# Child training in the Child ViReal Support Program: Combining iVR-based cognitive training and CBT techniques in a pilot study

**DOI:** 10.1371/journal.pone.0343364

**Published:** 2026-02-27

**Authors:** Iouliani Pachiti, Fotios S. Milienos, Timothy C. Papadopoulos, Panagiota Dimitropoulou

**Affiliations:** 1 Department of Psychology, University of Crete, Rethymnon, Greece; 2 Department of Sociology, Panteion University of Social and Political Sciences, Athens, Greece; 3 Department of Psychology & Center for Applied Neuroscience, University of Cyprus, Nicosia, Cyprus; Taipei Veterans General Hospital, TAIWAN

## Abstract

**Introduction:**

Attention deficits are common among school-aged children and affect their social, academic, and family lives, making it necessary to receive adequate support and intervention strategies.

**Objectives:**

This study aimed (a) to evaluate whether a multilevel intervention program –combining parent training, child training based on a cognitive-behavioral approach, and iVR-based cognitive training—could improve attention, executive functions, and psychosocial adjustment in children with attention deficits, and (b) to examine whether outcomes differed according to the sequence in which the intervention components were delivered. This pilot study was conducted as a precursor to a larger study.

**Methods:**

Sixteen families were randomly assigned to two groups following a sequential intervention design, with each group receiving the same components in a different order. Hence, families in the PC group received parent training first, followed by child training, while families in the CP group began with child training, followed by parent training (Clinical Trials Registry NCT05391698).

**Results:**

After participating in the intervention program, children demonstrated enhanced attentional and inhibitory control and sustained attention, as measured by fewer omission and commission errors, faster hit reaction time, and improved executive score. They also exhibited gains in working memory, processing speed, and planning and programming abilities and reported reduced behavioral problems. Emotional competence improved significantly only for the PC group. A significant time X group interaction was found for school competence, with the PC group showing a more pronounced improvement over time than the CP group. In contrast, no significant changes were observed in alerting and orienting scores, reaction time variability, social competence or self-perception skills.

**Conclusions:**

The findings have important implications, suggesting that multilevel intervention programs – particularly those integrating iVR-based cognitive training – may positively impact specific cognitive and psychosocial outcomes in children with attention deficits. As a pilot study, these results provide preliminary evidence and methodological insights to guide the design and rationale of future larger-scale studies.

## Introduction

Attention is a vital cognitive function that enables individuals to focus on important stimuli while filtering out distractions. It is crucial for sustaining focus, adapting to changes, and controlling impulses [1–3]. Variations in attention occur across individuals and developmental stages, especially in children whose attentional capacity is influenced by development [4,5]. Hence, attention impacts various domains of human functioning, including cognitive development, social interactions, and academic success.

However, many school-aged children experience attention deficits, affecting their academic, social, and emotional well-being [6]. These deficits stem from genetic, neurological, and environmental factors, resulting in various cognitive challenges [7,8]. Children with attention deficits often struggle with working memory, sustained attention, visual processing speed, response inhibition, and planning/programming abilities compared to typically developing peers [9–12]. These challenges frequently extend to secondary deficits such as difficulties in emotional regulation, social relationships, and academic performance [13–15], which may lead to further adverse effects on the parenting of children with ADHD.

Indeed, the challenges associated with attention deficits/ADHD can significantly disrupt family dynamics. It has been reported that parents or caregivers of children with attention deficits often struggle to manage parenting responsibilities and typical family routines, resulting in increased stress and reduced parental self-confidence, undermining their well-being and relationships [16,17]. Elevated stress levels could also lead to disrupted parent-child interactions and the use of more authoritarian or permissive parenting practices, which are associated with worsened ADHD symptoms and reduced response to interventions [18–20].

Considering the above, affected children and their families require targeted support and intervention to deal with daily adversities. Thus, the present study aimed to develop a multimodal intervention program designed to help children with attention deficits and their parents address these challenges. Additionally, the study aimed to examine the possible efficacy of this program in improving the children’s cognitive, behavioral, and psychosocial measures and reducing parenting stress while enhancing parental self-efficacy and parenting practices, as a groundwork for a larger study.

### Interventions for treating ADHD symptoms

Evidence-based interventions include pharmacotherapy, psychosocial interventions, or a combination of both [21,22]. While pharmacotherapy has been found to reduce the core ADHD symptoms (i.e., inattention and impulsivity/ hyperactivity) [23,24] and lead to short-term improvements in children’s social interactions and school performance [25,26], concerns about side effects and long-term sustainability remain [27,28]. Moreover, children require training to develop social and emotional skills, and parents should also receive parallel training to support their children and promote their well-being. It becomes evident that pharmacotherapy alone is insufficient to manage ADHD symptoms and their consequences.

Given the limitations of pharmacotherapy, there is a growing focus on psychosocial interventions to address ADHD symptoms more comprehensively. Psychosocial interventions, including behavior modification programs and child training programs based on cognitive-behavioral therapy (CBT), provide comprehensive approaches to manage attention deficits/ADHD by addressing both child behavior and parent-child interactions [7,29]. The CBT approach is based on the core idea that thoughts, emotions, and behaviors are interconnected, and by addressing one element (e.g., unhelpful thoughts), it is possible to influence and improve the other elements (e.g., emotional regulation and behavior). Hence, child training programs focus on helping children understand the connections between their thoughts, emotions, and behavior, equipping them with skills like self-instruction, self-evaluation, problem-solving, and self-reinforcement. Through role-playing and practical exercises, children learn to self-regulate, adapt to daily routines, and foster healthy relationships while improving self-esteem and emotional regulation [30,31].

Likewise, parent training programs help parents understand their children’s challenges and learn effective strategies to reduce undesirable behaviors while promoting positive ones based on principles from operant conditioning and social learning theories [7,29]. These programs emphasize reinforcing desirable behaviors, setting clear rules, and employing contingency management systems to replace dysfunctional interaction patterns with positive ones [22,32]. More recently, mindful parenting and mindfulness-based techniques have also been incorporated into these programs, further improving emotional regulation and enhancing parent-child interactions [33,34].

Hence, psychosocial interventions provide essential skills to children and parents for behavior management, self-control, problem-solving, and social interactions [30,35]. Additionally, parental participation in such programs correlates with increased parental self-confidence, improved parenting practices, reduced stress and negative emotions, and enhanced parent-child relationships [35–37].

In the last few years, new methods of intervention, such as cognitive training, have emerged for children with attention deficits. Specifically, cognitive training interventions target deficient neurocognitive functions like sustained attention, inhibitory control, and working memory through intensive repetition, steady increase in cognitive load, and feedback [38–40]. These interventions, often delivered through gamified electronic interfaces, have been promising in enhancing sustained attention and reducing impulsivity and hyperactivity [41–43].

### Immersive virtual reality (iVR)

Recently, interest has shifted towards using immersive virtual reality (iVR) technology for cognitive training, as it offers unique advantages over traditional interventions. iVR enables the creation of realistic, interactive, and gamified environments that closely resemble real-life scenarios, thereby enhancing ecological validity and supporting the transfer of newly acquired skills to daily life compared to traditional methods, such as two-dimensional (2D) cognitive training or child training programs in a therapist’s office [44–46].

Key features of iVR, such as presence and agency – allowing children to feel immersed in and in control of their virtual surroundings – are particularly effective in maintaining engagement and fostering attention [47]. For instance, iVR environments can replicate familiar contexts, such as a classroom, where typical distractors (e.g., peers talking or environmental noises) are introduced, enabling targeted attention training in a controlled and adaptive manner. Additionally, the flexibility of iVR allows for personalized feedback, task repetition, and the dynamic adjustment of training complexity to suit individual needs [48]. Hence, children can engage in immersive interactions and repeat tasks until mastering the required skill while progress is continuously monitored [49,50].

Recent meta-analyses and reviews highlight the benefits of iVR-based cognitive training for children with attention deficits, showing improvements in sustained attention [51–53] and working memory [54,55], with promising results for reduction in impulsivity and hyperactivity symptoms [56,57].

### The present study

Considering all the above, previous studies examining the effects of intervention programs on families of children with attention deficits have some limitations. First, to our knowledge, no prior studies have examined the combination of parent and child training using iVR technology for children’s cognitive training. The research in the field has primarily focused on parent training programs [37,58,59], child training programs [60–62] or a combination of both approaches [30,63,64]. Additionally, studies involving innovative technologies like iVR for cognitive training of children with attention deficits have predominantly used them as a standalone intervention delivery method [51,53,57] without integrating evidence-based psychosocial interventions. Second, no previous studies have examined the differential effects of a parent training program versus a child training program using iVR-based cognitive training on parents’ and children’s outcomes. The present study therefore integrates three approaches: parent training, child training, and iVR-based cognitive training. By incorporating iVR-based cognitive training in this manner, the study seeks to blend evidence-based intervention approaches with cutting-edge technology to enhance the outcomes for children with attention deficits and their families.

Hence, the rationale for combining these components was threefold. First, CBT-based interventions, as previously noted, have consistently demonstrated effectiveness in enhancing emotional regulation, behavioral functioning, and social skills in children with attention deficits – particularly when parent involvement is included [65]. Second, cognitive training programs – especially those delivered through engaging and ecologically valid platforms such as iVR – have been associated with improvements in attentional control, working memory, and executive functioning [38,66]. Third, emerging evidence supports the use of integrated multimodal interventions that combine psychosocial and cognitive elements, as they may produce synergistic effects and offer more holistic benefits by addressing both behavioral and neurocognitive dimensions of attention-related difficulties [14].

Building on these findings, the present study aimed to evaluate the potential impact of a comprehensive, multi-level intervention program – combining parent and child training with iVR-based cognitive training – on children’s cognitive functions (attention, inhibition control, working memory, processing speed, and programming skills) and psychosocial adjustment (social, emotional, and school competencies, self-perception, and behavioral problems). Additionally, the study aimed to explore the differential effects of intervention sequencing (PC group vs. CP group) in order to provide a more nuanced assessment of the program’s combined and component-specific impact. The measures used to evaluate the outcomes are described in detail in the Methodology section.

Based on the findings of previous endeavors regarding the effects of child cognitive training programs on children’s *cognitive functions* [39,52,67], we anticipated that at the end of the intervention program (Time 3) and compared to their initial assessment/baseline (Time 1), children would:

1. demonstrate enhanced performance in sustained attention, focused attention, and attentional control. Hence, we expected reduced (a) omission errors, (b) hit reaction time (hit RT), and (c) reaction time variability (RTV) from the Go/NoGo task and lower (d) alerting, (e) orienting, and (f) executive scores from the Attention Network Task (ANT task).
2. have better inhibition control as measured by reduced commission errors from the Go/NoGo task.
3. show (a) improved performance in planning and programming skills as reflected by better results in the Tower task; (b) enhanced working memory, and (c) better processing speed.

Moreover, based on the positive results from intervention programs using CBT-based techniques for the development and enhancement of *social, emotional, and behavioral skills* in children with attention deficits [30,60,68], we expected that after participating in the intervention program (Time 3) and compared to their initial assessment (Time 1), children would:

1. report increased social, school, and emotional competencies, as well as improved self-perception skills.
2. report fewer behavioral problems than in their initial assessment.
3. We also anticipated that the positive results would be maintained at the follow-up assessment four months after the end of the intervention program (Time 4). Hence, we anticipated that children (a) would perform better in the cognitive tasks (Go/NoGo task, ANT task, working memory, processing speed, Tower task), showing enhanced attentional control, sustained attention, inhibitory control, working memory, processing speed, and planning/ programming skills; (b) would report higher levels of social, school and emotional competencies, and self-perception skills, and (c) would report lower levels of behavioral problems.
4. Finally, at the Time 2 assessment, we expected that children in the CP group (child training before parent training) would demonstrate better cognitive outcomes compared to those in the PC group (parent training before child training), as the CP group would receive direct iVR-based cognitive training earlier in the intervention, whereas the PC group would first undergo parent training.

## Methodology

### Participants

#### Eligibility criteria for participants.

Families were eligible for inclusion in the study based on the following criteria: (a) the child had a formal diagnosis of ADHD, (b) the child was between 9 and 12 years old, (c) the child exhibited a Full-Scale IQ equal to or above 80, as assessed by the WISC-V [69], (d) both parents and the child were able to communicate effectively in the Greek language, (e) the child did not exhibit any comorbid disorders or concurrent difficulties that could potentially interfere with task performance during assessments or the intervention program (e.g., autism spectrum disorder, pervasive developmental disorder, visual or hearing impairment, psychotic disorder), (f) neither the child nor the parents had previously participated in an intervention program based on the behavioral or CBT approach, and (g) the child was not undergoing any medication treatment for ADHD.

#### Participant recruitment.

The research team collaborated with professionals from the official Interdisciplinary Evaluation, Counseling, and Support Centers (KE.D.A.S.Y.) in Heraklion and Rethymnon in Crete, Greece, to recruit families for the study. The staff at the centers informed parents with children diagnosed with ADD/ADHD about the opportunity to participate in the study. Interested families could phone the research team or complete a participation form on the program’s website (https://sites.google.com/view/childvireal/home) and the recruitment period lasted from 01.01.2022 to 01.03.2022. The research team contacted families meeting screening inclusion criteria, and written informed consent was obtained independently from parents and children. Also, parents provided a copy of their child’s assessment report validating the primary clinical diagnosis (ADD/ADHD) and completed questionnaires covering sociodemographic variables, the child’s characteristics, and family and other contextual factors. Additionally, children underwent a WISC-V assessment to evaluate their Full-Scale IQ [69]. Eligible families were subsequently invited to participate in the intervention program. The study adhered to the Consolidated Standards of Reporting Trials (CONSORT) guidelines (see S1 CONSORT Checklist) [70]. In addition, the study was approved by the Research Ethics Committee of the University of Crete (REC-UOC) (Approval Reference no. 51/25.02.2020). Participants were given an information sheet and access to the online website to review study details at their preferred pace. Also, written informed consent was reaffirmed before the implementation of the intervention.

#### Intervention procedure.

The *Child ViReal Support Program* is a comprehensive multi-level intervention program integrating child training and parent psychoeducational training [71]. The study’s principal investigator, a licensed School Psychologist, developed the training manuals. They were then reviewed by one of the coauthors, who has extensive expertise in school psychology, to ensure consistency in the exercises and informational material provided to participants. Additionally, two other licensed Educational/School Psychologists extensively reviewed the material and provided feedback before this was finalized. These two collaborators were also trained to utilize the manuals and deliver the training programs. The research team held weekly supervision sessions with the facilitators to ensure the intervention quality and fidelity and to provide ongoing support and guidance.

#### Child training program.

Children participated in a structured, in-person training program consisting of 16 individualized one-hour sessions held twice a week for eight weeks. Utilizing iVR technology, the program aimed to enhance sustained attention in a virtual classroom setting, supplemented by CBT techniques to promote behavioral and emotional self-regulation skills. The program facilitators followed the manual guidelines to ensure fidelity and completed an Integrity Checklist after each session to evaluate the coverage of session topics. In the rare event of a missed topic (e.g., due to time constraints), it was addressed at the beginning of the subsequent session. Also, measures were taken to reschedule missed sessions (e.g., due to illness), ensuring that all participating children would fully benefit from the program and complete an equal number of sessions.

The components of the child training program are described below.

**Cognitive-behavioral approach**. The program integrated established CBT techniques, extensively documented in previous studies [30,31,72,73]. These techniques aim to provide children with a comprehensive understanding of the interplay between emotions, thoughts, and behaviors.

The sessions were designed with progressive goals and targeted specific themes to support the development of essential skills for children with attention deficits/ADHD using interactive methods such as role-playing, storytelling, modeling, painting, and educational board games. During these sessions, children were trained to clarify the connection between emotions, thoughts, and behaviors and to identify and transform adverse automatic thoughts into more adaptive, positive alternatives. They were also trained to recognize situations that hinder concentration and to develop strategies for managing distractions effectively. Additionally, they were taught techniques to identify and express pleasant and unpleasant emotions, to manage challenging emotions (e.g., anger, stress) effectively, and to cultivate self-control and manage impulsivity. Moreover, the sessions focused on enhancing social competencies (e.g., initiation and maintenance of conversations, conflict resolutions), and using effective problem-solving strategies. Children were further supported in developing goal setting, time management, and executive functioning skills through structured activities. They practiced self-observation, self-guidance, and self-reinforcement techniques to foster independence and internal motivation. Finally, to reinforce skill acquisition and generalization to real-life settings, homework assignments were provided, encouraging children to apply what they learned in their home environments. A detailed overview of the session structure and thematic content is provided in S1 Appendix.

**Virtual classroom.** For the current study, a virtual classroom was created to replicate a real-world classroom experience using an HMD inspired by Rizzo and colleagues’ virtual classroom task [74,75]. The environment included three rows of desks with seated avatars representing students and a teacher avatar at the front. Visual aids like pictures on the wall and a whiteboard enhanced authenticity, with a door and window providing a view of the schoolyard. Children engaged in two tasks within the virtual classroom: the Card Sorting task and the Continuous Performance Task (CPT). Both tasks had a progressive structure with three levels (Levels 1, 2, and 3), each offering two levels of difficulty (Easy and Difficult). In each session, children were seated with the HMD appropriately adjusted for optimal viewing and effective task performance. Based on the program guidelines and their progress, they executed one of the two tasks.

During the Card Sorting task, children had to sort cards presented on their virtual desk based on specific criteria (e.g., numbers). For instance, if a card with the number 3 appeared, the child had to place it in the correct pile labeled with the number 3. The speed of card appearance increased as the task progressed, aiming to enhance attentional focus and minimize distractibility. In the CPT task, children watched stimuli appear on the virtual whiteboard. They had to respond to certain stimuli (GO stimuli, e.g., fruits) by pressing a button on the controller while ignoring others (NoGO stimuli, e.g., monkey) by refraining from reacting. The time between the appearance of target stimuli increased as the task progressed, challenging children to maintain their attention for extended periods, aiming to train vigilance and response inhibition. S2 Appendix illustrates and presents the parameters of both tasks.

Throughout the tasks, children encountered various distractors, including auditory (e.g., dropping pencils), visual (e.g., front-row classmates raising hands), and mixed (combining audio and visual, for example, a teacher passing by an open door and footsteps). Sessions became more challenging as distractors increased, and the duration of tasks extended from 20 to 30 minutes. This escalating cognitive load provided children with progressive training to resist distractions and maintain concentration. Additionally, a reward system allowed children to play a bonus game from the HMD’s application library (e.g., bowling, tennis) for accomplishing a session’s goal (e.g., accurately sorting a set number of cards).

The children experienced the virtual classroom using the standalone Oculus Quest 1 (Meta Platforms and Technologies, California, US), equipped with two Oculus Touch controllers for precise hand tracking and interaction within the virtual environment, and wore headphones for an enhanced immersive experience. The virtual classroom was developed using the Unity3D game engine (version 2019.4; Unity Technologies, San Francisco, CA, US), with additional functionality integrated using the C# programming language. In the first session, children were familiarized with the HMD and iVR technology by engaging in two training exercises, one for each of the described tasks and a bonus game.

#### Parent psychoeducational training program.

Parents participated in a structured psychoeducational training program designed to address ADHD-related challenges, drawing insights from established parent programs [32,76] and neuropsychological research on children with attention deficits and neurocognitive training [38,77,78]. The program consisted of eight weekly group sessions, each lasting 1.5–2 hours, covering specific thematic topics and using various instructional methods, such as lectures, group discussions, modeling, and role-playing, to enhance engagement and understanding. During the program, parents were introduced to attention deficits, associated executive function deficits, and behavioral challenges faced by children with ADHD in home and school environments. Additionally, they were trained in evidence-based strategies, including mindfulness-based stress management, behavior modification, effective communication techniques, and methods to enhance their children’s executive functions, social skills, and emotional regulation. Parents had to complete weekly homework assignments to practice newly learned skills within their family context. A more detailed description of the parental psychoeducational training program is available elsewhere [71].

#### Trial design and randomization.

A pilot study with a sequential intervention design was conducted to evaluate the potential effectiveness of the *Child ViReal Support Program* involving 16 children (*M* = 10.48, *SD* = .94, two girls) diagnosed with ADHD and their parents. The families were randomly assigned to either the PC group (parent training followed by child training; *n* = 9) or the CP group (child training followed by parent training; *n* = 7) using simple randomization via a web-based random number generator. No stratification or block randomization was applied due to the small sample size and pilot nature of the study. Group allocation was concealed until the moment of assignment. This sequencing was implemented to assess the differential impact of parent and child training programs on the outcomes and to provide a comprehensive assessment of the combined intervention program. Outcome assessments at all time points were conducted by an assessor who was aware of the participants’ group allocation but was blinded to the specific hypotheses of the current study.

**Fig 1:**
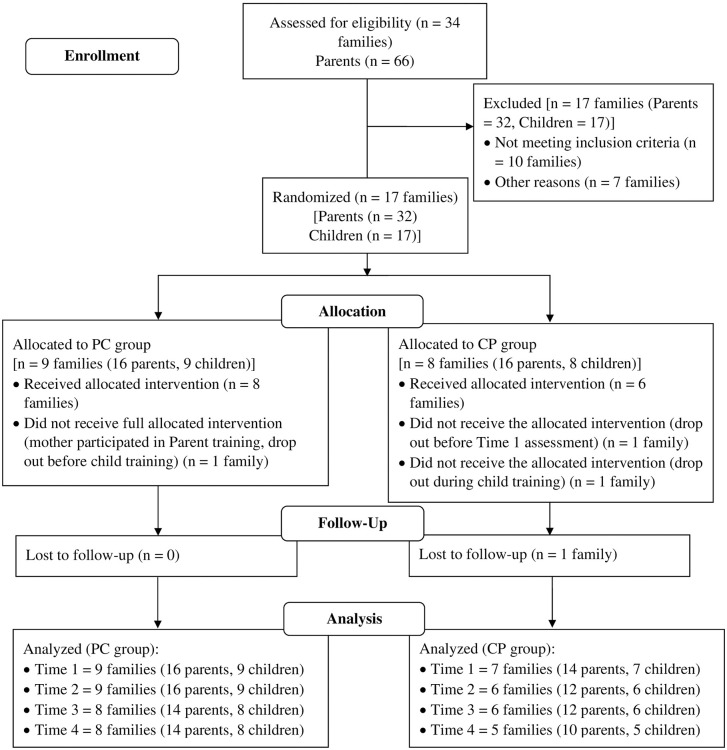
Participant flow diagram.

Two families withdrew from the intervention program throughout the study for personal reasons. In the first case, a family initially assigned to the PC group completed the parent training program but withdrew after the Time 2 assessment. In the second instance, a family initially assigned to the CP group dropped out during the child training before the Time 2 assessment. Additionally, one family could not participate in the follow-up assessment (Time 4), conducted four months after the end of the intervention program. A visual representation of the recruitment and randomization processes is shown in Fig 1. The study protocol was registered in the Clinical Trials Registry (NCT05391698) [79].

### Study procedure

**Fig 2:**
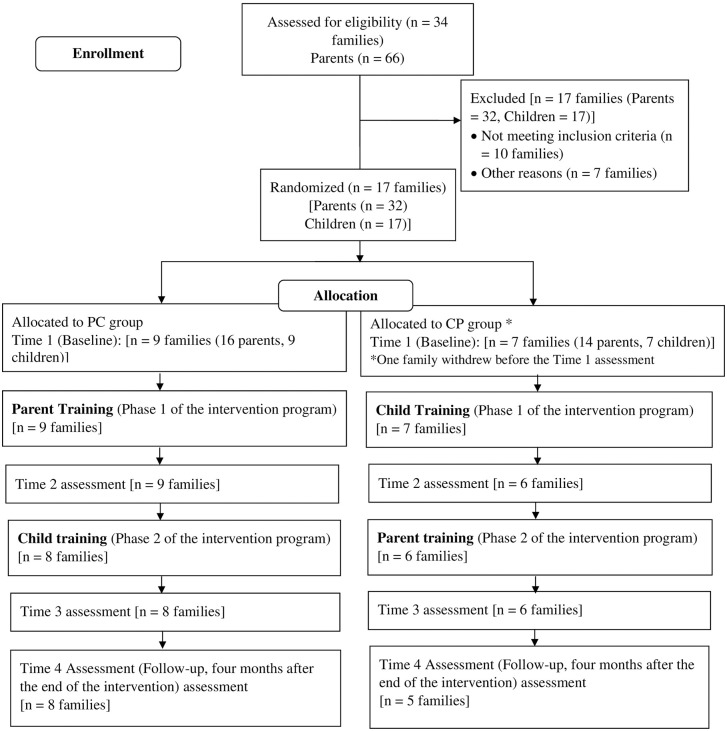
Intervention procedure flow diagram.

The study employed a longitudinal design with four assessment time points. Initially, all participants underwent a baseline assessment (Time 1 in March 2022) before being randomly assigned to either the PC or CP groups. The first phase of the intervention program lasted eight weeks, during which the PC group received parent training, and the CP group engaged in child training. Then, a second assessment (Time 2 in May 2022) was conducted using the same measures as the baseline. Participants then transitioned to the second phase of the intervention, lasting approximately another eight weeks, where the PC group received child training, and the CP group received parent training. Upon completion of the intervention program, a third assessment (Time 3 in July 2022) was conducted using the same measures. A follow-up assessment (Time 4 in November 2022) occurred four months after the Time 3 assessment, during which participants completed the same measures. A visual representation of the intervention procedure is shown in Fig 2.

### Measures

**Attention Network Task** (ANT; 80,81). The ANT task is a widely used cognitive task to measure attention in both children [80] and adults [81]. It evaluates the three attention networks described in Posner’s attention model: the alerting network, the orienting network, and the executive control network [82–84]. During the assessments at the four time points (T1, T2, T3, T4), children executed the child-adapted version of the ANT task called “Feed the Hungry Fish!” [80] using Psychopy software [85] on an MSI computer. Each trial started with a fixation cross at the center of the screen for a variable duration of 400–1600 milliseconds, followed by attentional cues that appeared in four conditions (no cue, central cue, double cue, or spatial cue) for 150 milliseconds, providing information about the target’s location. The target stimulus (fish) appeared alone or with four additional fish (flankers) above or below the fixation point. The flankers could point in the same (congruent) or different (incongruent) directions as the target. Children could indicate the direction of the target fish using the arrow keys (right or left) within a time frame of up to 1700 milliseconds, after which they received visual and auditory feedback. The task included 24 practice trials and three blocks of 48 trials each, evenly distributed across 12 conditions, combining the three target types and the four cue types. The task duration was approximately 20 minutes, with breaks permitted between blocks. Reaction times and response accuracy were recorded for each participant, and the three attention network scores were derived from reaction time differences across various stimulus combinations.

**Continuous Performance Task (CPT).** The Go/NoGo task is a variant of CPT tasks. It is widely used in psychological and neuroscience research, particularly in studying attention, cognitive control, and response inhibition, especially in clinical populations such as individuals with ADHD [86,87]. In this study, children executed a custom-designed Go/NoGo task inspired by Conners CPT-III [88] using Psychopy software [85] on an MSI laptop. During the task, children viewed alternating visual stimuli on the computer screen with varying inter-stimulus intervals (ISI) of 1, 2, or 4 seconds. They were instructed to respond to target stimuli (Go trials, 80% of the trials) by pressing the spacebar while inhibiting their response to non-target stimuli (NoGo trials, 20% of the trials). Immediate feedback was provided via happy or sad stars, indicating response accuracy. The task included 20 practice trials and six experimental blocks of 60 trials each (360 trials), lasting approximately 14–15 minutes, with breaks allowed between blocks.

Results were analyzed for (a) *omission errors* (i.e., the number of cases where a response did not occur in the presence of a target), which indicate inattention, (b) *commission errors* (i.e., the number of cases where a response occurred in the presence of a non-target) measuring failed response inhibition, (c) *mean reaction time* (hit reaction time; hit RT), which is the average time to respond to target stimuli and (d) *variability of the hit reaction time* (RTV), calculated as a coefficient of variation resulting from the division of standard deviation of reaction time from the mean reaction time [89,90]. RTV is a key indicator of cognitive performance associated with attention lapses, with higher RTV usually observed among individuals with ADHD, possibly indicating difficulties in attention control or the ability to maintain focus [91].

**Tower Task** [92,93]. The Tower Task, part of the “Test of Executive Functions in Elementary School” [92], was adapted from the Tower of London (ToL) test [94]. This test evaluates executive functions related to the prefrontal cortex, particularly planning and programming abilities, cognitive control, and problem-solving in non-verbal contexts for both children and adults [92,95,96]. It has been utilized extensively in neuropsychological assessments across clinical and non-clinical samples [97].

In the current study, children executed the computerized version of the task using a mouse to relocate three colored cylinders to target positions within a specified number of moves and a 60-second timeframe, following visual examples. Hence, success in the task relied on planning and executing actions while avoiding impulsive trial-and-error approaches. The Tower task has demonstrated good internal consistency (*a* = .78) and test-retest reliability over two weeks (*r* = .99) [92,93]. Additionally, children with impulsivity/ hyperactivity traits exhibited lower performance than their typically developing peers, supporting its concurrent validity [98].

**Test of Psychosocial Adjustment** [99,100]. This test evaluates social and emotional competencies or deficits in children aged 4–12. It can identify difficulties in interpersonal and intrapersonal adjustment, assess psychosocial characteristics of children with learning difficulties, predict factors for academic progress, and provide insights into various domains of psychosocial competence that contribute to a child’s mental resilience when dealing with learning difficulties at school. Additionally, it can be used to assess the effectiveness of intervention programs at individual or group levels [99].

The test comprises three scales: the “Educator Scale for Preschool Children (4-6 years)” and “Educator Scale for School-Age Children (7-12 years)” completed by educators, and the “Self-Report Scale”, completed by school-aged children. The “Self-Report Scale” used in this study consisted of 115 items measuring behavior frequency on a 5-point Likert-type scale (1 = Not at all, 2 = A little, 3 = Moderately, 4 = Quite a lot, 5 = Very much). It included five subscales, each focusing on specific dimensions. The *‘Social Competence’* (*n* = 25 items) evaluated assertiveness/leadership skills, interpersonal communication, and peer cooperation. The *‘School Competence’* (*n* = 30 items) assessed motivation, planning/ organization, and school effectiveness to determine a child’s adaptation to the school environment. The *‘Emotional Competence’* (*n* = 18 items) measured self-control, emotion management, and empathy. The *‘Self-Perception’* (*n* = 26 items) examined the child’s school-related perception of their academic abilities, performance, and overall self-image. The *‘Behavioral Problems’* subscale (*n* = 16 items) focused on maladaptive behaviors in the school context that affect the child’s social, emotional, and overall psychosocial adjustment. Higher scores across the first four subscales indicate positive behaviors and good adaptation, whereas higher scores within ‘Behavioral Problems’ suggest significant behavioral problems and concentration difficulties.

The test’s validity is supported by representative content validity through a comprehensive literature review, a theory-based question bank, expert revisions, and pilot study results [99,100]. Furthermore, internal consistency reliability for the five factors/subscales falls within an acceptable range (from *a* = .65 to *a* = .89) based on the normative data [99,100] and recent study results [101] demonstrating the reliability and validity of the test.

**Working Memory Index** (WMI, WISC-V;,69)**.** The assessment of children’s working memory utilized the Working Memory Index (WMI) from the WISC-V test [69]. The WMI assesses the capacity to temporarily register, maintain, and manipulate visual and auditory information, requiring attention, concentration, and discrimination. It consists of two subtests: Digit Span and Picture Span. In the Digit Span, the child is required to recall numbers in the same (forward digit span), in reverse (backward digit span), or in ascending order (sequencing digit span). In the Picture Span, the child must memorize and identify pictures, preferably in sequential order. Normative data obtained from the Greek version of WISC-V-GR [102] indicate acceptable split-half reliability for Digit Span (*r* = .91) and Picture Span (*r* = .88) and high overall reliability for the WMI (*a* = .93)*.*

**Processing Speed Index** (PSI, WISC-V;,69). Children’s processing speed was evaluated using the Processing Speed Index (PSI) from the WISC-V test [69]. This index measures how quickly and accurately a child can process visual information, evaluating visual perception, attention, and cognitive task execution speed. It comprises two subtests: Coding and Symbol Search. In the Coding subtest, the child uses a key to copy symbols corresponding to numbers within a time frame. In turn, the Symbol Search subtest requires the child to identify if a target symbol is present within a group of symbols within a specified time frame [69]. Normative data from the WISC-V-GR [102] indicate good test-retest reliability for both the Coding (*r* = .84) and the Symbol Search (*r* = .83) subtests. Additionally, the PSI exhibits strong overall reliability with a coefficient of.89.

### Feasibility and acceptability measures

We developed two bespoke questionnaires to evaluate the child training program. At the end of the intervention, each participating child completed the “Evaluation of Child Training-Final” (ECT-F) and the “Evaluation of VR Experience-Final” (EVR-F) questionnaires to assess their overall experience and engagement with the training program and their acceptance of iVR technology (equipment and tasks).

**Evaluation of Child Training – Final (ECT-F):** The ECT-F questionnaire, comprising 27 items, drew inspiration from reputable instruments known for their reliability and validity in assessing children’s satisfaction with intervention programs. Specifically, some items of these instruments were adapted into Greek through the back-translation method [103–105] by two independent bilingual psychologists whose outcomes matched the original version. It is categorized into three subscales. The *Structure/Content* subscale contains 14 statements derived from the Youth Client Satisfaction Questionnaire (YCSQ; 106) for which children used a 4-point Likert-type scale (0 = Not at all, 1 = A little, 2 = Moderately/Several, 3 = Very much) to evaluate satisfaction and enjoyment with various aspects of the program. Shapiro and colleagues [106] identified two factors within this subscale. The first factor, ‘Relationship with the Therapist/Trainer’ (6 items, e.g., *‘My trainer cared about me’*), assesses the child’s enjoyment, fulfillment throughout the program, and perception of their trainer’s empathy and attentiveness. The second factor, ‘Benefits of Therapy’ (8 items, e.g., *‘During the program, I learned things that helped me’*), evaluates whether changes occurred in emotions, behavior, family dynamics, and the child’s perception because of the intervention program. The total subscale score (*Total Satisfaction Score*) can range from 0 to 42, indicating the child’s overall satisfaction.

The *Utility/Feasibility* subscale includes 11 items adapted from elements of the Children Usage Rating Profile (CURP; 107) and aims to evaluate participants’ perspectives on the program’s perceived utility, feasibility, and acceptance. Responses were collected on a 4-point Likert scale (1 = Strongly disagree, 4 = Strongly agree) and explored the children’s views on the program’s content, homework, and how well it aligned with their needs. Two distinct factors emerge based on its structure and items adopted from the CURP [107]. The score for the ‘Utility/Feasibility’ factor (6 items, e.g., *‘The program and the sessions were too much work for me’*) evaluates whether children found the program beneficial and suitable considering the time and effort invested. The ‘Acceptance’ factor (5 items, e.g., *‘I could see myself participating in this program again’*) assesses whether children enjoyed participating and would be interested in participating again if given the opportunity.

The *‘Your Opinion’* section of the ECT-F includes two open-ended questions that allow children to provide feedback on what they liked most and least about the sessions and activities. This offers valuable qualitative insights into the participants’ perspectives and experiences.

**Evaluation of VR Experience – Final (EVR-F):** The EVR-F questionnaire, created for the current study, aimed to evaluate the children’s overall experience with VR, including the utility, feasibility, and acceptance of iVR technology (tasks and equipment). Comprising 32 items, the EVR-F was divided into five subscales. The *Cybersickness Symptoms* subscale (7 items) was drawn from established questionnaires such as the Child Simulator Sickness Questionnaire [Child SSQ;, [108] and the Pediatric Simulator Sickness Questionnaire [Peds SSQ;, 109] to assess participants’ susceptibility to cybersickness symptoms while engaging with iVR technology. Children used a 3-point Likert-type scale (0 = No, 1 = A little, 2 = A lot) to rate symptoms, which are categorized into three subgroups: *nausea* (e.g., *‘Do you feel sick?’*), *oculomotor discomfort* (e.g., *‘Do your eyes hurt?’*) and *disorientation* (e.g., *‘Are you dizzy with your eyes open?’*). Each subgroup contained three items, yielding scores from 0 to 6. A total score of 3 or more points in any subgroup indicates the presence of cybersickness symptoms, prompting the trainer to remove the child from the experimental setting (i.e., take off the HMD) until symptoms are alleviated.

The *Sense of Presence* subscale inspired by the Sense of Presence [SofP; 110] and the Augmented Reality Immersion [ARI; 111] questionnaires measures participants’ sense of immersion and presence in the virtual classroom environment. Children responded to a 3-point Likert-type scale (0 = No, 1 = Somewhat, 2 = A lot) on ten items (e.g., *‘The environment and the items in the classroom made me feel like I was actually in the classroom’*). The total score (ranging from 0 to 20) reflects the child’s perceived level of presence/immersion within the virtual environment.

The *Enjoyment/Engagement* subscale (5 items) explores the children’s subjective experiences with iVR tasks, such as enjoyment and engagement. Drawing from elements from the Virtual Reality Neuroscience Questionnaire [VRNQ; 112], this subscale enabled participants to rate statements (e.g., *‘I liked the use of the VR glasses and the virtual tasks’*) on a 3-point Likert-type scale (0 = No, 1 = Maybe, 2 = Yes). The total score ranges from 0 to 10, with a higher score indicating greater satisfaction and enjoyment while using iVR technology, engagement in activities, and the child’s intention to use this technology again.

The *Usability of the iVR technology* subscale includes eight items adapted and modified from the VRNQ [112] and the System Usability Scale [SUS; 113, 114]. Children responded again on a 3-point Likert-type scale (0 = No, 1 = Somewhat, 2 = Yes) to rate the items that assess participants’ perceptions of the ease of use and functionality of iVR equipment and tasks (e.g.,*‘The VR glasses/HMD were comfortable’*).

Lastly, in the *Your Opinion* section, children answered two open-ended questions, providing qualitative feedback on their experiences with iVR technology. Hence, participants could share their likes, dislikes, and suggestions for improvement about using the HMD and engaging in virtual activities.

### Statistical analysis

Initially, the statistical analysis of the dataset involved the computation of descriptive statistics, including means, standard deviations, and correlations separately for each time point and group (CP and PC). To assess the equality of means across different time points, we chose Friedman’s two-way analysis of variance, which is appropriate for small sample sizes and non-normally distributed data, assuming independence between subjects and an ordinal or higher scale of measurement – assumptions that were met in our dataset. To study the effect of the independent variables (time, group, age) on our outcomes (attention, inhibition, programming, working memory, processing speed, and psychosocial adjustment), we used marginal models [115]. Marginal models are advantageous for analyzing population-averaged effects rather than subject-specific effects. They provide a flexible method for modeling mean responses and addressing correlations in longitudinal datasets, making them particularly appropriate for our data, which contains scores from four assessment points. The data analysis was conducted using SPSS 29.0 [116] and R-project [117].

## Results

### Demographics

The children that participated in the study were diagnosed with ADHD by child psychiatrists at the official Interdisciplinary Evaluation, Counseling, and Support Centers (KE.D.A.S.Y.) and the state Community Children and Adolescents Mental Health Centers of Heraklion and Rethymnon in Crete, Greece. The children ranged in age from 9 to 12 years (*M* = 10.48, *SD* = 0.94) with intelligence quotient (IQ) scores between 80 and 103 (*M* = 90.56, *SD* = 8.46). Their parents (14 fathers and 16 mothers), aged between 32 and 52 years old (*M* = 42, *SD* = 5.11), also participated in the study. Most parents reported a secondary educational level (60%), and 36.7% reported a tertiary one (Bachelor’s degree and Master’s/Ph.D degree). Regarding employment, 76.7% were involved in full-time jobs, 10% indicated part-time employment, and 13.3% were unemployed. Of the 16 participating families, two were single-parent households. Geographically, most families lived in an urban area (*n* = 10, 62.5%), with some living in semi-urban (*n* = 3, 18.8%) and rural areas (*n* = 3, 18.8%).

### Main analysis

In this exploratory pilot study, analyses addressed the two main aims: (a) to examine overall changes in cognitive and psychosocial outcomes over time across all participants, and (b) to explore whether changes differed accordingly to intervention sequence (PC vs. CP group). In line with these objectives, results are presented in three stages. First, we report within-group changes over time using Friedman’s two-way analysis of variance to examine whether cognitive and psychosocial outcomes improved following the intervention. Second, we present correlations between variables. Third, we describe the results of the marginal models evaluating the effects of time, group (PC vs. CP), and their interaction on each outcome, thereby assessing whether intervention sequence influenced changes.

#### Descriptive statistics and Friedman’s two-way analysis of variance.

The descriptive statistics for cognitive and psychosocial measures across the four assessment time points (Time 1–4) and separately for each group (PC and CP) are provided in Table 1. Friedman’s two-way analysis of variance was employed to evaluate the statistical significance of differences over time, adjusted with Bonferroni correction at a.05 level (the small sample size is a crucial factor that should not be overlooked when making comments about statistical significance). The following analysis examine whether each group demonstrated significant improvements over the intervention and follow-up period.

**Table 1:** Means (SD in parentheses) of children’s subscales, for each time point and group, along with Friedman’s two-way analysis of variance (testing equality of means across time).

Group	Scale	Subscale	Time 1 (n = 16)	Time 2 (n = 15)	Time 3 (n = 14)	Time 4 (n = 13)	d (effect size)
**PC**	**Attention Network Task (ANT)**	**Alerting Score**	32.78 (68.35)	42.56 (52.59)	4.13 (51.31)	34.00 (44.94)	−2.11
		**Orienting Score**	46.11 (77.22)	39.78 (48.69)	18.75 (38.60)	34.00 (62.71)	.17
		Executive Score**	149.78 (40.00)^1^	92.11 (71.20)^1,2^	69.50 (25.55)^2^	68.25 (28.90)^2^	2.33
	**Go/NoGo Task (GNG Task)**	Omission Errors*	14.00 (9.52)	11.22 (7.79)	9.25 (7.96)	9.00 (7.61)	.58
		**Commission Errors**	39.78 (20.54)	36.11 (13.00)	25.50 (13.11)	32.00 (15.41)	.43
		**Hit Reaction Time (Hit RT)**	437.89 (61.13)	452.00 (40.33)	421.38 (45.21)	414.13 (41.34)	.45
		**Reaction Time Variability (RTV)**	0.32 (0.06)	0.32 (0.05)	0.29 (0.06)	0.30 (0.07)	.31
	**WISC-V**	Working Memory (WMI)**	16.89 (3.88)	17.44 (3.28)	20.88 (4.67)	19.75 (4.06)	−.72
		Processing Speed (PSI)**	18.56 (3.32)^1^	20.44 (2.24)^1,2^	23.50 (2.13)^2^	22.50 (3.85)^1,2^	−1.09
	**Tower**	Total Score***	12.24 (1.37)^1^	13.44 (1.42)^1,2^	15.00 (1.60)^2^	14.46 (1.52)^2^	−1.53
	**Psychosocial Adjustment Test (PSA)**	**Social Competence**	48.89 (15.50)	46.67 (14.81)	54.88 (14.82)	52.13 (16.13)	−.20
		**School Competence**	35.11 (5.18)	36.56 (7.07)	40.50 (6.63)	40.50 (6.07)	−.95
		Emotional Competence*	40.89 (14.09)	40.67 (13.47)	48.00 (11.20)	47.00 (12.81)	−.45
		**Self-Perception**	38.11 (5.27)	35.89 (3.65)	41.13 (4.12)	38.25 (4.26)	−2.92
		Behavioral Problems*	61.11 (6.56)	57.00 (4.35)	54.75 (4.26)	53.87 (6.51)	1.10
**CP**	**Attention Network Task (ANT)**	**Alerting Score**	51.71 (66.16)	53.50 (38.56)	14.67 (20.06)	15.20 (32.75)	.70
		**Orienting Score**	58.29 (44.59)	9.00 (83.81)	45.83 (36.27)	13.00 (38.03)	1.09
		Executive Score*	125.29 (52.17)^1^	84.67 (28.48)^1,2^	46.00 (41.04)^2^	48.20 (43.57)^2^	1.60
	**Go/NoGo Task (GNG Task)**	Omission Errors*	14.14 (9.33)	8.00 (4.64)	3.83 (3.12)	3.00 (1.22)	1.67
		**Commission Errors**	39.71 (18.65)	34.83 (18.38)	30.67 (14.39)	27.40 (14.41)	.74
		**Hit Reaction Time (Hit RT)**	459.00 (57.78)	451.50 (60.79)	394.17 (25.61)	386.00 (11.11)	1.75
		**Reaction Time Variability (RTV)**	0.28 (0.03)	0.29 (0.03)	0.27 (0.04)	0.27 (0.04)	.28
	**WISC-V**	Working Memory (WMI)*	15.71 (4.27)	17.50 (4.63)	18.50 (4.68)	18.80 (2.16)	−.91
		**Processing Speed (PSI)**	14.86 (2.79)	17.67 (3.50)	19.17 (2.56)	21.00 (1.58)	−2.71
	**Tower**	Total Score*	13.14 (1.66)^1^	14.94 (1.27)^2^	15.27 (1.65)^1,2^	14.22 (0.43)^1,2^	−.89
	**Psychosocial Adjustment Test (PSA)**	**Social Competence**	47.29 (16.63)	54.00 (14.38)	51.00 (15.71)	49.00 (15.44)	−.11
		**School Competence**	37.43 (3.10)	38.33 (3.32)	35.67 (4.76)	36.20 (5.97)	.26
		**Emotional Competence**	40.71 (11.08)	47.83 (8.97)	44.00 (12.88)	44.00 (14.91)	−.25
		**Self-Perception**	33.57 (6.37)	37.83 (1.47)	36.33 (3.14)	37.40 (4.72)	−.68
		Behavioral Problems*	49.71 (6.79)^1^	45.67 (6.80)^1,2^	45.83 (6.01)^1,2^	44.40 (3.78)^2^	.97

*Note.* ^1,2^ means with the same or without superscripts have no statistically significant difference, according to the pairwise comparisons and Bonferroni adjustment using Friedman’s test. Adjusted p-values less than 0.05 were considered statistically significant.

*p < .05. **p < .01. and ***p < .001.

Hence, concerning *cognitive measures*, we hypothesized improvements in sustained attention, focused attention, and attentional control – particularly reductions in the scores from Go/NoGo task [omission errors (1a), hit RT (1b) and RTV (1c)] and the scores from ANT task [alerting (1d), orienting (1e), and executive (1f) scores]. A significant within-subjects improvement (on average) was observed in the executive score of the ANT task (i.e., a decrease shows improvement) for children of both groups, with a decrease from baseline (Time 1) sustained through Time 3 and Time 4 (PC: *χ*^*2*^_*F*_ (3) =15.76, *p* = .001, *d* = 2.33, and CP: *χ*^*2*^_*F*_ (3) = 10.68, *p* = .014, *d* = 1.60), supporting Hypotheses 1f and 6a. Additionally, omission errors in the Go/NoGo task significantly decreased in both groups (PC: *χ*^*2*^_*F*_ (3) = 9.73, *p* = .021, *d* = .58, and CP: *χ*^*2*^_*F*_ (3) = 9.00, *p* = .029, *d* = 1.67), aligning with our hypothesis mentioning that children will demonstrate enhanced sustained and focused attention (Hypothesis 1a).

However, our results did not fully support our first and sixth hypotheses since the alerting and orienting scores (Hypotheses 1d, 1e, and 6a) did not differ significantly across the four assessment time points for either group. In addition, the decrease observed in hit RT did not reach statistical significance, while the RTV remained stable over time for the children of both groups across the four assessment time points (Hypotheses 1b and 1c).

As far as inhibition control is concerned, we hypothesized reductions in commission errors (from the Go/NoGo task) for children of both groups. While a decreasing trend was observed, no statistically significant change occurred, so Hypothesis 2 was not supported.

Turning to children’s executive functions’ outcomes, our hypotheses (3a, b, c) were largely supported. Participants from both groups showed a statistically significant increase in working memory over the course of the intervention (PC: *χ*^*2*^_*F*_ (3) =11.95, *p* = .008, *d* = −.72, and CP: *χ*^*2*^_*F*_ (3) = 9.39, *p* = .025, *d* = −.91). Hence, as hypothesized (Hypothesis 3b), children exhibited enhanced working memory from before to after their participation in the intervention. Additionally, regarding children’s planning/programming skills, there was a significant increase in the total score of the Tower task for children of both groups (PC: *χ*^*2*^_*F*_ (3) =15.76, *p* = .001, *d* = 1.53, and CP: *χ*^*2*^_*F*_ (3) = 10.68, *p* = .014, *d* = −.89) across the four assessment time points, as hypothesized (Hypothesis 3a). Specifically, and according to pairwise comparisons, the children of the PC group demonstrated a consistent increasing pattern over time, particularly after the Time 2 assessment, while they maintained a significant increase between their initial assessment (Time 1) and the follow-up assessment (Time 4), aligning with Hypothesis 6a. In the CP group, children showed an initial significant increase in the total score of the Tower task from Time 1–2, followed by stabilization and a slight non-significant decrease at Time 4.

Moreover, children in the PC group demonstrated a significant increase in processing speed over the course of the intervention (*χ*^*2*^_*F*_ (3) =12.20, *p* = .007, *d* = −1.09). Specifically, higher processing speed was observed between their baseline assessment (Time 1) and the assessment at the end of the intervention program (Time 3), according to pairwise comparisons. In contrast, children in the CP group did not show any significant changes in the processing speed over time. This result aligns partly with our expectations (Hypothesis 3c) since only the children from one of the two groups exhibited significant differences in processing speed after their participation in the intervention program.

Regarding the *psychosocial outcomes*, we anticipated increases in social, emotional, and school competencies and self-perception (Hypotheses 4 and 6b), while we hypothesized decreases in behavioral problems (Hypotheses 5 and 6c). Children in both groups exhibited a statistically significant decrease in behavioral problems over the intervention period (PC: *χ*^*2*^_*F*_ (3) = 9.78, *p* = .021, *d* = 1.10, and CP: *χ*^*2*^_*F*_ (3) = 10.47, *p* = .015, *d* = .97), as hypothesized (Hypothesis 5). This significant decrease was maintained at the follow-up assessment conducted four months after the end of the intervention, aligned with our expectations (Hypothesis 6c). Concurrently, children in the PC group showed a significant increase in emotional competence (*χ*^*2*^_*F*_ (3) = 10.04, *p* = .018, *d* = −.45), especially after participating in the child training program, which was still present at the follow-up assessment. The CP group showed a non-significant increase. The results regarding within-group changes in social competence, school competence, and self-perception did not meet our expectations (Hypotheses 4 and 6b); even though positive trends were observed in the means of both groups during the intervention period, these increases did not reach statistical significance.

In summary, both groups showed significant improvements in several cognitive domains, including executive function, sustained attention, working memory, and planning/programming abilities, alongside reductions in behavioral problems. Additionally, processing speed and emotional competence improved significantly only in the PC group. In contrast, no significant changes were observed in alerting and orienting scores, school and social competence, or self-perception.

#### Correlations.

The Pearson correlations between the study variables were computed separately for each time point and group (CP and PC), acknowledging the need for cautious interpretation of significance due to small sample sizes (see S1 Table). In the ANT task scores (alerting, orienting, and executive), both groups initially showed negative correlations between the orienting and both the alerting and executive scores at Time 1, shifting to positive correlations by Time 4. Also, the CP group showed non-significant positive correlations between executive and alerting scores and the PC group had non-significant negative correlations at all time points of assessment.

Regarding the scores from the Go/NoGo task, the PC group showed a shift in correlation between omission and commission errors from a negative at Time 1 (*r* = −.25) to a positive at Time 4 (*r* = .60) while having a significantly strong correlation at Time 3 (*r* = .89, *p* = .003). Additionally, the PC group demonstrated a significant positive correlation between omission errors and hit RT (*r* = .68, *p* = 04) at Time 1, which changed to non-significant negative correlations at Times 3 and 4. Furthermore, for the PC group, the omission errors were positively correlated with RTV at all time points, reaching statistical significance at Times 3 and 4 (*r* = .86, *p* = .006, and *r* = . 88, *p* = .003, respectively). Similarly, the correlations between commission errors and RTV were positive at all time points, with statistical significance observed at Times 2 and 4 (*r* = .77, *p* = .015, and *r* = .75, *p* = .018, respectively). Notably, the commission errors and hit RT correlated negatively for both groups at all time points of assessment, reaching statistical significance at Time 1 (*r* = −.77, *p* = .014) for the PC group and at Times 2 and 3 (*r* = −.98, *p* = .001, and *r* = −.92, *p* = .010, respectively) for the CP group.

Exploring performance relationships between ANT and Go/NoGo tasks revealed group-specific omission and commission error patterns. Hence, omission errors for the CP group mainly correlated positively with the alerting and orienting scores but negatively with the executive score. Conversely, the PC group consistently showed negative associations between omission errors and alerting score, with primarily positive trends related to the executive score. Regarding commission errors, both groups exhibited mainly negative correlations with executive score. Concerning hit RT, the CP group transitioned from a negative correlation with the alerting score at Times 1 and 2 to a positive correlation at Times 3 and 4. In contrast, the PC group shifted from a positive correlation at Times 1 and 2 to a negative correlation at Times 3 and 4.

The associations of working memory with the other variables also revealed group-specific dynamics. For the PC group, working memory correlated negatively with omission errors at all time points, ranging from moderate to strong correlations and reaching statistical significance at Times 2, 3, and 4 (*r* = −.87, *p* = .002, *r* = −.88, *p* = .004, and *r* = −.96, *p* < .000, respectively). Similarly, negative correlations were observed between working memory and commission errors at all time points, for the PC group, with a statistically significant negative correlation at Time 3 (*r* = −.73, *p* = .040). In contrast, the CP group exhibited non-significant positive correlations between working memory and commission errors at the four time points of assessment. Regarding the correlations between working memory and RTV, the PC group demonstrated negative correlations at all time points of assessment, with statistically significant correlations observed at Times 1, 3, and 4 (*r* = −.82, *p* = .007, *r* = −.72, *p* = .044, and *r* = −.81, *p* = .014, respectively).

In the analysis of the associations related to children’s performance in the Tower task, the PC group showed negative correlations with omission errors at all time points of assessment, ranging from weak to strong correlations and reaching significance at Times 3 and 4 (*r* = −.76, *p* = .028, and *r* = −.81, *p* = .015, respectively). Similarly, the correlations with commission errors were negative at all time points. In contrast, the CP group exhibited positive correlations with commission errors at all four time points of assessment. Additionally, the CP group exhibited negative correlations with hit RT at all time points. Conversely, the PC group demonstrated mixed positive and negative correlations with hit RT at each time point but showed negative correlations with RTV, reaching statistical significance at Times 1 and 4 (*r* = −.76, *p* = .017, and *r* = −.79, *p* = .019, respectively). Moreover, the Tower task correlated positively with working memory for both groups, with moderate correlations for the CP group and a range of weak to strong correlations for the PC group. Statistically significant negative correlations were observed at Times 3 and 4 (*r* = .93, *p* = .001, and *r* = .82, *p* = .013, respectively). However, the patterns differed in the relationship with processing speed, with a shift in direction from positive to negative correlations for the CP group and negative to positive correlations for the PC group.

Notable patterns emerged when examining the interplay between the various psychosocial domains. Both groups consistently exhibited positive correlations between social, school, and emotional competencies, underlying their interconnectedness. Specifically, the PC group exhibited moderate to strong correlations between social and school competencies, with a significant positive correlation at Time 4 (*r* = .86, *p* = .006). The CP group exhibited statistically significant strong positive correlations between social and school competencies at Times 1, 2, and 3 (*r* = .91, *p* = .004, *r* = .91, *p* = .013, and *r* = .92, *p* = .009, respectively). Additionally, both groups demonstrated statistically significant strong positive correlations between social and emotional competencies at all time points of assessment. Furthermore, both groups exhibited moderate to strong positive correlations between school and emotional competencies, with the CP group reaching statistical significance at Times 1 and 3 and the PC group at Times 2, 3, and 4. Similarly, self-perception formed positive correlations with social, school, and emotional competencies for both groups at all four-time points of assessment. In contrast, the CP group showcased negative correlations between behavioral problems and social, school, and emotional competencies and self-perception at all time points of assessment. The PC group exhibited negative correlations between behavioral problems and social and emotional competencies but mixed correlation patterns with school competence and self-perception.

#### Marginal models.

The subsequent step involved assessing the effects of the intervention (time), group (“PC” and “CP”), and age on the dependent variables (three scores from the ANT task, four scores from the Go/NoGo task, WMI, PSI, the total score of the Tower task, and five scores from Psychosocial Adjustment Test). Hence, a diverse set of marginal models was initially fitted (due to the potentially correlated errors) according to the assumed structure of the covariance matrix of errors. Then, the selection of the most appropriate model was determined using the AIC and BIC criteria [118–120], utilizing the “gls” function from the “nlme” package in R-project, maximizing the restricted log-likelihood function with the generalized least squares method [121]. One interaction term was considered among the exploratory variables: between time and group.

However, following a backward selection procedure, the interaction term was excluded from all models, except from one, while “age” was excluded from all models because its contribution was not significant in any model. Thus, for each model, a decision was made to retain only time and group as exploratory variables. The interaction was kept for the model with “school competence” as a dependent variable since its contribution was significant for that specific model. No conclusive evidence indicated a violation of model assumptions, encompassing linearity, normality of residuals, and independence between residuals and exploratory variables. Rigorous checks of these assumptions have been conducted based on the properties of the normalized residuals.

The estimated marginal models, presented in Table 2, indicate the effects of time and group on the dependent variables and the effect of the interaction between group and time on one of the variables (school competence). As we predicted (Hypotheses 1a, 1b, 1f, 2, and 5), the regression coefficients of Time suggest a decrease in the estimations for the executive score, omission errors, commission errors, hit RT, and behavioral problems over time (keeping the group fixed). Specifically, on average, there is a reduction of 27.20 milliseconds in the executive score (*β*_*2*_ = −27.20, SE = 4.45, *p* < .001) and 16.72 milliseconds in hit RT (*β*_*2*_ = −16.72, SE = 5.33, *p* = .002) as time passes. Additionally, there is, on average, a decrease of 2.00 units in omission errors (*β*_*2*_ = −2.00, SE = 0.65, *p* = .003), 3.44 units in commission errors (*β*_*2*_ = −3.44, SE = 1.52, *p* = .028), and 2.28 units in behavioral problems (*β*_*2*_ = −2.28, SE = 0.40, *p* < .001) from one time-point to another. This decline suggests improved attentional and executive functions and decreased behavioral problems over the intervention period.

**Table 2:** Estimation of marginal models (SE in parentheses), for each of the children’s dependent variables (using generalized least squares estimation method).

	Parameters (β)				
Dependent variable	Intercept	Group (PC: 1, CP: 0)	Time (2, 1 –4)	Group * Time	Correlation Structure
**Alerting Score**	50.04 (18.67)	−1.48 (12.82)	−7.38 (5.77)	--	Exponential variance
**Orienting Score**	53.85 (20.21)	−0.63 (19.20)	−7.99 (5.98)	--	Compound symmetry
**Executive Score**	142.59 (16.27)	21.53 (16.58)	−27.20 (4.45)***	--	Compound symmetry
**Omission Errors**	11.00 (3.38)	6.03 (2.79)*	−2.00 (0.65)**	--	Exponential variance
**Commission Errors**	41.60 (6.45)	1.89 (7.20)	−3.44 (1.52)*	--	Autoregressive, AR (1)
**Hit Reaction Time** **(Hit RT)**	456.78 (20.95)	20.38 (15.48)	−16.72 (5.33)**	--	Exponential variance
**Reaction Time Variability (RTV)**	0.29 (0.02)	0.03 (0.02)	−0.00 (0.00)	--	Compound symmetry
**Working Memory (WMI)**	14.92 (1.56)	0.75 (1.84)	1.07 (0.31)**	--	Autoregressive, AR (1)
**Processing Speed (PSI)**	13.87 (1.04)	3.18 (1.07)**	1.66 (0.28)***	--	Compound symmetry
**Tower Task** **Total Score**	12.59 (0.58)	−0.51 (0.66)	0.69 (0.13)***	--	Compound symmetry
**Social Competence**	47.43 (5.64)	−0.84 (7.09)	1.50 (0.80)	--	Compound symmetry
**School Competence**	38.22 (2.36)	−5.12 (3.12)	−0.48 (0.72)	2.49 (0.94)*	Compound symmetry
**Emotional Competence**	38.94 (4.90)	0.26 (5.62)	1.92 (1.08)	--	Autoregressive, AR (1)
**Self-Perception**	33.81 (1.77)	2.80 (1.99)	0.68 (0.41)	--	Compound symmetry
**Behavioral Problems**	51.59 (2.12)	10.85 (2.54)***	−2.28 (0.40)***	--	Compound symmetry

*p < .05. **p < .01. and ***p < .001.

In contrast, as time passes (keeping the group fixed), there is a consistent increase in the indices of working memory (*β*_*2*_ = 1.07, SE = 0.31, *p* = .001), processing speed (*β*_*2*_ = 1.66, SE = 0.28, *p* < .001), and the total score of the Tower task (*β*_*2*_ = 0.69, SE = 0.13, *p* < .001), as hypothesized (Hypotheses 3a, 3b, and 3c). This upward trajectory indicates positive changes in these cognitive domains throughout the intervention, suggesting enhancements in children’s working memory, cognitive processing speed, and planning and programming skills.

Furthermore, the impact of group assignments is noteworthy. Thus, there were between-subjects differences in omission errors, processing speed, and behavioral problems. Specifically, the estimated values for the children of the PC group were significantly higher in these domains than those of the CP group by 6.03 units, 3.18 units, and 10.85 units on average (and keeping time fixed). This finding indicates that children in the PC group tend to exhibit higher levels of omission errors (*β*_*1*_ = 6.03, SE = 2.79, *p* = .03), better processing speed (*β*_*1*_ = 3.18, SE = 1.07, *p* = .004), and more behavioral problems (*β*_*1*_= 10.85, SE = 2.54, *p* < .001) compared to their counterparts in the CP group.

The sole significant contribution of the effect of the interaction between time and group is observed on school competence (*β*_*Group X Time*_ = 2.49, SE = 0.94, *p* = .010). Hence, the change in school competence scores over time differs significantly between the PC and CP groups. Specifically, the PC group experienced a more pronounced improvement in school competence over the assessment periods than the CP group. Additionally, despite the expectation that the CP group would show better cognitive outcomes at Time 2 due to earlier exposure to iVR-based cognitive training (Hypothesis 7), the interaction between time and group was not significant for any cognitive outcomes, indicating no differential pattern of change between CP and PC groups across time in these measures.

In summary, the results align with some of our expectations and indicate overall positive trends for children, with improvements in attention, executive functions, and reduced behavioral problems compared to their initial (baseline) assessments. Additionally, time and group assignment influence specific domains, such as school competence, with PC group showing greater gains over time. In contrast, the results did not support our hypotheses regarding improvements in alerting and orienting scores, social, emotional, and school competencies, and self-perception.

### Feasibility and acceptability outcomes

The ECT-F and EVR-F questionnaires, administered in the last session of the child training program, offered valuable insights into its utility, feasibility, and acceptability. Table 3 presents descriptive statistics for the scores extracted from the two questionnaires and their correlation coefficients. Notably, there were some significant correlations between these variables and the study’s dependent variables, as described below.

**Table 3:** Means, standard deviations, and Pearson correlation coefficients of the scores extracted from the Evaluation of Child Training Final (ECT-F) and Evaluation of VR Experience Final (EVR-F) Questionnaires.

				Evaluation of Child Training – Final (ECT-F)						Evaluation of VR Experience – Final (EVR-F)				
				Content/ Structure			Utility/ Feasibility			VR Cybersickness Symptoms (SSQ)				
Variable	M	SD	1	2	3	4	5	6	7	8	9	10	11	12
**1. Sessions attended**	14.06	4.07												
**2. Relationship with Therapist**	17.79	0.57	−.23											
**3. Benefits from the Program**	22.50	2.50	−.38	.61*										
**4. Total Satisfaction**	40.29	2.89	−.37	.73**	.99**									
**5. Program Usability**	13.07	5.67	−.44	.08	.35	.32								
**6. Program Acceptance**	18.86	1.83	−.20	.33	.43	.44	.02							
**7. VR_Total Time**	350.68	115.48	.91**	−.24	−.25	−.26	−.35	−.11						
**8. SSQ Nausea**	0.14	0.53	.17	.11	.17	.17	−.16	.18	.20					
**9. SSQ Occulomotor**	0.07	0.27	.17	.11	−.06	−.03	.05	−.61*	−.10	−.08				
**10. SSQ Disorientation**	0.57	1.22	−.29	−.03	−.10	−.09	.13	.04	−.24	.34	−.14			
**11. VR Sense of Presence**	11.43	4.75	−.36	.51	.61*	.63*	.39	.43	−.11	.34	−.15	.30		
**12. VR Enjoyment/ Engagement**	7.36	1.98	.07	.34	.38	.40	.01	.44	.04	.38	−.49	−.28	.50	
**13. VR Usability**	14.43	2.06	−.09	.08	.22	.21	.01	.91**	.16	.22	−.62*	−.10	.36	.50

*p < .05. **p < .01.

As mentioned earlier, 14 children completed the entire child training program. Participants, on average, attended 14.06 sessions (*SD* = 4.07), indicating consistent engagement with the program. Overall, children expressed high satisfaction with the program (*M* = 40.29, *SD* = 2.89). Specifically, the mean for the relationship with the therapist was notably high (*M* = 17.79, *SD* = .57), while all children reported having a good time and feeling cared for by the facilitators, indicating positive interactions during the sessions. Additionally, children reported substantial benefits from the program (*M* = 22.50, *SD* = 2.50) since all of them reported feeling better after attending the sessions (78.6% very much, 21.4% moderate/much), learning valuable things that will help them in their lives (78.6% very much, 21.4% moderate/much), and positive impacts on family relationships (71.4% very much, 21.4% much, 7.1% somewhat) and self-confidence. Usability (*M* = 13.07, *SD* = 5.67) and acceptance (*M* = 18.86, *SD* = 1.83) of the program were generally positive, with a strong agreement that the program is beneficial for children (92.9% strongly agreed, 7.1% agreed) reflected in their interest in participating again or in a similar program.

In assessing the VR experience, participants spent an average of 350.68 minutes engaging with the equipment and implementing the tasks in the virtual classroom. The SSQ indicated minimal cybersickness symptoms, with children enjoying (*M* = 7.36, *SD* = 1.98) and finding the VR equipment and tasks feasible and easy to use (*M* = 14.43, *SD* = 2.06). More precisely, all children enjoyed using the VR equipment and the tasks (85.7% yes, 14.3% maybe). The majority expressed interest in using it again (78.6% yes, 7.1% maybe) and showing the tasks to others (friends, siblings), while only two children would not want to use it again or show the tasks to others. Also, although some participants (two children) expressed their desire for more sessions, most thought the number of sessions was appropriate (85.7%).

Correlation analysis between the scores from the ECT-F and EVR-F questionnaires revealed non-significant negative correlations between the number of sessions and various variables, including the relationship with the therapist, benefits from the program, total satisfaction, program usability, and acceptance, as well as disorientation and the sense of presence in the virtual classroom. However, as anticipated, the relationship with the therapist showed a significant moderate positive correlation with the benefits from the program (*r* = .61, *p* = .02), while the total satisfaction score exhibited a strong positive correlation with both scores (*r* = .73 and *r* = .99 respectively). Positive but not statistically significant correlations were observed between the program’s usability and acceptance, the relationship with the therapist, and perceived program benefits. Notably, oculomotor discomfort correlated significantly and negatively with program acceptance (*r* = −.61, *p* = .02), suggesting that participants reporting higher levels of oculomotor discomfort are likely to express lower acceptance toward the program. Additionally, the sense of presence in the virtual environment exhibited significant moderate positive correlations with the perceived benefits of the program (*r* = .61, *p =* .02) and the level of children’s satisfaction from the program (*r* = .63, *p* = .01), as well as non-significant moderate positive correlations with sessions attended, program usability and acceptance. The perceived usability of VR technology (equipment and tasks) had a strong positive correlation with the acceptance of the program (*r* = .91, *p* < .001) and a negative correlation with disorientation (*r* = −.62, *p* = .01). Therefore, when children feel that iVR technology is easy to use and comfortable, they are more favorable toward the program. Conversely, if they experience higher levels of disorientation after using VR, they will express lower levels of usability of iVR technology.

Some intriguing findings emerged when examining the correlations between the variables of the ECT-F and EVR-F questionnaires and the study’s dependent variables (see S2 Table). Notably, the relationship with the therapist benefits from the program, and the total satisfaction score exhibited moderate and strong positive correlations with social competence, school competence, and emotional competence at all time points, with statistically significant correlations noted. Additionally, self-perception displayed weak and moderate positive correlations with these variables, reaching significance at Times 3 and 4. In contrast, behavioral problems showed non-significant negative correlations with these three variables at all time points. Moreover, the sense of presence in the virtual environment correlated negatively with the Tower task at all time points but positively with social, school, and emotional competence and self-perception (statistically significant correlations noted at Times 3 and 4). Also, a moderate positive correlation was observed between VR engagement/enjoyment and emotional competence, with statistically significant correlations at Times 1 and 2. Furthermore, omission errors correlated significantly positively with disorientation after using iVR technology at all time points. Finally, the hit RT showed an increasing positive correlation with VR total time, reaching significance by Times 3 and 4.

In addition to quantitative findings, participants’ qualitative informal feedback during and after the sessions offered insight into their subjective experiences with the child training program. While not part of a formal qualitative analysis, these reflections help to highlight the positive experiences and perceived utility of the program, since they illustrate children’s enthusiasm for iVR technology, the virtual classroom, and the CBT-based activities (e.g., board games, stories, etc.). Importantly, children acknowledged that acquiring skills and techniques would be beneficial for the future. Several children spontaneously shared comments such as: *“I had a perfect time”*, *“I learned many things during the program which will help me”*, *“I would love to have more sessions”*, *“In the virtual classroom, I liked that we had a new goal every time and I was trying to surpass my record”*. Such statements suggest that the program was engaging and perceived as valuable from the child’s perspective.

## Discussion

The current study aimed to develop and assess the potential effectiveness of a multimodal intervention program –the Child ViReal Support Program– that integrates parent training, child training, and iVR-based cognitive training. This innovative combination was designed to enhance attentional skills and executive functions in children with attention deficits while simultaneously supporting parents in managing their children’s challenges. Unlike previous interventions that focus on a single modality, this program brings together behavioral, emotional and neurocognitive components in a coordinated structure, aiming to address the child’s difficulties in a more comprehensive and synergistic way.

Accordingly, this study examined the program’s potential impact on a broad range of cognitive outcomes – including sustained and focused attention, attentional control, inhibition control, working memory, processing speed, and planning/programming abilities – as well as on psychosocial outcomes, such as social, school, and emotional competencies, self-perception skills, and behavioral problems. In doing so, the study offers a preliminary but valuable contribution to the growing evidence base supporting integrated, technology-enhanced interventions for children with attention deficits and their families.

### Cognitive outcomes

Our study revealed significant improvements in various *cognitive domains* over the intervention period, as evidenced in sustained attention, attentional and inhibitory control, working memory, processing speed, and planning abilities, which were sustained four months after the end of the program. The results indicate that the participants demonstrated significantly lower omission and commission errors, hit RT, and executive score at post-test and follow-up, indicating a higher attentional and inhibitory control and enhanced sustained attention by the end of the program. These findings align with previous research showing that cognitive training, either computer-based or iVR-based, can produce positive effects on sustained attention, vigilance, inhibitory control, and cognitive flexibility, as measured by decreased omission and commission errors and lower hit RTs [43,51–53,122,123]. Additionally, these results are consistent with the parent reports regarding their children’s reduced inattention and impulsivity/hyperactivity symptoms after the intervention and at the follow-up assessment outlined in a previous study by the authors [71].

However, when the sample was split into two groups (PC and CP), the statistical significance was maintained only for the executive score and omission errors for both groups.

Additionally, there were no significant differences in the RTV, a finding that is consistent with previous research in which no differences were observed after cognitive training [124]. This outcome could be interpreted by the way we computed the RTV in this research or by the small sample size, which could limit the study’s statistical power to detect subtle changes in the RTV over time. Similarly, no significant changes were observed in alerting or orienting scores. This may reflect the fact that these specific attention networks may be less responsive to short-term training, particularly in children with attention deficits, as alerting and orienting processes are thought to mature earlier or differently and be more stable and less amenable to change than executive control functions [125,126]. Additionally, the small sample size and resulting limited statistical power may have reduced the ability to detect subtle changes in these measures.

Furthermore, significant increases in working memory and processing speed were observed in the whole sample, suggesting gains in cognitive processing efficiency and resource allocation. Notably, when the sample was split into two groups, the increase in working memory was evident and significant for both groups. However, only the PC group showed significant improvements in processing speed, particularly after participating in the child training program. In contrast, although the children in the CP group showed increased processing speed in the assessments following their initial assessment, they did not reach statistical significance. This difference between the two groups could probably be explained by the smaller sample size of the CP group since two participants discontinued during the study, which could affect statistical power.

Moreover, both groups improved performance on the Tower task, reflecting enhanced planning and problem-solving abilities. Specifically, the PC group exhibited better performance after participating in the child training program, with the results remaining significantly higher compared to their initial assessment four months after the end of the intervention. The children in the CP group performed better at Time 2, namely after their child training ended. However, the increased results at the Time 3 and 4 assessments did not differ significantly from their initial assessments. These findings are consistent with previous studies showing that child-oriented cognitive training has significant effects on children’s executive functions and planning abilities [127,128] while iVR-based cognitive training has positive effects on working memory [54,55] and processing speed [129].

### Psychosocial outcomes

Regarding *psychosocial outcomes*, children reported significantly lower behavioral problems over the intervention period compared to their initial reports, and this decrease was still present at the follow-up assessment. The decrease aligns with the parents’ reports, which observed decreased inattention and impulsivity/hyperactivity symptoms in their children [71] along with the results from the computerized cognitive tasks (i.e., fewer omission and commission errors). At the same time, these results are consistent with previous research suggesting that iVR-based cognitive training [56,57] and CBT-based child training [30,64] can improve children’s symptoms of impulsivity/hyperactivity and behavioral problems.

However, contrary to our expectations, children did not report significant improvements in social competence and self-perception over the intervention period. These findings may reflect the fact that social functioning and self-perception tend to influence – and be shaped by – ongoing interpersonal experiences and feedback, and thus often require longer-term interventions and reinforcement in naturalistic settings before measurable changes emerge [130]. Additionally, the self-report nature of these measures may have limited sensitivity to detect subtle changes. Regarding emotional competence, even though children in both groups reported enhanced abilities, only the PC group reached statistical significance. This difference may be related to the sequencing of the intervention, as receiving parent training first may have created a more supportive home environment that reinforced emotional regulation skills during subsequent child training sessions [131].

### The impact of group assignment on intervention outcomes

Furthermore, the impact of group assignments on intervention outcomes was evident, particularly concerning omission errors, processing speed, and behavioral problems. Specifically, the children of the PC group demonstrated higher levels of omission errors and higher processing speed and reported more behavioral problems than those of the CP group. The impact of the group on the processing speed and behavioral problems might be explained by the difference observed in the initial assessment (Time 1) between the two groups in these two variables. Still, the impact of the group on the omission errors could be explained by the difference in the decrease observed during the intervention period, since the omission errors at the initial assessment are similar between the two groups. Moreover, the difference in the sample sizes of the two groups at the end of the intervention might be another explanation for these findings.

Also, the interaction between time and group assignment significantly influenced school competence, indicating that the timing of intervention components may play a crucial role in shaping specific outcomes. This finding highlights the importance of considering the sequencing of parent and child training components and individual differences to maximize the program’s effectiveness, particularly in promoting academic functioning and school success. In contrast, sequencing did not appear to influence cognitive outcomes, as no differential effects emerged between CP group and PC group after Time 2 assessment, suggesting that the timing of iVR-based cognitive training exposure did not differentially influence these measures. This may be due in part to the limited sample size, which reduces statistical power and may have masked subtle sequencing effects. Future research with larger samples is needed to explore whether the sequencing of intervention elements plays a more decisive role in shaping cognitive gains when statistical power is sufficient to detect more nuanced group differences.

### Limitations

Overall, our research provides encouraging preliminary evidence supporting the potential effectiveness of the *Child ViReal Support Program* as a promising intervention for children with attention deficits and their parents. However, it is essential to note the study’s limitations, such as the small sample size and potential biases associated with self-report measures. The small sample size evoked challenges, mainly when analyzing the two groups separately, limiting the ability to detect significant differences between the two elements of the intervention program (child training and parent training). Furthermore, the reduced number of participants who completed the Time 4 follow-up further limited statistical power and may affect the stability of the long-term results, underscoring the need for cautious interpretation. Nonetheless, it is notable that significant changes emerged post-intervention, even within this relatively small sample compared to initial assessments for children and parents.

Additionally, the absence of a control group restricts the ability to attribute observed changes solely to the intervention, as improvements may reflect maturation or other non-specific factors. It is also important to note that effect sizes varied considerably across domains. For example, improvements in executive score were associated with a large effect size, whereas other outcomes, such as working memory and emotional competence, showed smaller effects. These differences in magnitude should be interpreted with caution in terms of clinical significance. While the large effects suggest potential for clinically meaningful change, smaller effects may relate to skills that develop more gradually or require longer exposure to training.

These constraints underscore the importance of viewing this work as a pilot study. As such, the findings are best understood as hypothesis-generating and intended to inform future research. Follow-up studies with larger and more diverse samples, as well as randomized controlled designs, are needed to replicate and extend these results, examine sequencing effects more robustly, explore the mechanisms underlying intervention effects and the robustness and clinical relevance of both large and small effect sizes. Hence, based on the experience gained in this pilot study, future studies could benefit from extending the recruitment network to increase sample diversity, lengthening follow-up periods to assess maintenance of effects, and including a control group (e.g., a pure CBT or iVR group) to strengthen causal inference. Additionally, refining certain measures to reduce reliance on self-report and ensuring balanced group sizes may further enhance statistical power and interpretability. Finally, testing the intervention in different settings (e.g., schools, community clinics) could help determine its scalability and generalizability.

## Conclusion

In conclusion, the *Child ViReal Support Program* demonstrates preliminary positive results for children with attention deficits and their parents by enhancing executive functions and reducing behavioral problems, inattention, and impulsivity/hyperactivity symptoms. Additionally, the program benefits parents by improving their practices, increasing parental self-efficacy, and reducing their stress. The combined quantitative and qualitative data affirm the overall acceptability of the program and iVR-based cognitive training. These findings, although preliminary and influenced by the small sample size, offer meaningful insights into the potential cognitive and behavioral benefits of the combined intervention. Additionally, they highlight the importance of comprehensive, multimodal intervention approaches employing innovative technologies like iVR technology to address the needs of families dealing with attention deficits/ADHD. The methodological insights gained from this pilot also provide concrete recommendations for optimizing the design of future studies. The implications of this study extend to professionals such as school psychologists and clinicians, emphasizing the importance of evidence-based interventions in promoting positive outcomes and improving the quality of life and well-being of children and families affected by attention deficits.

## Supporting information

S1 Appendix Sessions of the Child Training Program. (DOCX)

S2 Appendix iVR-based cognitive tasks. (DOCX)

S1 Table Pearson correlation coefficients of the children’s variables for each time and group separately. (DOCX)

S2 Table Pearson correlation coefficients (Child Training assessment variables and dependent variables), for each time separately. (DOCX)

S1 Checklist CONSORT Checklist. (DOC)

S2 File Trial Protocol_Greek. (DOCX)

S3 File Trial Protocol_Eng. (DOCX)
