# Correction: Child training in the Child ViReal Support Program: Combining iVR-based cognitive training and CBT techniques in a pilot study

**DOI:** 10.1371/journal.pone.0345666

**Published:** 2026-03-24

**Authors:** Iouliani Pachiti, Fotios S. Milienos, Timothy C. Papadopoulos, Panagiota Dimitropoulou

The following information is missing from the Acknowledgments statement: The 3D model of the virtual classroom that was modified for the purpose of the study, was provided by Andria Shimi of the University of Cyprus.
