# Supplementary material for: Child training in the Child ViReal Support Program: Combining iVR-based cognitive training and CBT techniques in a pilot study
Source: PLoS One. 2026 Feb 27;21(2):e0343364. doi: 10.1371/journal.pone.0343364 (PMC12948055; doi:10.1371/journal.pone.0343364)
Supplement: S1 Appendix — (DOCX) [file pone.0343364.s001.docx]

**S1 Appendix. Sessions of the Child Training Program**

|  | Topic | Session components |
| --- | --- | --- |
| 1 | Thoughts-Emotions-Actions | - How thoughts, emotions, and actions interact and impact one another |
| 2 | A mind full of thoughts! | - Automatic negative thoughts - Transformation of negative thoughts into positive ones |
| 3 | I am learning to concentrate | - Attention/ External and internal distractors - Strategies for managing distractions |
| 4 | My emotions! | - Identification and categorization of pleasant and unpleasant emotions |
| 5 | I express my emotions! | - Effective expressions of pleasant and unpleasant emotions (e.g., facial expressions, body posture) |
| 6 | I detect and control my anger! | - Identification of situations and events that cause anger - Recognition of ‘early warning’ signs of anger in the body - Anger management techniques |
| 7 | I detect and control my stress! | - Identification of situations and events that cause stress - Recognition of stress signs in the body - Stress management techniques |
| 8 | I am learning to control myself! | - Impulse reactions might cause negative consequences - “Stop-Think-Act” technique |
| 9 | Social skills and Friendly relationships | - The creation and maintenance of friendly relationships are based on social skills - Training on social skills (e.g., polite interactions with others, etc.) |
| 10 | I am learning how to solve problems and conflicts! | - Conflicts might arise during people’s interactions - Conflict resolution strategies |
| 11 | I am learning how to analyze problems! (Problem-solving strategies I) | - Identification of everyday problems - Analysis of problems by paying attention to details, asking appropriate questions, and collecting necessary information |
| 12 | I am learning how to solve problems! (Problem-solving strategies II) | - Next steps of the problem-solving process - Find alternative solutions. / Consider the pros and cons of each solution before choosing the appropriate solution to solve a problem. |
| 13 | I am learning to set goals and achieve them! | - The importance of setting goals for the future - Strategies for goal formulation, time management, and division of a goal into achievable steps |
| 14 | I am learning to observe, evaluate, and congratulate myself! | - Self-guidance, self-observation, and self-reinforcement techniques |
| 15 | I love myself! | - Self-esteem and self-confidence/ Finding positive elements and characteristics of the child’s self - Self-reinforcement techniques |
| 16 | You made it! Congratulations! | - Wrap-up and review of the sessions and content covered through the child training program |
