# Supplementary material for: Child training in the Child ViReal Support Program: Combining iVR-based cognitive training and CBT techniques in a pilot study
Source: PLoS One. 2026 Feb 27;21(2):e0343364. doi: 10.1371/journal.pone.0343364 (PMC12948055; doi:10.1371/journal.pone.0343364)
Supplement: S2 Appendix — (DOCX) [file pone.0343364.s002.docx]

**S2 Appendix. iVR-based cognitive tasks**

**Card Sorting Task**

**
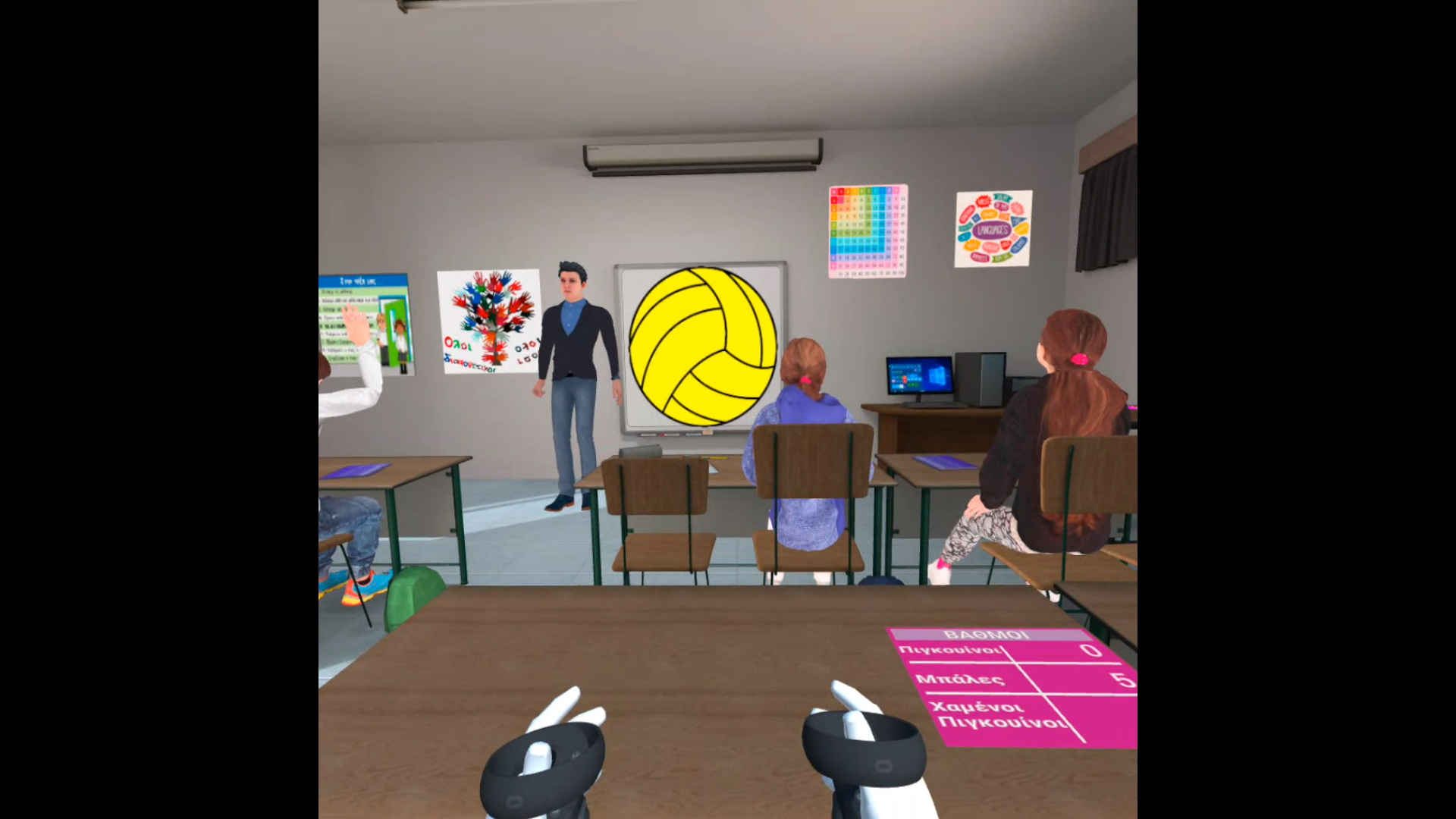

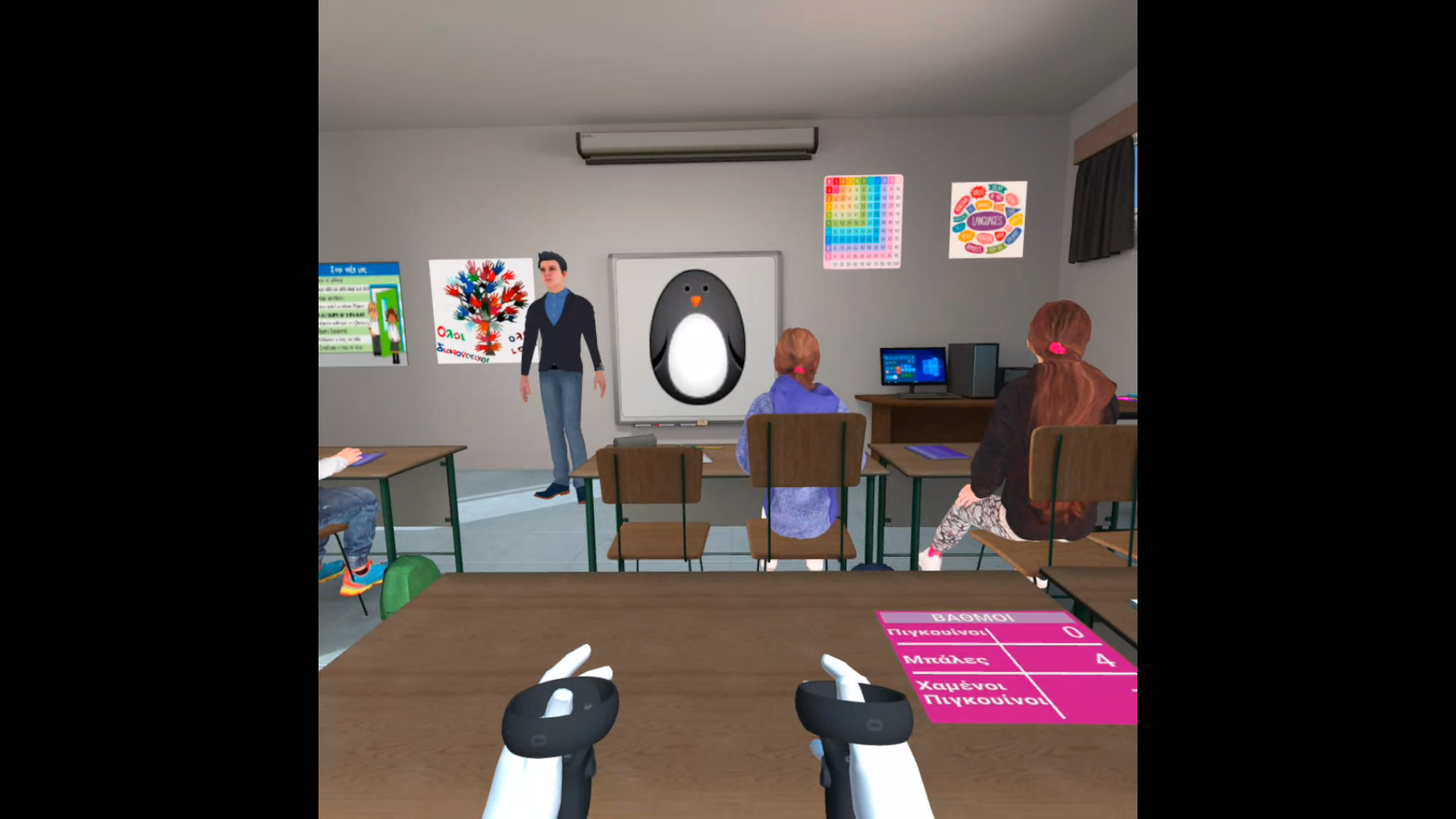

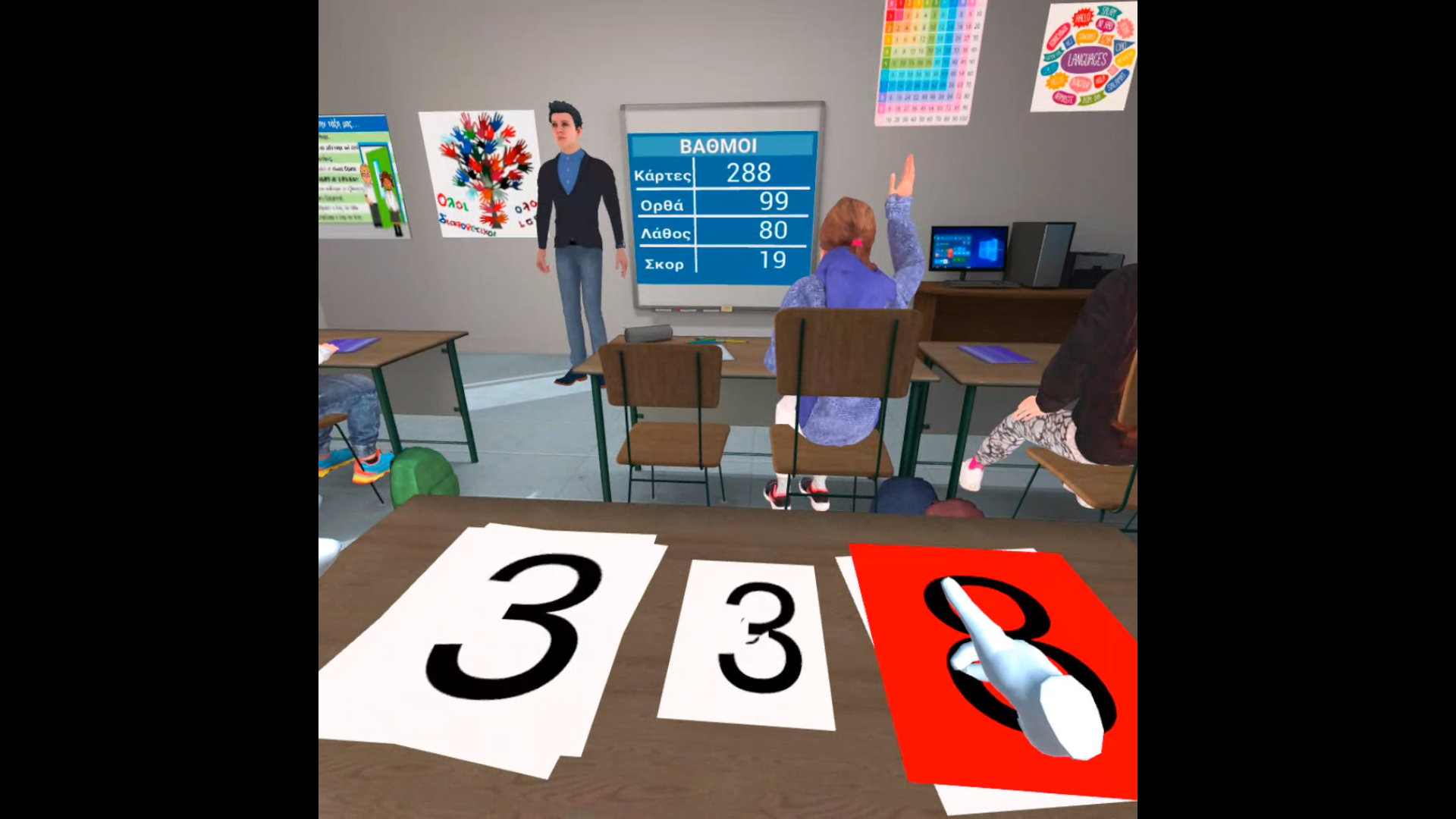

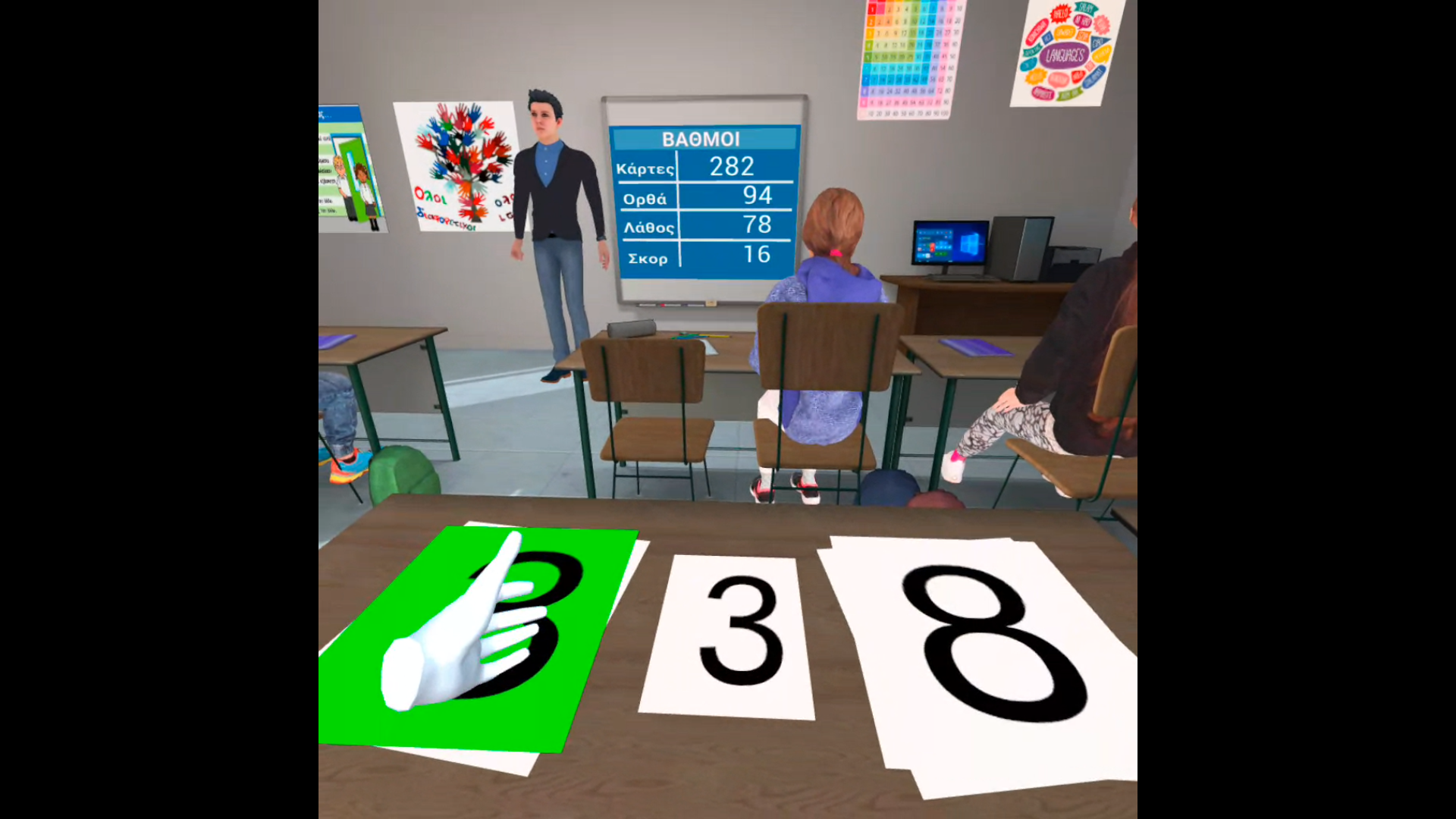
**

**CPT Task**

| **iVR-based cognitive tasks parameters** | |
| --- | --- |
| **Hardware setup** | |
| Head-mounted display (HMD) | Oculus Quest 1 (Meta Platforms and Technologies, California, US) |
| Resolution and refresh rate | High-resolution OLED display  1400 X 1600 pixels per eye  72Hz refresh rate |
| Field of view | 93^o^ horizontally X 93^o^ vertically |
| Controllers | Two Oculus Touch controllers |
| Audio | Over-ear headphones for immersive sound |
| Tracking | 6 degrees of freedom (DoF) positional tracking  4 built-in cameras |
| Physical space setup | Seated experience (as in a classroom setting) |
| Response mode | Pressing the trigger button on the controllers |
| **Software** | |
| Platform | Unity3D game engine (version 2019.4; Unity Technologies, San Francisco, CA, US) |
| Programming language | C# |
| Custom environment | Virtual classroom adapted from Rizzo et al.’s virtual classroom paradigm |
| Task type | Focused and sustained attention training |
| Avatars | Teacher avatar at the front, student avatars in three rows of desks |
| Visual details | Pictures on walls, whiteboard in front, door, window with schoolyard view |
| Distractors | Auditory (e.g., pencil dropping, footsteps, ambient classroom sounds)  Visual (e.g., classmates raising hands, teacher movement past open door etc.)  Mixed (combined audio and visual cues)  Distractor frequency and intensity increased with task progression. |
| **Tasks** | |
| Card Sorting task | Cards appear on a virtual desk; child sorts the cards based on specific criteria (e.g., number). Card representation speed increases with level progression. |
| Continuous Performance task (CPT) | Stimuli presented on whiteboard; respond to Go stimuli (targets; e.g., fruits) via controller button press; inhibit response to NoGo stimuli (non-targets; e.g., monkey). Target presentation intervals increase over time. |
| **Task Structure** | |
| Levels | Three progressive levels per task, each with two difficulty settings (Easy / Difficult) |
| Session allocation | One task per session, selected based on program guidelines and individualized progress of each participant |
| Session duration | 20 – 30 minutes (based on level and difficulty setting) |
| Number of sessions | 16 |
| Difficulty progression | Card Sorting task: Increased card appearance speed  CPT task: Longer intervals between target stimuli, requiring sustained attention |
| Feedback & Rewards | Performance-based progression to next difficulty level  Bonus game (e.g., bowling, tennis) from HMD library awarded if session goal met (e.g., minimum number of correct responses) |
| Familiarization session | First session included training exercises (demos) for both tasks and a bonus game to introduce HMD controls, interaction mechanics and iVR environment |

| **Sessions’ outline** | | | |
| --- | --- | --- | --- |
| **Session** | **Task** | **Description** | **Duration** |
|  | Familiarization session | First introduction to iVR equipment and the two tasks | 10 minutes |
|  | Card Sorting task | Level 1_Easy (1200 trials, 4 seconds interval) | 20 minutes |
|  | CPT task | Level 1_Easy (1200 trials, 240 max wrong)  80% targets = fruits/ vegetables,  20% non-targets = monkey | 20 minutes |
|  | Card Sorting task | Level 2_Easy (1500 trials, 3 seconds interval) | 25 minutes |
|  | CPT task | Level 2_Easy (1500 trials, 300 max wrong)  80% targets = fishes,  20% non-targets = shark | 25 minutes |
|  | Card Sorting task | Level 3_Easy (1500 trials, 2 seconds interval) | 25 minutes |
|  | CPT task | Level 3_Easy (1800 trials, 360 max wrong)  80% targets = awards/badges,  20% non-targets = alien | 30 minutes |
|  | Card Sorting task | Level 1_Difficult (1200 trials, 3 seconds interval) | 25 minutes |
|  | CPT task | Level 1_Difficult (1500 trials, 1200 max wrong)  20% targets = heart,  80% non-targets = viruses | 25 minutes |
|  | Card Sorting task | Level 2_Difficult (1350 trials, 3 seconds interval) | 30 minutes |
|  | CPT task | Level 2_Difficult (1800 trials, 1440 max wrong)  20% targets = pinguin,  80% non-targets = balls | 30 minutes |
|  | Card Sorting task | Level 3_Difficult (1500 trials, 2 seconds interval) | 30 minutes |
|  | CPT task | Level 3_Difficult (1800 trials, 1440 max wrong)  20% targets = present,  80% non-targets = monsters | 30 minutes |
|  | Card Sorting task | Level 3_Difficult (1500 trials, 2 seconds interval) | 30 minutes |
|  | CPT task | Level 3_Difficult (1800 trials, 1440 max wrong)  20% targets = present,  80% non-targets = monsters | 30 minutes |
|  | Card Sorting task **or**  CPT task | Level 3_Difficult  Level 3_Difficult | 30 minutes  30 minutes |
