# Supplementary material for: Child training in the Child ViReal Support Program: Combining iVR-based cognitive training and CBT techniques in a pilot study
Source: PLoS One. 2026 Feb 27;21(2):e0343364. doi: 10.1371/journal.pone.0343364 (PMC12948055; doi:10.1371/journal.pone.0343364)
Supplement: S1 Table — (DOCX) [file pone.0343364.s003.docx]

**S1 Table. Pearson correlation coefficients of the children’s variables for each time and group separately.**

| Variable | | Time | 1 | 2 | 3 | 4 | 5 | 6 | 7 | 8 | 9 | 10 | 11 | 12 | 13 | 14 | 15 |  |
| --- | --- | --- | --- | --- | --- | --- | --- | --- | --- | --- | --- | --- | --- | --- | --- | --- | --- | --- |
| 1. ANT  Alerting  Score | | 1 | ─ | -.08 | -.20 | -.17 | -.26 | .17 | -.63 | .47 | -.36 | .70* | -.12 | -.14 | .06 | -.16 | .23 |  |
|  |  | 2 | ─ | -.44 | -.25 | -.06 | -.27 | .29 | -.15 | .17 | .40 | -.37 | .59 | .70* | .85** | .32 | -.07 |  |
|  |  | 3 | ─ | -.06 | -.23 | -.04 | -.09 | -.71* | .38 | .26 | -.24 | .25 | -.40 | .03 | -.35 | .38 | .79* |  |
|  |  | 4 | ─ | . 13 | -.38 | -.28 | .34 | -.27 | -.20 | .38 | .71* | .15 | .04 | .36 | .38 | .34 | .18 |  |
| 2. ANT Orienting Score | | 1 | -.41 | ─ | -.24 | -.18 | .36 | -.42 | -.12 | -.03 | -.10 | .35 | -.07 | .34 | -.07 | -.23 | -.29 |  |
|  |  | 2 | -.46 | ─ | -.43 | .11 | .10 | .24 | -.21 | -.09 | .09 | .06 | -.25 | -.61 | -.50 | -.18 | -.21 |  |
|  |  | 3 | -.02 | ─ | .54 | -.22 | .08 | .25 | -.38 | .55 | .84** | .55 | .39 | .38 | .41 | .61 | -.03 |  |
|  |  | 4 | .55 | ─ | .59 | .60 | .64 | -.17 | .52 | -.60 | -.16 | -.30 | .25 | .22 | .39 | .07 | -.19 |  |
| 3. ANT Executive Score | | 1 | .42 | -.87* | ─ | .81** | -.24 | .62 | .32 | -.31 | -.02 | -.21 | .11 | .28 | -.26 | -.06 | .26 |  |
|  |  | 2 | .64 | -.40 | ─ | .51 | .35 | -.02 | .42 | -.39 | -.05 | -.02 | .17 | -.08 | -.06 | -.14 | -.16 |  |
|  |  | 3 | .55 | .13 | ─ | -.35 | -.30 | .27 | -.59 | .41 | .84** | .46 | .63 | .60 | .57 | .33 | .09 |  |
|  |  | 4 | .73 | .96** | ─ | .54 | -.04 | -.01 | .17 | -.65 | -.29 | -.21 | .13 | -.23 | .02 | .15 | .16 |  |
| 4. Go/NoGo Omission Errors | | 1 | .11 | .57 | -.34 | ─ | -.25 | .68* | .42 | -.60 | -.23 | -.22 | .05 | -.12 | -.43 | .14 | .30 |  |
|  |  | 2 | -.24 | .33 | -.35 | ─ | .58 | .39 | .32 | -.87** | .19 | -.54 | .23 | -.37 | -.26 | -.01 | -.64 |  |
|  |  | 3 | .46 | -.25 | -.13 | ─ | .89** | -.40 | .86* | -.88** | -.14 | -.76* | .04 | .27 | .12 | .28 | -.04 |  |
|  |  | 4 | .62 | .62 | .61 | ─ | .60 | -.45 | .88** | -.96** | -.63 | -.81* | .37 | .28 | .15 | -.12 | -.26 |  |
| 5. Go/NoGo Commission Errors | | 1 | -.18 | .44 | -.55 | -.11 | ─ | -.77* | .58 | -.45 | .47 | -.18 | .19 | .25 | .37 | -.25 | -.76* |  |
|  |  | 2 | .33 | -.44 | -.34 | -.32 | ─ | -.39 | .77* | -.64 | -.52 | -.43 | .28 | -.00 | -.24 | .36 | -.35 |  |
|  |  | 3 | .07 | -.73 | -.44 | .07 | ─ | -.19 | .69 | -.73* | .06 | -.69 | .27 | .42 | .39 | .51 | -.13 |  |
|  |  | 4 | -.97** | -.36 | -.56 | -.60 | ─ | -.60 | .75* | -.41 | -.08 | -.42 | .21 | .53 | .32 | -.02 | -.15 |  |
| 6. Go/NoGo Hit Reaction Time (HitRT) | | 1 | -.09 | .20 | -.04 | .73 | -.67 | ─ | -.22 | -.05 | -.56 | .03 | -.10 | -.33 | -.52 | .05 | .51 |  |
|  |  | 2 | -.42 | .47 | .18 | .48 | -.98** | ─ | -.67* | -.12 | .69* | -.23 | .26 | -.32 | .15 | -.31 | -.21 |  |
|  |  | 3 | .09 | .55 | .46 | .12 | -.92** | ─ | -.64 | .17 | .19 | -.04 | .56 | -.15 | .42 | -.29 | -.47 |  |
|  |  | 4 | .70 | .62 | .74 | .50 | -.66 | ─ | -.53 | .30 | -.12 | .31 | .03 | -.16 | -.09 | .05 | -.47 |  |
| 7. Go/NoGo  Reaction Time Variability (RTV) | | 1 | .67 | .16 | -.10 | .65 | .13 | .21 | ─ | -.82** | .48 | -.76* | .12 | .07 | .07 | .13 | -.22 |  |
|  |  | 2 | .29 | .01 | -.38 | -.05 | .80 | -.79 | ─ | -.49 | -.54 | -.11 | .01 | .18 | -.16 | .15 | -.20 |  |
|  |  | 3 | .66 | -.53 | .19 | .38 | .43 | -.06 | ─ | -.72* | -.45 | -.62 | -.28 | -.03 | -.23 | .19 | .34 |  |
|  |  | 4 | -.43 | .37 | .23 | -.37 | .61 | .05 | ─ | -.81* | -.60 | -.79* | .33 | .38 | .22 | -.30 | -.40 |  |
| 8. Working Memory Index (WMI) | | 1 | -.15 | .09 | .11 | .32 | .32 | .01 | .10 | ─ | .07 | .47 | .16 | .33 | .39 | .15 | .30 |  |
|  |  | 2 | .54 | -.58 | .32 | -.11 | .48 | -.57 | .56 | ─ | .17 | .25 | .08 | .31 | .43 | .13 | .72* |  |
|  |  | 3 | -.61 | -.24 | -.80 | .29 | .28 | -.40 | -.46 | ─ | .38 | .93** | -.10 | -.13 | -.14 | .07 | .18 |  |
|  |  | 4 | -.41 | -.51 | -.50 | -.28 | .26 | .16 | .02 | ─ | .68 | .82* | -.41 | -.20 | -.19 | .13 | .33 |  |
| 9. Processing Speed Index (PSI) | | 1 | -.29 | .17 | -.04 | -.42 | .54 | -.66 | -.24 | .02 | ─ | -.53 | .65 | .67* | .85** | .52 | -.08 |  |
|  |  | 2 | -.55 | .50 | .00 | -.38 | -.55 | .48 | -.55 | -.71 | ─ | -.34 | .19 | -.18 | .23 | .04 | -.14 |  |
|  |  | 3 | -.31 | .38 | .26 | -.90* | -.31 | .25 | -.16 | -.54 | ─ | .46 | .58 | .69 | .63 | .61 | -.11 |  |
|  |  | 4 | .72 | .49 | .67 | .13 | -.66 | .87 | .11 | .00 | ─ | .48 | .06 | .23 | .39 | .65 | .46 |  |
| 10. Tower | | 1 | -.03 | -.27 | .54 | .00 | .14 | -.16 | .06 | .72 | .43 | ─ | -.15 | -.06 | -.13 | -.39 | -.13 |  |
|  |  | 2 | .46 | -.58 | .45 | -.78 | .49 | -.66 | .37 | .71 | -.14 | ─ | -.66 | -.05 | -.21 | -.59 | .10 |  |
|  |  | 3 | -.03 | -.46 | -.13 | .55 | .24 | -.33 | -.20 | .64 | -.82* | ─ | -.21 | -.05 | -.22 | .07 | .19 |  |
|  |  | 4 | -.66 | -.32 | -.47 | -.05 | .57 | -.10 | .19 | .79 | -.44 | ─ | -.71* | -.61 | -.47 | -.08 | .56 |  |
| 11. Social Competence | | 1 | -.66 | .22 | .04 | -.17 | -.06 | .01 | -.54 | -.02 | .68 | .34 | ─ | .54 | .80* | .62 | -.15 |  |
|  |  | 2 | -.76 | .81 | -.65 | .40 | -.27 | .32 | .15 | -.31 | .35 | -.43 | ─ | .53 | .75* | .56 | -.01 |  |
|  |  | 3 | .46 | .38 | .39 | .31 | -.28 | .08 | -.34 | -.02 | -.44 | .48 | ─ | .70 | .96** | .45 | -.04 |  |
|  |  | 4 | .60 | .18 | .35 | -.11 | -.53 | .06 | -.20 | -.69 | .46 | -.98** | ─ | .86** | .87** | .66 | -.58 |  |
| 12. School Competence | | 1 | -.51 | -.14 | .40 | -.30 | -.36 | .06 | -.65 | -.00 | .49 | .42 | .91** | ─ | .61 | .17 | .02 |  |
|  |  | 2 | -.64 | .68 | -.70 | .72 | -.20 | .31 | .22 | -.18 | -.01 | -.59 | .91* | ─ | .85** | .55 | .02 |  |
|  |  | 3 | .54 | .10 | .60 | .35 | -.23 | .09 | -.14 | -.14 | -.45 | .57 | .92** | ─ | .81* | .80* | .17 |  |
|  |  | 4 | .61 | .14 | .36 | -.24 | -.56 | .37 | -.06 | -.31 | .77 | -.81 | .87 | ─ | .83* | .61 | -.52 |  |
| 13. Emotional Competence | | 1 | -.46 | .12 | .16 | -.34 | .21 | -.38 | -.55 | .27 | .80* | .53 | .86* | .79* | ─ | .52 | -.13 |  |
|  |  | 2 | -.82* | .65 | -.57 | .09 | -.21 | .22 | .09 | -.26 | .53 | -.17 | .93** | .72 | ─ | .42 | .10 |  |
|  |  | 3 | .44 | .37 | .51 | -.05 | -.17 | -.08 | -.33 | -.24 | -.14 | .28 | .91* | .86* | ─ | .58 | -.13 |  |
|  |  | 4 | .31 | .03 | .17 | -.42 | -.23 | -.12 | .04 | -.59 | .36 | -.88* | .94* | .85 | ─ | .67 | -.41 |  |
| 14. Self-Perception | | 1 | -.23 | -.13 | .28 | .21 | .07 | .17 | .18 | .52 | .25 | .80* | .34 | .32 | .20 | ─ | .48 |  |
|  |  | 2 | .02 | -.29 | -.47 | .50 | .57 | -.46 | .62 | .57 | -.83* | -.00 | .11 | .42 | -.02 | ─ | -.15 |  |
|  |  | 3 | .52 | .40 | -.02 | .39 | .03 | -.17 | -.14 | .07 | -.51 | .28 | .82* | .60 | .72 | ─ | .29 |  |
|  |  | 4 | .79 | .25 | .39 | .52 | -.79 | .12 | -.68 | -.70 | .20 | -.78 | .71 | .44 | .44 | ─ | .06 |  |
| 15. Behavioral Problems | | 1 | .74 | -.70 | .45 | -.37 | -.32 | -.19 | .27 | -.57 | -.30 | -.29 | -.56 | -.37 | -.56 | -.28 | ─ |  |
|  |  | 2 | .23 | -.48 | .57 | -.12 | -.37 | .37 | -.79 | -.27 | .13 | -.11 | -.71 | -.66 | -.66 | -.45 | ─ |  |
|  |  | 3 | -.09 | .30 | .18 | -.18 | -.54 | .75 | .24 | -.47 | .59 | -.75 | -.56 | -.53 | -.58 | -.57 | ─ |  |
|  |  | 4 | -.15 | -.27 | -.31 | .38 | -.01 | .17 | -.37 | .71 | -.25 | .79 | -.80 | -.66 | -.91* | -.28 | ─ |  |
|  |  | | | | | | | | | | | | | | | | | |

*Note.* The results for the PC group are shown above the diagonal. The results for the CP group are shown below the diagonal.

**p*<.05. ***p*<.01. (significance tests must be cautiously treated due to small sample sizes)
