# Supplementary material for: Child training in the Child ViReal Support Program: Combining iVR-based cognitive training and CBT techniques in a pilot study
Source: PLoS One. 2026 Feb 27;21(2):e0343364. doi: 10.1371/journal.pone.0343364 (PMC12948055; doi:10.1371/journal.pone.0343364)
Supplement: S2 Table — (DOCX) [file pone.0343364.s004.docx]

| **S2 Table. Pearson correlation coefficients (Child Training assessment variables and dependent variables), for each time separately.** | | | | | | | | | | | | | |
| --- | --- | --- | --- | --- | --- | --- | --- | --- | --- | --- | --- | --- | --- |
|  |  | **Evaluation of Child Training – Final (ECT-F)** | | | | |  | **Evaluation of VR Experience – Final (EVR-F)** | | | | | |
|  |  | **Content / Structure** | | | **Utility/Feasibility** | |  | **VR Cybersickness Symptoms (SSQ)** | | |  |  |  |
|  | Time | Relationship with Therapist | Benefits from Program | Total Satisfaction | Program Usability | Program Acceptance | VR Total Time | VR  SSQ Nausea | VR  SSQ Oculomotor | VR_  Disorientation | VR  Sense of Presence | VR Enjoyment/ Engagement | VR Usability |
| ANT  Alerting Score | 1 | -.03 | -.42 | -.37 | -.17 | -.63* | -.24 | .23 | .33 | .07 | -.28 | -.02 | -.52 |
|  | 2 | .06 | -.04 | -.03 | .26 | -.37 | .05 | .02 | .21 | -.06 | .16 | .33 | -.31 |
|  | 3 | -.06 | .15 | .11 | .34 | -.22 | -.46 | -.21 | .28 | .01 | .09 | -.28 | -.29 |
|  | 4 | .16 | .30 | .30 | .24 | .14 | .15 | -.39 | .01 | -.61* | .36 | .35 | .28 |
| ANT  Orienting Score | 1 | .24 | -.09 | -.03 | -.68** | .04 | .34 | -.10 | .28 | -.38 | -.21 | -.18 | .04 |
|  | 2 | -.03 | .23 | .19 | .18 | .22 | .26 | .15 | .31 | -.18 | -.08 | -.34 | .28 |
|  | 3 | .35 | .36 | .38 | -.18 | .26 | .21 | -.08 | -.08 | -.56* | .33 | .54* | .32 |
|  | 4 | .33 | .24 | .28 | -.05 | .40 | .13 | -.27 | -.30 | .12 | .22 | .00 | .31 |
| ANT  Executive Score | 1 | .00 | -.17 | -.14 | .42 | .11 | .06 | -.06 | -.36 | .67** | .11 | -.19 | .04 |
|  | 2 | .42 | -.02 | .07 | .00 | -.01 | -.49 | -.02 | -.04 | .63* | .15 | -.27 | -.22 |
|  | 3 | .46 | .38 | .42 | .40 | .12 | -.11 | -.04 | .08 | -.36 | .09 | .25 | .13 |
|  | 4 | .33 | .33 | .36 | .39 | .43 | .22 | -.15 | -.36 | .16 | .24 | -.03 | .37 |
| Go/NoGo Omission Errors | 1 | .16 | -.06 | -.02 | .08 | -.13 | -.09 | -.11 | .42 | .53* | .23 | -.64* | -.26 |
|  | 2 | .15 | .19 | .19 | .15 | .28 | .01 | .00 | .04 | .62* | .45 | -.33 | .16 |
|  | 3 | .15 | .13 | .15 | .09 | .21 | -.14 | -.08 | .09 | .60* | .34 | -.44 | .06 |
|  | 4 | .11 | .19 | .19 | .13 | .41 | -.14 | -.03 | -.21 | .69** | .40 | -.32 | .25 |
| Go/NoGo Commission Errors | 1 | -.01 | .13 | .11 | -.47 | .13 | .03 | -.01 | .17 | -.23 | -.06 | .11 | .08 |
|  | 2 | -.05 | -.12 | -.12 | -.67** | .08 | -.12 | .08 | .08 | .26 | -.13 | -.19 | -.07 |
|  | 3 | .07 | .01 | .02 | -.27 | .01 | -.57* | -.04 | .16 | .44 | .08 | -.19 | -.22 |
|  | 4 | .03 | .01 | .01 | -.22 | .18 | -.45 | -.11 | -.25 | .33 | .13 | -.03 | .03 |
| Go/NoGo  Hit RT | 1 | .15 | .06 | .08 | .27 | -.12 | .01 | .16 | .13 | .49 | .29 | -.33 | -.19 |
|  | 2 | .19 | .26 | .26 | .71** | .26 | .17 | .17 | -.07 | .23 | .47 | .10 | .31 |
|  | 3 | .23 | .08 | .12 | -.06 | .23 | .54* | .65* | -.06 | -.12 | .27 | .51 | .42 |
|  | 4 | .02 | -.27 | -.23 | -.30 | .24 | .65* | .39 | -.14 | -.23 | -.19 | .19 | .43 |
| RTV | 1 | -.11 | .09 | .05 | .25 | .14 | -.06 | -.23 | .14 | .31 | .39 | -.21 | .13 |
|  | 2 | -.09 | .06 | .04 | -.47 | -.05 | -.15 | -.04 | .07 | .07 | -.11 | -.25 | -.09 |
|  | 3 | -.05 | -.05 | -.06 | .12 | -.04 | -.42 | -.06 | .27 | .69* | .09 | -.63* | -.19 |
|  | 4 | -.07 | .19 | .14 | .16 | .38 | -.29 | .11 | -.27 | .62* | .31 | -.20 | .26 |
| Working Memory Index (WMI) | 1 | .07 | -.21 | -.17 | -.27 | -.40 | .27 | .09 | -.05 | -.21 | -.26 | .19 | -.39 |
|  | 2 | -.05 | -.34 | -.30 | -.40 | -.34 | .26 | .11 | -.12 | -.24 | -.23 | .20 | -.27 |
|  | 3 | -.24 | -.24 | -.26 | -.35 | -.20 | .44 | .07 | -.24 | -.39 | -.13 | .28 | -.02 |
|  | 4 | -.24 | -.36 | -.36 | -.14 | -.46 | .35 | .14 | .06 | -.49 | -.25 | .30 | -.20 |
| Processing Speed Index (PSI) | 1 | .33 | .43 | .44 | .29 | .32 | -.11 | -.08 | -.08 | -.21 | .25 | .34 | .25 |
|  | 2 | .27 | .36 | .37 | .55* | .44 | .01 | -.04 | -.32 | -.03 | .08 | .15 | .39 |
|  | 3 | .12 | .13 | .14 | .05 | .38 | .05 | .03 | -.52 | -.10 | .22 | .51 | .47 |
|  | 4 | .34 | .32 | .35 | .32 | -.15 | .08 | -.09 | .01 | -.53 | .28 | .54 | -.06 |
| Tower | 1 | -.04 | -.37 | -.33 | -.39 | -.30 | .23 | .05 | .11 | -.20 | -.60* | -.18 | -.30 |
|  | 2 | -.39 | -.38 | -.40 | -.17 | -.39 | -.35 | -.18 | .02 | -.22 | -.59* | -.18 | -.38 |
|  | 3 | -.14 | -.13 | -.14 | -.21 | -.43 | .25 | -.21 | .16 | -.60* | -.37 | -.05 | -.34 |
|  | 4 | -.22 | -.38 | -.38 | -.33 | -.45 | .44 | -.09 | -.09 | -.50 | -.56* | .08 | -.28 |
| Social Competence | 1 | .65* | .52 | .58* | .28 | .54* | -.12 | .19 | -.07 | -.17 | .42 | .40 | .41 |
|  | 2 | .76** | .66* | .72** | .00 | .43 | .18 | .30 | .15 | -.20 | .53 | .44 | .30 |
|  | 3 | .79** | .80** | .85** | .16 | .33 | -.04 | .29 | .07 | -.18 | .57* | .45 | .16 |
|  | 4 | .70** | .74** | .78** | .28 | .49 | -.26 | .32 | .20 | .05 | .66* | .33 | .33 |
| School Competence | 1 | .67** | .35 | .44 | .16 | .18 | .17 | -.29 | -.09 | -.25 | .20 | .31 | .04 |
|  | 2 | .41 | .40 | .43 | -.05 | .07 | -.12 | .00 | .15 | -.64* | .34 | .64* | .14 |
|  | 3 | .54* | .68** | .70** | .42 | .15 | -.27 | -.11 | .03 | -.08 | .51 | .23 | .00 |
|  | 4 | .55 | .61* | .64* | .31 | .39 | -.16 | .30 | .20 | -.01 | .71** | .29 | .35 |
| Emotional Competence | 1 | .47 | .35 | .40 | .15 | .20 | -.21 | .06 | -.05 | -.38 | .22 | .56* | .14 |
|  | 2 | .59* | .56* | .60* | 11 | .22 | -.02 | 15 | .03 | -.49 | .34 | .67** | .17 |
|  | 3 | .69** | .87** | .89** | .25 | .45 | -.24 | .19 | -.06 | -.21 | .53 | .50 | .27 |
|  | 4 | .63* | .76** | .79** | .24 | .33 | -.30 | .16 | .23 | -.21 | .47 | .35 | .18 |
| Self-Perception | 1 | .44 | .26 | .32 | .39 | .34 | .08 | -.11 | -.111 | -.14 | .46 | .27 | .44 |
|  | 2 | .39 | -.03 | .06 | -.39 | .19 | .30 | -.28 | .103 | -.41 | .18 | .32 | .39 |
|  | 3 | .37 | .58* | .58* | .11 | .33 | -.19 | -.01 | -.071 | -.07 | .57* | .26 | .37 |
|  | 4 | .81** | .64* | .72** | .15 | .33 | -.04 | .08 | .076 | -.24 | .62* | .51 | .81** |
| Behavioral Problems | 1 | .03 | -.06 | -.05 | .34 | 02 | -.16 | -.07 | -.27 | .26 | .12 | .05 | .03 |
|  | 2 | -.13 | -.16 | -.16 | .05 | .03 | .05 | .35 | -.41 | .21 | .10 | .29 | -.13 |
|  | 3 | -.02 | -.03 | -.04 | .27 | .02 | .10 | .18 | -.21 | .33 | .28 | .07 | -.02 |
|  | 4 | -.14 | -.08 | -.10 | .13 | -.26 | .20 | -.18 | -.26 | .09 | -.01 | -.08 | -.14 |
| **p*<.05. ***p*<.01. (significance tests must be cautiously treated, due to small sample sizes) | | | | | | | | | | | | | |
