# Supplementary material for: Child training in the Child ViReal Support Program: Combining iVR-based cognitive training and CBT techniques in a pilot study
Source: PLoS One. 2026 Feb 27;21(2):e0343364. doi: 10.1371/journal.pone.0343364 (PMC12948055; doi:10.1371/journal.pone.0343364)

**Determinants of Treatment Response to State-of-the-art Interventions for Attention Deficits: Child Temperament, Cognitive Profiles and Family Dynamics**

Ιουλιανή Παχίτη

Τμήμα Ψυχολογίας, Πανεπιστήμιο Κρήτης

Επιστημονικά Υπεύθυνη: Δρ. Παναγιώτα Δημητροπούλου

Επίκουρη Καθηγήτρια Εκπαιδευτικής Ψυχολογίας

**Ερευνητικό Πρωτόκολλο**

Οι δυσκολίες προσοχής που παρουσιάζουν αρκετά παιδιά κατά την προσχολική και σχολική ηλικία και οι οποίες συχνά συνοδεύονται από συμπτώματα υπερκινητικότητας και παρορμητικότητας ορίζονται από το Διαγνωστικό και Στατιστικό Εγχειρίδιο Ψυχικών Διαταραχών-5 (DSM-V; American Psychiatric Association, 2013) ως Διαταραχή Ελλειμματικής Προσοχής με/ή χωρίς Υπερκινητικότητα (ΔΕΠ-Υ) με την προϋπόθεση ότι τα παιδιά πληρούν συγκεκριμένα κριτήρια διάγνωσης. Η ΔΕΠ-Υ συνιστά μια χρόνια νευροαναπτυξιακή διαταραχή, η οποία αποτελεί μια από τις πιο συχνά διαγνωσμένες αναπτυξιακές διαταραχές στην παιδική ηλικία (Maniadaki, 2019. Sekaninova και συν., 2019). Χαρακτηρίζεται από ένα επίμονο και αναπτυξιακά μη κατάλληλο μοτίβο δυσκολιών προσοχής, υπερκινητικότητας και παρορμητικότητας με αποτέλεσμα τα άτομα με ΔΕΠ-Υ να παρουσιάζουν δυσκολίες στη συγκέντρωση, τη διατήρηση της προσοχής, τη μνήμη εργασίας και τον έλεγχο των παρορμήσεων (American Psychiatric Association, 2013. Maniadaki, 2019. Sekaninova και συν., 2019).

Σύμφωνα με τα επιδημιολογικά στοιχεία διαφόρων ερευνών, τα ποσοστά εμφάνισης της συγκεκριμένης διαταραχής κυμαίνονται μεταξύ 3-7% στον παιδικό πληθυσμό, ενώ σε μετα-ανάλυση 102 ερευνών που πραγματοποίησαν ο Polanczyk και οι συνεργάτες του (2007) φάνηκε ότι το παγκόσμιο ποσοστό της ΔΕΠ-Υ στον παιδικό πληθυσμό είναι 5,29% (Μανιαδάκη & Κάκουρος, 2016. Polanczyk και συν., 2007). Όσον αφορά στον ελληνικό πληθυσμό, τα ερευνητικά δεδομένα αναφέρουν ποσοστά μεταξύ 5-11% (Μανιαδάκη & Κάκουρος, 2016). Μια από τις σημαντικότερες επιδημιολογικές έρευνες που έγιναν μέχρι σήμερα στην Ελλάδα, όπου μελετήθηκε η συχνότητα εμφάνισης της συμπτωματολογίας της ΔΕΠ-Υ σε 2695 παιδιά τα οποία οι ερευνητές παρακολούθησαν συστηματικά από τη γέννηση μέχρι την ενηλικίωσή τους, κατέδειξε ότι στην ηλικία των 7 ετών η συχνότητα των συμπτωμάτων υπερκινητικότητας έφτανε το 7%, των δυσκολιών προσοχής το 9,5% και της παρορμητικότητας το 7%. Η ίδια ομάδα παιδιών αξιολογήθηκε στην ηλικία των 18 ετών και φάνηκε ότι τα συμπτώματα της υπερκινητικότητας και της παρορμητικότητας μειώθηκαν στο 3% και 0,3% αντίστοιχα, ωστόσο οι δυσκολίες προσοχής εξακολουθούσαν να είναι παρούσες στο 7,3% του δείγματος (Palili και συν., 2011).

Οι δυσκολίες των παιδιών που διαγιγνώσκονται με ΔΕΠ-Υ τις περισσότερες φορές παρουσιάζουν συννοσηρότητα και με άλλες διαταραχές και δυσκολίες, όπως διασπαστικές συμπεριφορές, μαθησιακές δυσκολίες, εσωτερικευμένα προβλήματα (άγχος, κατάθλιψη), καθώς επίσης και δυσκολίες δημιουργίας κοινωνικών σχέσεων, απόρριψη από την ομάδα των συνομηλίκων, μειωμένη σχολική επίδοση και δυσκολίες στον ύπνο (Brassett-Harknett & Butler, 2005. Tarver και συν., 2014). Επίσης, τα παιδιά με ΔΕΠ-Υ φαίνεται να παρουσιάζουν ελλείμματα στις επιτελικές λειτουργίες τα οποία είναι σε αντιστοιχία με τις δομικές διαφορές που παρατηρούνται στις εμπρόσθιες περιοχές του εγκεφαλικού τους φλοιού, όπως αυτές προκύπτουν από μελέτες με απεικονιστικές μεθόδους. Τα ελλείμματα αυτά στις επιτελικές λειτουργίες γίνονται εμφανή και από τις χαμηλότερες επιδόσεις που έχουν τα παιδιά με ΔΕΠ-Υ σε σύγκριση με τυπικά αναπτυσσόμενα παιδιά σε έργα που μετρούν διαστάσεις των επιτελικών αυτών λειτουργιών (π.χ. εστιασμένη προσοχή, μνήμη εργασίας κτλ.) (Sjöwall και συν., 2013. Tarver και συν., 2014).

Επιπρόσθετα, η δυσκολία αναγνώρισης, ελέγχου και διαχείρισης των συναισθημάτων αποτελεί ένα κοινό χαρακτηριστικό των ατόμων που διαγιγνώσκονται με ΔΕΠ-Υ. Οι δυσκολίες αυτές επισημαίνονται τόσο από τους γονείς οι οποίοι αναφέρουν ότι τα παιδιά τους παρουσιάζουν χαμηλά επίπεδα συναισθηματικού ελέγχου και υψηλά επίπεδα αρνητικών συναισθημάτων (υψηλά επίπεδα θυμού, εκνευρισμού), όσο και μέσα από μετρήσεις κατά τη διάρκεια έργων αναγνώρισης συναισθημάτων (Franke και συν., 2018. Sjöwall και συν., 2013).

Σε γενικές γραμμές, υπάρχει σημαντική ετερογένεια μεταξύ των ατόμων που παρουσιάζουν δυσκολίες προσοχής και υπερκινητικότητα τόσο όσον αφορά στα νευροψυχολογικά ελλείμματα όσο και στις συναισθηματικές δυσκολίες καθιστώντας απαραίτητη την στοχευμένη παρέμβαση στα ελλείμματα που παρουσιάζει το κάθε παιδί. Επιπλέον είναι σημαντικό οι παρεμβάσεις να στοχεύουν και στη συναισθηματική λειτουργικότητα των παιδιών, καθώς αποτελεί ένα βασικό παράγοντα στην πρόβλεψη αρνητικών γεγονότων στη ζωή τους συμπεριλαμβανομένων της παραίτησης από το σχολείο και των δυσκολιών στις οικογενειακές σχέσεις (Franke και συν., 2018. Sjöwall και συν., 2013. Tarver και συν., 2014).

Όσον αφορά στην εξελικτική πορεία των δυσκολιών των παιδιών με ΔΕΠ-Υ, φαίνεται μέσα από ερευνητικά δεδομένα διαχρονικών μελετών ότι σε αρκετά άτομα οι δυσκολίες προσοχής διατηρούνται και κατά τη διάρκεια της εφηβείας και της ενηλικίωσης παρόλο που παρατηρείται μείωση των επιπέδων της υπερκινητικότητας/ παρορμητικότητας (Brassett-Harknett & Butler, 2007. Franke και συν., 2018. Palili και συν., 2011). Παράλληλα, παρατηρούνται αρκετά συνοδά προβλήματα, όπως είναι η μεγαλύτερη πιθανότητα για ανάπτυξη εθισμών σε ουσίες, ιδιαίτερα στις περιπτώσεις που υπάρχει συννοσηρότητα με διασπαστικές συμπεριφορές (π.χ. διαταραχή διαγωγής), το αυξημένο ρίσκο για εκδήλωση βίαιων συμπεριφορών και προβλημάτων με το νόμο, καθώς επίσης και περισσότερα προβλήματα οδήγησης ή περισσότερα αυτοκινητικά ατυχήματα (Brassett-Harknett & Butler, 2007. Franke και συν., 2018).

Παράλληλα με τις ενδοατομικές δυσκολίες που παρουσιάζουν τα παιδιά που διαγιγνώσκονται με ΔΕΠ-Υ παρατηρούνται και αλλαγές στις οικογενειακές σχέσεις, οι οποίες πολλές φορές διαταράσσονται στο άκουσμα της διάγνωσης για το παιδί. Μετασυνθέσεις και μετα-αναλύσεις ποιοτικών και ποσοτικών ερευνών αντίστοιχα κατέδειξαν ότι οι γονείς των παιδιών με διάγνωση ΔΕΠ-Υ παρουσιάζουν υψηλά επίπεδα γονεϊκού στρες και βιώνουν αρκετά έντονα και αρνητικά συναισθήματα στην προσπάθειά τους να διαχειριστούν την οικογενειακή ρουτίνα και τις συμπεριφορές των παιδιών τους (Corcoran και συν., 2017. Miller & Brooker, 2017. Theule και συν., 2013). Ακόμη και οι τυπικές οικογενειακές ρουτίνες (π.χ. ετοιμασία για το σχολείο, ετοιμασία για ύπνο) αποτελούν καθημερινή πρόκληση για τους γονείς, ενώ το γονεϊκό στρες φαίνεται να διοχετεύεται σε αρκετούς τομείς της ζωής τους, όπως είναι η υγεία τους, η ψυχολογική, συζυγική και επαγγελματική τους λειτουργικότητα. Σε αρκετές έρευνες που αναφέρονται στη μετασύνθεση ερευνών που διεξήγαγαν οι Corcoran και συνεργάτες (2017), αρκετοί γονείς ανέφεραν ότι χρειάστηκε να αλλάξουν ή να παραιτηθούν από τη δουλειά τους προκειμένου να μπορέσουν να διαχειριστούν καλύτερα τις συμπεριφορές των παιδιών τους (Hallberg και συν., 2008. Ηο και συν., 2011. Moen και συν., 2011, όπως αναφέρονται στο Corcoran και συν., 2017), ενώ παρατηρείται και διατάραξη των συζυγικών σχέσεων για διάφορους λόγους (π.χ. το παιδί αναζητά συνεχώς προσοχή και μειώνεται ο χρόνος που έχουν μεταξύ τους οι γονείς, οι μητέρες αναλαμβάνουν περισσότερο ρόλο στη διαχείριση των συμπεριφορών των παιδιών σε σχέση με τους πατέρες κτλ.) (Corcoran και συν., 2017. Theule και συν., 2013). Επιπλέον, διαταράσσονται και οι σχέσεις των γονέων με το παιδί τους, οι οποίες χαρακτηρίζονται από περισσότερες συγκρούσεις και περισσότερο αυταρχικές και ελεγκτικές τεχνικές γονικού ελέγχου των παιδιών (Lifford και συν., 2008). Επομένως, φαίνεται ότι είναι σημαντικό να παρέχεται στήριξη και βοήθεια στους γονείς των παιδιών που έχουν λάβει διάγνωση ΔΕΠ-Υ όσον αφορά σε δεξιότητες συναισθηματικής αυτοδιαχείρισης, καθώς βιώνουν έντονο στρες και αρνητικά συναισθήματα, καθώς επίσης και σε κατάλληλες γονεϊκές πρακτικές διαχείρισης των συμπεριφορών των παιδιών τους.

**Πρόγραμμα Κριτηρίων Ερευνητικού Τομέα (Research Domain of Criteria, RDoC)**

Tα τελευταία χρόνια έχει αναπτυχθεί μια νέα ερευνητική προσπάθεια από το National Institute of Mental Health (NIMH) σε μια προσπάθεια κατανόησης των δυσκολιών που παρουσιάζουν τα άτομα με διαταραχές μέσα από μια πολυεπίπεδη προσέγγιση σε αντίθεση με την κατηγορική προσέγγιση που ακολουθείται από το DSM και το ICD, η οποία αναφέρεται ως Πρόγραμμα Κριτηρίων Ερευνητικού Τομέα (Research Domain of Criteria, RDoC) (Cuthbert, 2015). Η συγκεκριμένη ερευνητική προσπάθεια δεν στοχεύει να αντικαταστήσει τα δύο υπάρχοντα διαγνωστικά εγχειρίδια και να αποτελέσει ένα νέο διαγνωστικό κατηγορικό σύστημα. Στόχος της είναι η μελέτη και η σύνδεση πληροφοριών από διαφορετικά επίπεδα (από γονιδιωματικές πληροφορίες μέχρι και αυτο-αναφορές) σχετικά με τις δυσκολίες που παρουσιάζουν τα άτομα μέσα από μια πολυεπίπεδη προσέγγιση του εύρους της λειτουργικότητας των νευροβιολογικών, γνωστικών και συμπεριφορικών ικανοτήτων παρουσιάζοντάς τα σε ένα συνεχές υψηλότερων ή χαμηλότερων βαθμών υγείας ή προσαρμογής (Musser & Raiker, 2019. Garvey και συν., 2016).

Επίσης, μέσα από τις ερευνητικές προσπάθειες που υιοθετούν την προσέγγιση του RDoC, στόχος είναι να δημιουργηθούν κατάλληλα εννοιολογικά μοντέλα που θα παρέχουν την απαραίτητη γνώση μεταξύ και εντός αυτών των επιπέδων των πληροφοριών (γονίδια, μόρια, κύτταρα, κυκλώματα, φυσιολογία, συμπεριφορά και αυτο-αναφορές). Με τον τρόπο αυτό επιδιώκεται η καλύτερη κατανόηση της πολυπλοκότητας των διαφόρων διαταραχών στο επίπεδο των συμπτωμάτων και η ανάπτυξη κατάλληλων και πιο αποτελεσματικών φαρμακευτικών και συμπεριφορικών παρεμβάσεων που να βασίζονται και στη βιολογική βάση των δυσκολιών (Garvey και συν., 2016).

Επομένως, οι νέες ερευνητικές προσπάθειες κατανόησης των δυσκολιών που παρουσιάζουν τα παιδιά με ΔΕΠ-Υ είναι σημαντικό να εμπλουτίζονται και από μετρήσεις που δεν περιορίζονται μόνο στις αναφορές των γονέων, των εκπαιδευτικών ή σε αυτο-αναφορές των εφήβων σε κλίμακες συμπεριφοράς, αλλά που δίνουν δεδομένα σχετικά και με τη νευροβιολογική βάση της συμπεριφοράς (π.χ. απεικονιστικές μέθοδοι, fMRI, οφθαλμοκίνηση κτλ.) προκειμένου να υπάρχει σύνδεση μεταξύ των επιπέδων.

Ένα σύστημα που μπορεί να δώσει τέτοιου είδους δεδομένα, στην περίπτωση των ατόμων με ΔΕΠ-Υ, είναι το οπτικοκινητικό σύστημα, καθώς μέσα από τις μετρήσεις με έναν καταγραφέα οφθαλμοκίνησης (eye-tracker) λαμβάνονται πληροφορίες τόσο για την αντανακλαστική όσο και για την εκούσια συμπεριφορά (Luna και συν., 2008. Rommelse και συν., 2008). Η εκτέλεση μιας οφθαλμικής κίνησης είναι το αποτέλεσμα μιας πολύπλοκης αλληλεπίδρασης διάφορων γνωστικών διαδικασιών και έτσι, η οφθαλμοκινητική συμπεριφορά μπορεί να παρέχει πληροφορίες σχετικά με τις διαδικασίες αυτές, αλλά και με ελλείμματα που παρατηρούνται (Rommelse και συν., 2008). Επιπλέον, με την προσθήκη γνωστικών απαιτήσεων σε οφθαλμοκινητικά έργα (π.χ. έργα με αντισακκαδικές κινήσεις), οι εκούσιες οφθαλμικές κινήσεις απαιτούν τη χρήση υψηλότερου επιπέδου γνωστικών διαδικασιών, καθώς παράγουν νευρωνική δραστηριότητα σε ολόκληρο τον εγκεφαλικό φλοιό εν αναμονή μιας προγραμματισμένης αντίδρασης, επιτρέποντας με αυτό τον τρόπο να αναγνωριστούν οι εγκεφαλικές δομές που παίζουν ρόλο στις γνωστικές αυτές διεργασίες (Basso, 1998, όπως αναφέρεται στο Luna και συν., 2008).

**Θεραπευτική Παρέμβαση και ΔΕΠ-Υ**

Με βάση όσα έχουν προαναφερθεί, παρατηρείται ότι οι δυσκολίες προσοχής και υπερκινητικότητας/ παρορμητικότητας, καθώς και τα συνοδά με αυτές προβλήματα εμφανίζονται σε ένα σημαντικό ποσοστό του παιδικού πληθυσμού και επηρεάζουν τα άτομα σε σημαντικούς τομείς της ζωής τους, αλλά και τις ενδοοικογενειακές σχέσεις. Επομένως, γίνεται εμφανές ότι τόσο τα παιδιά που έχουν λάβει διάγνωση ΔΕΠ-Υ, όσο και οι γονείς τους χρειάζονται κατάλληλη στήριξη και παρέμβαση με στόχο τη διαχείριση των συμπεριφορών που εκδηλώνουν τα παιδιά, αλλά και τη συναισθηματική αυτοδιαχείριση των γονέων που καλούνται να αντιμετωπίσουν τις συμπεριφορές αυτές.

Παρά το γεγονός ότι δεν υπάρχει αποθεραπεία για τη ΔΕΠ-Υ, έχουν σχεδιαστεί και εφαρμοστεί παρεμβάσεις οι οποίες στοχεύουν στη διαχείριση των συμπτωμάτων που παρατηρούνται (Schellack και συν., 2019. Tarver και συν., 2014). Οι σύγχρονες εμπειρικά βασισμένες παρεμβάσεις που θεωρούνται ευρέως αποδεκτές για την αντιμετώπιση της ΔΕΠ-Υ περιλαμβάνουν τη φαρμακευτική αγωγή, τις ψυχοπαιδαγωγικές παρεμβάσεις ή ένα συνδυασμό των δύο (Anton και συν., 2009. Μανιαδάκη & Κάκουρος, 2016). Η φαρμακευτική αγωγή περιλαμβάνει διεγερτικά (π.χ. μεθυλφαινιδάτη και αμφεταμίνη) και μη-διεγερτικά φάρμακα (π.χ. ατομοξετίνη). Ερευνητικά δεδομένα καταδεικνύουν την αποτελεσματικότητα της φαρμακοθεραπείας όσον αφορά στη μείωση των πρωτογενών συμπτωμάτων της ΔΕΠ-Υ, καθώς τα διεγερτικά φαίνεται να βελτιώνουν την αναστολή και την αυτορρύθμιση της συμπεριφοράς, ενώ τα μη διεγερτικά να βοηθούν στη βελτίωση της ικανότητας προσοχής και συγκέντρωσης (Μανιαδάκη & Κάκουρος, 2016. Schellack και συν., 2019. Tarver και συν., 2014).

Παρόλα αυτά, αρκετοί ειδικοί και γονείς ανησυχούν για τις πιθανές παρενέργειες που μπορεί να έχει η φαρμακοθεραπεία στα παιδιά. Κάποιες από αυτές τις παρενέργειες είναι η μείωση της όρεξης, η διαταραχή του ύπνου, οι πονοκέφαλοι, οι ναυτίες, η αίσθηση κόπωσης, καθώς και οι αλλαγές στη διάθεση (Franke και συν., 2018. Μανιαδάκη & Κάκουρος, 2016. Zachor και συν., 2009). Επιπλέον, η φαρμακευτική αγωγή μπορεί μεν να βοηθά στη βελτίωση των πρωτογενών συμπτωμάτων της ΔΕΠ-Υ με παράλληλη βραχυπρόθεσμη βελτίωση στις κοινωνικές αλληλεπιδράσεις των παιδιών και στις σχολικές τους επιδόσεις, εντούτοις η βελτίωση αυτή δεν διατηρείται μακροπρόθεσμα και δεν συμβάλλει στη γενικότερη λειτουργικότητά τους στις κοινωνικές σχέσεις και τη σχολική ζωή (Μανιαδάκη & Κάκουρος, 2016. Tarver και συν., 2014).

Όσον αφορά στις ψυχοπαιδαγωγικές παρεμβάσεις, αυτές περιλαμβάνουν: α) τα προγράμματα τροποποίησης της συμπεριφοράς με βασικά συστατικά αυτών των προγραμμάτων την εκπαίδευση των γονέων και τις παρεμβάσεις στο σχολείο, β) τις γνωστικο-συμπεριφορικές παρεμβάσεις και γ) την εκπαίδευση στην απόκτηση κοινωνικών δεξιοτήτων (Anton και συν., 2009. Μανιαδάκη & Κάκουρος, 2016. Tarver και συν., 2014).

Οι παρεμβάσεις τροποποίησης της συμπεριφοράς βασίζονται στις αρχές της συντελεστικής και της κοινωνικής μάθησης και στοχεύουν στη μείωση των ανεπιθύμητων μορφών συμπεριφοράς, την αύξηση των επιθυμητών μορφών συμπεριφοράς και την ανάπτυξη νέων λειτουργικών μορφών συμπεριφοράς που δεν υπήρχαν προηγουμένως στο ρεπερτόριο του παιδιού. Η εφαρμογή τους στηρίζεται κυρίως στην εκπαίδευση των γονέων και των εκπαιδευτικών σε τεχνικές που βασίζονται στη διαφορική ενίσχυση, καθώς και σε συστήματα επιβράβευσης (π.χ. συστήματα ανταλλάξιμων αμοιβών, κοινωνικοί ενισχυτές, time-out κτλ.) (Anton και συν., 2009. Μανιαδάκη & Κάκουρος, 2016. Zachor και συν., 2009).

Βασικό στόχο της εκπαίδευσης των γονέων αποτελεί η καλύτερη κατανόηση και αποσαφήνιση των δυσκολιών που βιώνουν τα παιδιά, η αντιμετώπιση τυχόν λανθασμένων αντιλήψεων που μπορεί να έχουν σχετικά με τον εαυτό τους και τα παιδιά τους, καθώς επίσης και η αντικατάσταση μη λειτουργικών μοτίβων αλληλεπίδρασης που έχουν με τα παιδιά τους με πιο λειτουργικά και θετικά μοτίβα (Johnston & Park, 2015. Lee και συν., 2012. Pfiffner & Haack, 2014). Ουσιαστικά, εκπαιδεύονται να εστιάζουν και στις θετικές συμπεριφορές των παιδιών, εκτός από τις αρνητικές στις οποίες συνήθως δίνουν ιδιαίτερη σημασία, να καθορίζουν τους κανόνες και να δίνουν τις οδηγίες, όπως και να χρησιμοποιούν τις τεχνικές διαφορικής ενίσχυσης προκειμένου να μάθουν να χειρίζονται αποτελεσματικά τις αντιδράσεις του παιδιού τους. Ακόμη, εκπαιδεύονται στην εφαρμογή συστημάτων ανταλλάξιμων αμοιβών (π.χ. λίστα με πράγματα που αρέσουν στο παιδί να κάνει και για τα οποία πρέπει να κερδίσει συγκεκριμένο αριθμό βαθμών), αλλά και στο πώς να γενικεύσουν τις τεχνικές αυτές και σε συμπεριφορές που εκδηλώνονται εκτός του σπιτιού (Anton και συν., 2009. Johnston & Park, 2015. Μανιαδάκη & Κάκουρος, 2016. Lee και συν., 2012. Zachor και συν., 2009). Η εκπαίδευση των γονέων σε προγράμματα τροποποίησης της συμπεριφοράς φαίνεται να βελτιώνει τη λειτουργικότητα του παιδιού, των γονέων και του συνόλου της οικογένειας, καθώς επίσης και την αίσθηση αυτοαποτελεσματικότητάς τους όσον αφορά στις δεξιότητες διαχείρισης των συμπεριφορών που εκδηλώνουν τα παιδιά τους. Επιπλέον, φαίνεται να παρατηρείται αύξηση της γονεϊκής αυτοπεποίθησης, βελτίωση των γονεϊκών πρακτικών και μείωση του γονεϊκού στρες και των αρνητικών τους συναισθημάτων (Pfiffner & Haack, 2014. Zachor και συν., 2009).

Παράλληλα με τα παραπάνω, τα τελευταία χρόνια έχει δοθεί ιδιαίτερη έμφαση στην έννοια της γονεϊκής ενσυνειδητότητας (mindful parenting) και την εκπαίδευση των γονέων σε παρεμβάσεις βασισμένες στην ενσυνειδητότητα (mindfulness-based interventions) (Miller & Brooker, 2017). Η ενσυνειδητότητα αναφέρεται στην επίγνωση που προκύπτει μέσα από τη σκόπιμη εστίαση της προσοχής μας στην παρούσα στιγμή, και την παρατήρηση της τρέχουσας εμπειρίας, όπως αυτή προκύπτει, χωρίς να την κρίνουμε, να την αναλύουμε ή να την αξιολογούμε με οποιοδήποτε τρόπο (Han και συν., 2019). Όσον αφορά στη γονεϊκή ενσυνειδητότητα, μέσα από ερευνητικά δεδομένα που μελετούν την πορεία των επιδράσεών της στα προβλήματα εσωτερίκευσης και εξωτερίκευσης των παιδιών προκύπτει ότι οι γονείς με γονεϊκή ενσυνειδητότητα χρησιμοποιούν περισσότερο θετικές γονεϊκές πρακτικές. Επίσης, μπορούν καλύτερα να διαχωρίζουν τις γνωστικές, συναισθηματικές και συμπεριφορικές εμπειρίες σε σύγκριση με τους γονείς που έχουν χαμηλότερα επίπεδα γονεϊκής ενσυνειδητότητας μειώνοντας με αυτό τον τρόπο την πιθανότητα εμπλοκής τους σε μη κατάλληλες αλληλεπιδράσεις με τα παιδιά τους (Han και συν., 2019).

Ειδικότερα, οι γονείς που χρησιμοποιούν τεχνικές γονεϊκής ενσυνειδητότητας και αλληλεπιδρούν ενσυνείδητα και προσεγμένα με τα παιδιά τους έχουν υψηλότερου επιπέδου ποιότητα σχέσης με αυτά σε σχέση με τους γονείς που έχουν λιγότερο ενσυνείδητες αλληλεπιδράσεις (Duncan και συν., 2009, όπως αναφέρεται στο Han και συν., 2019. Parent και συν., 2016). Το παραπάνω σχετίζεται με καλύτερη ψυχοκοινωνική προσαρμογή και λιγότερα προβλήματα συμπεριφοράς στα παιδιά (Han και συν., 2019. Parent και συν., 2016). Επομένως, οι παρεμβάσεις που βασίζονται στην ενσυνειδητότητα έχουν ενταχθεί στα προγράμματα παρέμβασης για γονείς παιδιών με ΔΕΠ-Υ τα τελευταία χρόνια, εφόσον φαίνεται ότι προσφέρουν ευκαιρίες για αυτο-φροντίδα που χρειάζονται οι γονείς, καθώς εξασκούνται σε τεχνικές που τους βοηθούν σε διαχείριση του στρες, σε μείωση της δυσφορίας/θλίψης και σε μείωση συμπτωμάτων ψυχοπαθολογίας (Miller & Brooker, 2017).

Όσον αφορά στις γνωστικο-συμπεριφορικές παρεμβάσεις, αυτές αποτελούν μια επέκταση των συμπεριφορικών μεθόδων τροποποίησης της συμπεριφοράς, η οποία εστιάζει στην αλληλεπίδραση μεταξύ των γνωστικών λειτουργιών, των συναισθημάτων και της συμπεριφοράς των ατόμων (Flores & Parra, 2014. Tarver και συν., 2014). Ο στόχος της γνωστικο-συμπεριφορικής παρέμβασης είναι να μάθουν τα παιδιά να ασκούν αποτελεσματικότερο έλεγχο στις σκέψεις, τα συναισθήματα και τις γνωστικές τους λειτουργίες ως ένα μέσο για αυτοδιαχείριση και βελτίωση της ικανότητας αυτοελέγχου της συγκέντρωσης της προσοχής τους και της συμπεριφοράς τους. Οι γνωστικο-συμπεριφορικές παρεμβάσεις περιλαμβάνουν τεχνικές αυτοκαθοδήγησης, αυτοενίσχυσης, τεχνικές επίλυσης προβλημάτων και παιχνίδια ρόλων. Έτσι, τα παιδιά με ΔΕΠ-Υ μαθαίνουν στρατηγικές με τις οποίες αυτοαξιολογούν και αυτοδιορθώνουν τη συμπεριφορά τους, καθώς και τεχνικές βελτίωσης των γνωστικών τους δεξιοτήτων όπως της προσοχής και της μνήμης εργασίας. Αυτές οι στρατηγικές έχουν αντίκτυπο σε διάφορους τομείς της ζωής τους, όπως είναι η προσαρμογή στους κανόνες και τις ρουτίνες της καθημερινότητας, η ανάπτυξη κοινωνικών σχέσεων και υγιούς αυτοεκτίμησης και η καλύτερη επίλυση προβλημάτων (Flores & Parra, 2014. Μανιαδάκη & Κάκουρος, 2016. Schellack και συν., 2019).

Εκτός από τις παραπάνω μορφές παρέμβασης, ιδιαίτερα σημαντική είναι η εκπαίδευση των παιδιών με ΔΕΠ-Υ στην απόκτηση κοινωνικών δεξιοτήτων (social skills training), καθώς όπως αναφέρθηκε πιο πάνω, τα παιδιά με ΔΕΠ-Υ παρουσιάζουν δυσκολίες στη σύναψη και διατήρηση φιλικών σχέσεων, ενώ συχνά βιώνουν και την απόρριψη από την ομάδα των συνομηλίκων. Η εκπαίδευση στην απόκτηση κοινωνικών δεξιοτήτων περιλαμβάνει παιχνίδια ρόλων σε ένα ευρύ φάσμα δεξιοτήτων, όπως είναι η διατύπωση ερωτήσεων, η συνεργασία, η διατήρηση μιας συζήτησης, η τήρηση κανόνων και η ένταξη σε μια ομάδα συνομηλίκων (Μανιαδάκη & Κάκουρος, 2016. Pfiffner & Haack, 2014. Zachor και συν., 2009). Οι έρευνες που πραγματοποιήθηκαν σχετικά με την αποτελεσματικότητα των συγκεκριμένων παρεμβάσεων δεν επέδειξαν σημαντικά αποτελέσματα και ένας πιθανός λόγος για αυτό είναι ότι δεν μεταφέρονται και δεν γενικεύονται οι δεξιότητες που μαθαίνονται στο πλαίσιο της παρέμβασης και στην καθημερινότητα του παιδιού (Evans και συν., 2014).

Συνοψίζοντας σχετικά με τα υπάρχοντα εμπειρικά βασισμένα είδη παρέμβασης για τα παιδιά με ΔΕΠ-Υ, φαίνεται ότι η φαρμακευτική αγωγή μπορεί να βοηθήσει στη μείωση των πρωτογενών συμπτωμάτων της ΔΕΠ-Υ, χωρίς όμως να παρατηρείται μακροπρόθεσμη βελτίωση στη λειτουργικότητα των παιδιών. Οι ψυχοκοινωνικές παρεμβάσεις από την άλλη, βοηθούν τόσο τους γονείς όσο και τα παιδιά να αποκτήσουν δεξιότητες για τη διαχείριση των δυσκολιών που παρουσιάζονται και η αποτελεσματικότητά τους έχει διαφανεί από μετα-αναλύσεις ερευνών (Johnston & Park, 2015. Fabiano και συν., 2009, όπως αναφέρεται στο Tarver και συν., 2014). Παρόλο που σύμφωνα με μια πρόσφατη μετα-ανάλυση ερευνών από τον Sonuga και τους συνεργάτες του (2013, όπως αναφέρεται στα Franke και συν., 2018 και Tarver και συν., 2014) φάνηκε ότι τα μεγέθη επίδρασης των ψυχοπαιδαγωγικών παρεμβάσεων για τη μείωση των κύριων συμπτωμάτων της ΔΕΠ-Υ ήταν χαμηλά, εντούτοις είναι σημαντικό να λαμβάνεται υπόψη ότι οι παρεμβάσεις αυτές έχουν θετικά αποτελέσματα τόσο όσον αφορά στη διαχείριση των συμπεριφορών των παιδιών, όσο και σε δευτερογενή συμπτώματα και δυσκολίες, όπως είναι η βελτίωση των κοινωνικών αλληλεπιδράσεών τους, η μείωση του γονεϊκού στρες και η βελτίωση της αίσθησης γονεϊκής αυτοαποτελεσματικότητας, καθώς και η βελτίωση της ικανότητας αυτοελέγχου της συμπεριφοράς από τα παιδιά με αποτέλεσμα τη μακροπρόθεσμη προσαρμογή τους στο περιβάλλον (Anton και συν., 2009. Μανιαδάκη & Κάκουρος, 2016. Pfiffner & Haack, 2014).

Η αποτελεσματικότερη βελτίωση των δυσκολιών που παρουσιάζουν τα παιδιά με ΔΕΠ-Υ φαίνεται ότι σχετίζεται με πολυδιάστατα παρεμβατικά προγράμματα, τα οποία εξατομικεύονται με βάση τα γνωστικά και συμπεριφορικά επίπεδα του κάθε παιδιού. Τα προγράμματα αυτά αξιοποιούν το συνδυασμό της ψυχοεκπαίδευσης των γονέων και της γνωστικο-συμπεριφορικής παρέμβασης που βασίζεται στο παιδί (Anton και συν., 2009. Μανιαδάκη & Κάκουρος, 2016. Rajeh και συν., 2017) και σε αρκετές περιπτώσεις και τη φαρμακευτική αγωγή.

Μια σημαντική διάσταση που φαίνεται ότι απουσιάζει, βάσει των νεότερων ερευνητικών δεδομένων, είναι η ενίσχυση των παραπάνω προγραμμάτων παρέμβασης με την εκμάθηση δεξιοτήτων από τα παιδιά σε ένα περιβάλλον όμοιο με αυτό στο οποίο αναμένεται να μεταφερθούν και να γενικευθούν οι συγκεκριμένες δεξιότητες. Τον περιορισμό αυτό έρχεται να καλύψει η ευελιξία που προσφέρει η τεχνολογία εικονικής πραγματικότητας (virtual reality, VR) (Bashiri και συν., 2017), η οποία αναλύεται στην επόμενη ενότητα.

**Εικονική Πραγματικότητα**

Η τεχνολογία εικονικής πραγματικότητας προσφέρει τη δυνατότητα προσομοίωσης μέσω του ηλεκτρονικού υπολογιστή μιας τρισδιάστατης εικόνας ή περιβάλλοντος με το οποίο μπορεί το άτομο να αλληλεπιδράσει μέσω πολλαπλών αισθητήριων καναλιών επιτρέποντάς του να συμπεριφερθεί όπως θα συμπεριφερόταν σε συνθήκες του πραγματικού κόσμου (Bashiri και συν., 2017. Shema-Shiratzky και συν., 2019. Wang & Reid, 2011). Η τεχνολογία αυτή χρησιμοποιείται εδώ και αρκετά χρόνια σε διάφορους τομείς όπως είναι η εκπαίδευση, η κατάρτιση, η ψυχαγωγία, ο στρατός, η ιατρική, η χειρουργική και η θεραπευτική παρέμβαση (Anton και συν., 2009. Bashiri και συν., 2017).

Αρκετές έρευνες έχουν δείξει ότι οι δυνατότητες της εικονικής πραγματικότητας στην παρέμβαση για παιδιά με δυσκολίες (π.χ. ΔΕΠ-Υ, αυτισμός, εγκεφαλική παράλυση κτλ.) περιλαμβάνουν την ευελιξία σε σχέση με τις απαιτήσεις του κάθε παιδιού, τη δυνατότητα ελέγχου από μέρους του θεραπευτή και προσαρμογής των στοιχείων της παρέμβασης με βάση το ατομικό επίπεδο του κάθε παιδιού, την αίσθηση της εμβύθισης από μέρους των παιδιών σε ένα περιβάλλον που είναι όμοιο με αυτό στο οποίο αναμένεται να μεταφερθούν οι μαθημένες δεξιότητες, καθώς και την εκμάθηση και εκπαίδευση των νέων δεξιοτήτων σε ένα ασφαλές περιβάλλον (Jeffs, 2010. Parsons και συν., 2017. Wang & Reid, 2011).

Όσον αφορά συγκεκριμένα στα παιδιά με διάγνωση ΔΕΠ-Υ φαίνεται ότι με τη χρήση της τεχνολογίας εικονικής πραγματικότητας στην παρέμβαση προσφέρονται εκτός από τα παραπάνω, και η δυνατότητα για εξατομίκευση των δραστηριοτήτων για το κάθε παιδί ή υποομάδα παιδιών ανάλογα με τις δυσκολίες που παρουσιάζει ενσωματώνοντας τα ενδιαφέροντα του κάθε παιδιού και τις προτιμήσεις του στο πρόγραμμα παρέμβασης (Bashiri και συν. 2017. Wang & Reid, 2011). Επομένως, μπορεί να τροποποιηθεί ένα πολύπλοκο εικονικό περιβάλλον αφαιρώντας αρκετά διασπαστικά στοιχεία με στόχο την ενίσχυση της προσοχής, ενώ παράλληλα μπορεί να μεγιστοποιηθεί και η επίδραση της ανατροφοδότησης, η οποία μπορεί να είναι άμεση και είναι πολύ σημαντική για τα παιδιά με ΔΕΠ-Υ.

Επιπρόσθετα, η χρήση της εικονικής πραγματικότητας στην παρέμβαση παρέχει τη δυνατότητα ελέγχου όσον αφορά στο σχεδιασμό του προγράμματος με την ένταξη δομημένων και συστηματικών εκπαιδευτικών στρατηγικών από τον θεραπευτή, ενώ η παρέμβαση γίνεται πιο διαδραστική και διασκεδαστική ενισχύοντας τα κίνητρα των παιδιών (Anton και συν., 2009. Bashiri και συν., 2017. Wang & Reid, 2011). Μέσα από την ανασκόπηση ερευνών που έκαναν η Bashiri και οι συνεργάτες της (2017) φάνηκε ότι η χρήση της εικονικής πραγματικότητας μειώνει το κόστος και το χρόνο της παρέμβασης, ενώ μπορεί να βελτιώσει τη μνήμη εργασίας, την επιτελική λειτουργικότητα και τις γνωστικές διαδικασίες (πχ. προσοχή).

Παράλληλα, σε μια πιλοτική έρευνα που έγινε από τη Shema-Shiratzky και τους συνεργάτες της (2019) σε δείγμα 14 παιδιών σχολικής ηλικίας με διάγνωση ΔΕΠ-Υ, στην οποία ήθελαν να εξετάσουν την αποτελεσματικότητα ενός συνδυασμού γνωστικής και κινητικής εκπαίδευσης με τη χρήση εικονικής πραγματικότητας για την ενίσχυση της συμπεριφορικής και γνωστικής λειτουργικότητας στα παιδιά, φάνηκε από τις αναφορές των γονέων ότι υπήρξε μια σημαντική βελτίωση στα κοινωνικά προβλήματα και τη ψυχοσωματική συμπεριφορά των παιδιών μετά την παρέμβαση. Επίσης, παρατηρήθηκε βελτίωση στην κατευθυνόμενη και επιλεκτική προσοχή, οι οποίες αντανακλούσαν καλύτερα σκορ επιτελικής λειτουργικότητας, αλλά και στη μνήμη εργασίας, παρόλο που ο δείκτης προσοχής (εστιασμένη προσοχή και επαγρύπνηση) παρέμεινε σταθερός (Shema-Shiratzky και συν., 2019).

Γενικότερα, η τεχνολογία εικονικής πραγματικότητας θα μπορούσε να ενταχθεί σε ένα πολυεπίπεδο πρόγραμμα παρέμβασης για παιδιά με ΔΕΠ-Υ, καθώς προσφέρει δυνατότητες που δεν μπορούν να παρέχουν τα παραδοσιακά είδη παρέμβασης, όπως είναι η υψηλή οικολογική εγκυρότητα που μπορεί να αυξήσει τις πιθανότητες μεταφοράς και γενίκευσης των νέων μαθημένων δεξιοτήτων στον πραγματικό κόσμο (Anton και συν., 2009. Bashiri και συν., 2017).

Με βάση όσα έχουν αναφερθεί πιο πάνω, φαίνεται ότι οι δυσκολίες προσοχής και υπερκινητικότητας που παρουσιάζει ένα σημαντικό ποσοστό του παιδικού πληθυσμού επηρεάζουν σε μεγάλο βαθμό διάφορους τομείς της σχολικής, κοινωνικής και οικογενειακής τους ζωής καθιστώντας απαραίτητη την παρέμβαση και τη στήριξη τόσο των ίδιων των παιδιών όσο και των γονιών τους (Corcoran και συν., 2017. Sjöwall και συν., 2013. Tarver και συν., 2014). Επιπλέον, φαίνεται ότι είναι σημαντικό να γίνεται ένας συνδυασμός παρεμβάσεων, οι οποίες να μπορούν να εξατομικεύονται ανάλογα με τις δυσκολίες που παρουσιάζει το κάθε παιδί. Παράλληλα, θεωρείται ουσιαστικής σημασίας η μελέτη και των στοιχείων εκείνων που διαφοροποιούν το κάθε άτομο (π.χ. γνωστικό προφίλ) και το οικογενειακό του περιβάλλον (π.χ. οικογενειακές σχέσεις), καθώς οι παράγοντες αυτοί διαμεσολαβούν για την έκβαση της παρέμβασης και παίζουν ρόλο στην εξατομίκευση των παρεμβάσεων που προτείνονται (Haack και συν., 2017. Hinshaw, 2007).

Επομένως, στόχος της παρούσας ερευνητικής προσπάθειας είναι η μελέτη των ρυθμιστικών παραγόντων που φαίνεται να παίζουν ρόλο στην έκβαση των παρεμβάσεων σε παιδιά με δυσκολίες προσοχής, όπως είναι το γνωστικό τους προφίλ και το οικογενειακό τους περιβάλλον. Έτσι, αναμένεται να δημιουργηθεί μια *πολυεπίπεδη παρεμβατική μελέτη διαμήκους σχεδιασμού (longitudinal intervention research) για παιδιά με δυσκολίες προσοχής ή/και υπερκινητικότητα*. Το παρεμβατικό αυτό πρόγραμμα θα περιλαμβάνει στοιχεία από εμπειρικά βασισμένες παρεμβάσεις που χρησιμοποιούνται για τα παιδιά με δυσκολίες προσοχής και υπερκινητικότητα σε συνδυασμό με τη χρήση εικονικής πραγματικότητας. Πιο συγκεκριμένα, αναμένεται να περιλαμβάνει: α) εκπαίδευση των παιδιών σε γνωστικές δεξιότητες και δεξιότητες συναισθηματικής αυτοδιαχείρισης βασισμένες σε αρχές της γνωστικο-συμπεριφορικής προσέγγισης και χρήση τεχνολογίας εικονικής πραγματικότητας, β) εκπαίδευση των γονέων σε βέλτιστες γονεϊκές πρακτικές και γ) στήριξη των γονέων με στόχο την ενίσχυση της συναισθηματικής αυτοδιαχείρισης και μείωση του στρες.

Οι υποθέσεις της προτεινόμενης ερευνητικής μελέτης είναι ότι:

1. Tα παιδιά γονέων που χαρακτηρίζονται από υποστηρικτικό γονεϊκό στυλ θα έχουν καλύτερα επίπεδα ψυχοκοινωνικής προσαρμογής σε σχέση με παιδιά γονέων που χαρακτηρίζονται από αυταρχικό ή αυστηρό γονεϊκό στυλ
2. Τα παιδιά της ομάδας παρέμβασης θα παρουσιάσουν βελτίωση όσον αφορά στα συμπεριφορικά προβλήματα που παρουσιάζουν, τις μετρήσεις στα έργα προσοχής και την ψυχοκοινωνική τους προσαρμογή σε σχέση με τα παιδιά της ομάδας ελέγχου μετά την παρέμβαση
3. Οι γονείς της ομάδας παρέμβασης θα παρουσιάσουν μείωση στις μετρήσεις γονεϊκού άγχους, ενώ θα παρουσιάσουν βελτίωση στο αίσθημα γονεϊκής αυτοαποτελεσματικότητας σε σχέση με τους γονείς της ομάδας ελέγχου μετά την παρέμβαση
4. Θα διερευνηθεί το γονεϊκό στυλ διαπαιδαγώγησης σε σχέση με τα αποτελέσματα της παρέμβασης προκειμένου να διαφανεί ποιο στυλ γονεϊκής διαπαιδαγώγησης βοηθά στην καλύτερη έκβαση της παρέμβασης
5. Θα διερευνηθούν τα χαρακτηριστικά και το γνωστικό προφίλ των παιδιών σε σχέση με τα αποτελέσματα της παρέμβασης προκειμένου να διαφανεί πώς αυτά μπορούν να παίξουν καθοριστικό ρόλο στην έκβαση ενός παρεμβατικού προγράμματος.

**Μεθοδολογία**

**Συμμετέχοντες**

Αναμένεται να ληφθούν δεδομένα από περίπου 80 παιδιά ηλικίας 9-12 ετών (Δ’-Στ’ τάξη Δημοτικού Σχολείου) που έχουν ήδη λάβει διάγνωση Διαταραχής Ελλειμματικής Προσοχής-Υπερκινητικότητας (40 παιδιά για την πειραματική ομάδα και αντίστοιχος αριθμός παιδιών για ομάδα ελέγχου). Οι συμμετέχοντες θα εντοπιστούν στα Κοινοτικά Κέντρα Ψυχικής Υγείας Παιδιών και Εφήβων (Κοι.Κε.Ψ.Υ.Π.Ε.) στο Ηράκλειο και στο Ρέθυμνο, καθώς και στο νομό Αττικής, αλλά και στα Κέντρα Εκπαιδευτικής και Συμβουλευτικής Υποστήριξης (Κ.Ε.Σ.Υ.) των νομών Ρεθύμνου, Ηρακλείου, Χανίων και του νομού Αττικής. Ο αριθμός αυτός του δείγματος θεωρείται κατάλληλος, καθώς μέσα από αναλύσεις που έγιναν, σε επίπεδο στατιστικής σημαντικότητας 5%, χρειάζονται περίπου 36 άτομα σε κάθε ομάδα για να επιτευχθεί στατιστική ισχύς 90% για απόρριψη της μηδενικής υπόθεσης ότι δεν υπάρχει διαφορά μεταξύ των δύο συνθηκών. Ο υπολογισμός αυτός έγινε για αναλύσεις επαναληπτικών μετρήσεων εντός και μεταξύ των ομάδων.

Η ερευνητική ομάδα αναμένεται να προσεγγίσει τους γονείς των παιδιών μέσα από διαδικασίες γνωστοποίησης της ερευνητικής προσπάθειας με τη συνεργασία και ειδικών που εργάζονται στα Κοι.Κε.Ψ.Υ.Π.Ε και Κ.Ε.Σ.Υ. προκειμένου να μάθουν για το σκοπό της συγκεκριμένης έρευνας. Θα δοθούν έντυπα ενημέρωσης και συγκατάθεσης, τα οποία οι γονείς των παιδιών που επιθυμούν να λάβουν μέρος στην έρευνα θα συμπληρώσουν και θα επιστρέψουν στην ερευνητική ομάδα. Κριτήρια επιλογής των παιδιών που θα λάβουν μέρος στην έρευνα θα αποτελέσουν η ηλικία, το φύλο (σε μια προσπάθεια να υπάρχει αναλογία αγοριών - κοριτσιών), η μη λήψη φαρμακευτικής αγωγής, η μη συμμετοχή σε άλλο πρόγραμμα παρέμβασης που βασίζεται στη γνωστικο-συμπεριφορική προσέγγιση και η μη ύπαρξη συννοσηρών διαταραχών. Κριτήριο αποκλεισμού από την έρευνα θα αποτελέσει η μη κάλυψη των κριτηρίων επιλογής.

Στη συνέχεια, τα παιδιά θα τοποθετηθούν στις δύο ομάδες (πειραματική ομάδα και ομάδα ελέγχου) με τυχαία τοποθέτηση, όπου η πειραματική ομάδα θα λάβει την παρέμβαση. Για δεοντολογικούς λόγους, οι γονείς της ομάδας ελέγχου θα λάβουν ψυχοεκπαίδευση σχετικά με τις δυσκολίες που παρουσιάζουν τα παιδιά τους με τη μορφή ενημερωτικής ημερίδας μετά το τέλος και της δεύτερης αξιολόγησης (αξιολόγηση μετά την παρέμβαση).

**Εργαλεία Έρευνας**

Τα εργαλεία που θα χρησιμοποιηθούν για τη λήψη των δεδομένων της παρούσας έρευνας έχουν χρησιμοποιηθεί ευρέως σε παρόμοιου είδους έρευνες με συμμετέχοντες παιδιά με δυσκολίες προσοχής και υπερκινητικότητα. Θα χρησιμοποιηθούν ερωτηματολόγια για λήψη ιστορικού και δημογραφικών στοιχείων από τους γονείς, καθώς επίσης και στοιχεία από τις αξιολογήσεις που έλαβαν τα παιδιά στα κέντρα ψυχικής υγείας μετά από σχετική συγκατάθεση που θα ληφθεί από τους γονείς/κηδεμόνες των παιδιών.

Επίσης, θα χρησιμοποιηθούν ερωτηματολόγια που αφορούν τους ίδιους τους γονείς, καθώς είναι σημαντικό να ληφθούν στοιχεία σχετικά με το γονεϊκό στυλ, την αντίληψη γονεϊκής αυτοαποτελεσματικότητας, αλλά και το άγχος που πιθανόν να βιώνουν, αφού αποτελούν παράγοντες που μπορούν να επηρεάσουν την έκβαση μιας παρέμβασης. Τα ερωτηματολόγια που αναμένεται να χρησιμοποιηθούν είναι:

1. Αυτοσχέδιο ερωτηματολόγιο δημογραφικών στοιχείων (βλ. Παράρτημα Α)
2. το Ερωτηματολόγιο Τυπολογίας Γονέων (για πατέρα και μητέρα) (προσαρμογή και στάθμιση του Parenting Styles and Dimensions Questionnaire από Μαριδάκη-Κασσωτάκη, 2009 και Αντωνοπούλου & Τσίτσας, 2011, βλ. Παράρτημα Α), το οποίο αποτελεί ένα ερωτηματολόγιο αυτο-αναφοράς σχετικά με τον τρόπο διαπαιδαγώγησης των παιδιών και αξιολογεί τον υποστηρικτικό γονέα, τον αυταρχικό γονέα, τον αυστηρό γονέα και τον επιτρεπτικό γονέα,
3. το Ερωτηματολόγιο Γονεϊκού Στρες-Σύντομη μορφή (Parenting Stress Index-Short form, Abidin, 1995, μετάφραση από Λεζέ, 2013, βλ. Παράρτημα Α), το οποίο μετρά το γονεϊκό άγχος ως μεταβλητή που επηρεάζεται από τα χαρακτηριστικά του παιδιού, του γονέα και των καταστάσεων που περιβάλλουν τη μεταξύ τους σχέση και βοηθά στο να παρατηρηθεί το επίπεδο γονεϊκού στρες, οι ελλιπείς μέθοδοι διαπαιδαγώγησης και η προσαρμοστικότητα του παιδιού στο οικογενειακό περιβάλλον,
4. η Κλίμακα Μέτρησης Γονεϊκής Αυτοαποτελεσματικότητας (Tool to Measure Parenting Self-Efficacy, TOPSE, Kendall & Bloomfield, 2005, μετάφραση και προσαρμογή από Καρακώστα-Στεφανοπούλου & Μαλικιώση-Λοϊζου, 2013, βλ. Παράρτημα Α), η οποία μετρά τις αντιλήψεις αυτοαποτελεσματικότητας των γονέων σε συγκεκριμένους τομείς άσκησης του γονεϊκού τους ρόλου, και
5. η Ελληνική Κλίμακα Αξιολόγησης της ΔΕΠ/Υ-IV (ADHD Rating Scale-IV: Home Version, DuPaul, Power, Anastopoulos, & Reid, 1998, προσαρμογή και στάθμιση στον ελληνικό πληθυσμό από Καλαντζή-Αζίζι, Αγγελή, & Ευσταθίου, 2006, βλ. Παράρτημα Α), η οποία αφορά στη συχνότητα των συμπτωμάτων της διαταραχής.

Παράλληλα με τις μετρήσεις που θα ληφθούν από τους γονείς, αναμένεται να ληφθούν μετρήσεις και από τα παιδιά που θα λάβουν μέρος στην ερευνητική αυτή προσπάθεια. Αρχικά, θα δοθούν ξεχωριστά έντυπα ενημέρωσης και συγκατάθεσης στα παιδιά σχετικά με τη συμμετοχή τους (βλ. Παράρτημα Β), καθώς και διαβεβαίωση ότι οι πληροφορίες που θα ληφθούν θα είναι εμπιστευτικές και ότι μπορούν να αποχωρήσουν οποιαδήποτε στιγμή το επιθυμούν. Για την αξιολόγηση των παιδιών θα χρησιμοποιηθούν:

1. Αυτοσχέδιο ερωτηματολόγιο δημογραφικών στοιχείων (βλ. Παράρτημα Α)
2. η Κλίμακα Αξιολόγησης της Νοημοσύνης του Wechsler (WISC-V), προκειμένου να ληφθούν πληροφορίες σχετικά με το γνωστικό τους προφίλ (ή συγκεκριμένες υποκλίμακες αυτής)
3. το Τεστ Ψυχοκοινωνικής Προσαρμογής (Χατζηχρήστου και συν., 2008, βλ. Παράρτημα Α), το οποίο αποτελεί μια κλίμακα αυτο-αναφοράς για παιδιά ηλικίας 10-12 ετών και δίνει πληροφορίες σχετικά με ορισμένα χαρακτηριστικά της ψυχοκοινωνικής προσαρμογής, όπως την αξιολογούν τα ίδια τα παιδιά, με στόχο τον εντοπισμό τόσο των δεξιοτήτων ή ελλειμμάτων στον κοινωνικό, συναισθηματικό τομέα και την κοινωνική τους προσαρμογή όσο και των δυσκολιών ενδοπροσωπικής προσαρμογής,
4. το Ερωτηματολόγιο για τις Αντιλήψεις των Παιδιών για την Τυπολογία των Γονέων (πατέρα και μητέρας) (Parenting Styles and Dimensions Questionnaire, βλ. Παράρτημα Α), προκειμένου να διαφανεί πώς τα παιδιά αντιλαμβάνονται το γονεϊκό στυλ διαπαιδαγώγησης και κατά πόσο συμφωνούν τα αποτελέσματα των αντιλήψεων των παιδιών με αυτά που θα ληφθούν από τους ίδιους τους γονείς, και
5. η Κλίμακα Μέτρησης της Αντίληψης της Γονεϊκής Αποδοχής/Απόρριψης-Συνοπτική Έκδοση (Parental Acceptance/Rejection-Short Form, PARQ-Child, Rohner 2002, μετάφραση και προσαρμογή από Τσαούση, Γιοβαζολιά & Μάσχα, 2012, βλ. Παράρτημα Α), προκειμένου να διαφανεί πώς τα ίδια τα παιδιά αντιλαμβάνονται την αποδοχή ή απόρριψη από τους γονείς τους.

Εκτός από τις παραπάνω μετρήσεις, αναμένεται να ληφθούν και μετρήσεις από γνωστικά τεστ στον υπολογιστή, τα οποία έχουν αποδείξει ερευνητικά τη σχέση τους με τις δυσκολίες που παρουσιάζονται στα παιδιά με δυσκολίες προσοχής και υπερκινητικότητα/ παρορμητικότητα. Οι μετρήσεις που θα ληφθούν αφορούν σε έργο τύπου Posner, το οποίο θα είναι προσαρμοσμένο για παιδιά, καθώς μετρά την προσοχή και την ικανότητα μετατόπισης της προσοχής και σε ένα έργο Continuous Performance Test (CPT), το οποίο μελετά την εστιασμένη και επιλεκτική προσοχή των ατόμων, καθώς αναμένεται από αυτά να αντιδρούν σε ένα στόχο ερέθισμα και να αγνοούν διασπαστικά και άσχετα ερεθίσματα.

Επιπλέον, κατά τη διάρκεια της εκτέλεσης των συγκεκριμένων έργων στον υπολογιστή αναμένεται να λαμβάνονται μετρήσεις οφθαλμικών κινήσεων με τη χρήση καταγραφέα οφθαλμοκίνησης (eye-tracker) που θα είναι ενσωματωμένος στην οθόνη του ηλεκτρονικού υπολογιστή. Ο στόχος είναι να ληφθούν και μετρήσεις σχετικά με την οφθαλμική προσήλωση των παιδιών και κατά πόσο μπορούν να ελέγξουν αντανακλαστικές/αυτόνομες οφθαλμικές κινήσεις σε διασπαστικά ερεθίσματα (π.χ. κατά τη διάρκεια του έργου CPT). Οι μετρήσεις με τα γνωστικά τεστ και τις οφθαλμικές κινήσεις χρειάζεται να ληφθούν προκειμένου να υπάρξει σύγκριση με αντίστοιχες μετρήσεις στο τέλος της παρέμβασης, για να διαφανεί κατά πόσο η παρέμβαση ήταν αποτελεσματική στη βελτίωση της επιλεκτικής και της εστιασμένης προσοχής στις οποίες τα παιδιά με ΔΕΠ-Υ φαίνεται να παρουσιάζουν ελλείμματα.

Όλες οι πιο πάνω μετρήσεις (ερωτηματολόγια γονέων, παιδιών και γνωστικά έργα) θεωρούνται απαραίτητες, καθώς θα δώσουν πληροφορίες που μπορούν να μελετηθούν σχετικά με πιθανούς καθοριστικούς παράγοντες για την έκβαση της παρέμβασης, ενώ θα δώσουν και στοιχεία από διαφορετικά επίπεδα ανάλυσης όπως αυτά καθορίζονται από την ερευνητική προσέγγιση RDoC.

**Διαδικασία Υλοποίησης της Έρευνας και του Παρεμβατικού Προγράμματος**

Αρχικά, θα γίνουν επαφές από την ερευνητική ομάδα με τα Κοι.Κε.Ψ.Υ.Π.Ε. και Κ.Ε.Σ.Υ. προκειμένου να ενημερωθούν οι ειδικοί για τη συγκεκριμένη ερευνητική προσπάθεια και να γίνουν οι απαραίτητες ενέργειες προσέλκυσης των γονέων παιδιών με διάγνωση ΔΕΠ-Υ, ώστε να ενημερωθούν για την έρευνα. Στη συνέχεια, θα δοθούν τα έντυπα ενημέρωσης και συγκατάθεσης στους γονείς (βλ. Παράρτημα Β), προκειμένου να ληφθεί ένας αριθμός συμμετεχόντων.

Πριν την έναρξη του προγράμματος παρέμβασης, θα πραγματοποιηθεί μια πιλοτική μελέτη με μικρό αριθμό συμμετεχόντων, προκειμένου να διερευνηθεί η σκοπιμότητα και η χρηστικότητα (feasibility and usability) της τεχνολογίας εικονικής πραγματικότητας, καθώς αποτελεί μια νέα μέθοδο που εισάγεται στην παρέμβαση. Μετά την πιλοτική αυτή μελέτη θα γίνουν οι απαραίτητες αλλαγές με βάση τις δηλώσεις των παιδιών σχετικά με τη χρήση του εξοπλισμού και την εικονική πραγματικότητα ως μέρος της παρέμβασης.

Έπειτα, θα ακολουθήσει η επιλογή των παιδιών που θα αποτελέσουν κατάλληλο αριθμό δείγματος με βάση τα κριτήρια επιλογής (ηλικία, φύλο, μη λήψη φαρμακευτικής αγωγής ή συμμετοχής σε άλλου είδους θεραπευτική παρέμβαση γνωστικο-συμπεριφορικής προσέγγισης, μη ύπαρξη συννοσηρών διαταραχών). Το δείγμα συμμετεχόντων που θα επιλεγεί θα χωριστεί τυχαία σε δύο ομάδες (ομάδα παρέμβασης και ομάδα ελέγχου).

Μετά την επιλογή και την κατανομή των συμμετεχόντων στις ομάδες θα κληθούν για να ενημερωθούν σχετικά με την επιλογή τους στην έρευνα. Θα ακολουθήσουν δύο συναντήσεις αξιολόγησης, όπου θα δοθούν στους γονείς τα ερωτηματολόγια που έχουν περιγραφεί πιο πάνω προκειμένου να τα συμπληρώσουν (αξιολόγηση γονέων πριν την παρέμβαση, T1). Πριν ξεκινήσει η αξιολόγηση των παιδιών, αυτά θα ενημερωθούν για το σκοπό της έρευνας μέσα από ξεχωριστό έντυπο ενημέρωσης και συγκατάθεσης που θα συμπληρώσουν (βλ. Παράρτημα Β). Εφόσον ληφθεί η συγκατάθεσή τους, τα παιδιά θα αξιολογηθούν με τα έργα που έχουν επίσης περιγραφεί πιο πάνω (αξιολόγηση παιδιών πριν την παρέμβαση, T1).

Στη συνέχεια, η ομάδα των γονέων των παιδιών που θα αποτελέσουν την ομάδα παρέμβασης αναμένεται να λάβει μέρος σε πρόγραμμα ψυχοεκπαίδευσης διάρκειας 8-10 εβδομαδιαίων συναντήσεων σχετικά με μεθόδους τροποποίησης της συμπεριφοράς, εκπαίδευση σε βέλτιστες γονεϊκές πρακτικές και σε τεχνικές ενίσχυσης της ενσυνειδητότητας με στόχο τη μείωση του στρες που αναμένεται ότι βιώνουν και την ενίσχυση της συναισθηματικής αυτοδιαχείρισης.

Όσον αφορά στο περιεχόμενο του ψυχοεκπαιδευτικού προγράμματος αναμένεται ότι θα περιλαμβάνει μια αρχική συνάντηση, όπου θα δοθούν γενικές πληροφορίες σχετικά με τις δυσκολίες προσοχής που βιώνουν τα παιδιά, καθώς και πληροφορίες από τους ίδιους τους γονείς για το πώς βιώνουν τις δυσκολίες αυτές, ενώ θα δοθούν στοιχεία για αντικατάσταση πιθανών λανθασμένων αντιλήψεων που μπορεί να έχουν και θα διασφαλιστεί η εμπιστευτικότητα από τους ίδιους προς την ομάδα και τις συναντήσεις. Έπειτα, αναμένεται ότι στις επόμενες συναντήσεις, θα εκπαιδευτούν σε διάφορες συμπεριφορικές τεχνικές (π.χ. θετική προσοχή σε θετικές συμπεριφορές που παρουσιάζει το παιδί, θέσπιση οικογενειακών κανόνων, πώς να δίνουν οδηγίες, θετική ενίσχυση για την εκδήλωση προσαρμοστικών συμπεριφορών, σύστημα ανταλλάξιμων αμοιβών, γενίκευση των τεχνικών και σε πλαίσια εκτός σπιτιού κτλ.) και τεχνικές ενίσχυσης της ενσυνειδητότητας. Παράλληλα, αναμένεται ότι κατά τη διάρκεια των συναντήσεων, οι γονείς θα εκδηλώνουν τις ανησυχίες τους σχετικά με την εκπαίδευση, αλλά και με συμπεριφορές που εμφανίζουν τα παιδιά τους και θα δίνεται καθοδήγηση για τροποποίηση τεχνικών με βάση τις ανάγκες που αυτοί θα εκδηλώνουν.

Όσον αφορά στο πρόγραμμα παρέμβασης των παιδιών, αυτό αναμένεται ότι θα αποτελείται από 18-20 εβδομαδιαίες συναντήσεις (1 ώρα/συνάντηση) και θα περιλαμβάνει εκπαίδευση των παιδιών σε γνωστικές δεξιότητες και δεξιότητες συναισθηματικής αυτοδιαχείρισης βασισμένες σε αρχές της γνωστικο-συμπεριφορικής προσέγγισης. Παράλληλα, θα χρησιμοποιηθεί και η τεχνολογία εικονικής πραγματικότητας στην παρέμβαση, καθώς προσφέρει τη δυνατότητα για εκπαίδευση σε προσομοιωμένα με τον πραγματικό κόσμο περιβάλλοντα. Ουσιαστικά, αναμένεται ότι τα παιδιά θα εκπαιδευτούν σε τεχνικές αυτοαξιολόγησης, αυτοενίσχυσης και αυτορρύθμισης της συμπεριφοράς και των συναισθημάτων τους. Κατά τη διάρκεια των συναντήσεων, αναμένεται ότι μέσω της τεχνολογίας εικονικής πραγματικότητας τα παιδιά θα εκτελούν διάφορα έργα των οποίων η δυσκολία και το περιεχόμενο θα μεταβάλλονται, ενώ θα εκπαιδεύονται σε δεξιότητες διατήρησης και μετατόπισης της προσοχής και θα υπάρχει συνδυασμός με τις τεχνικές που θα μαθαίνουν οι γονείς (π.χ. θα ενισχύονται με πόντους στα παιχνίδια τους οποίους θα μπορούν να ανταλλάξουν με αμοιβές που θα έχουν καταρτιστεί στο πρόγραμμα αμοιβών με τους γονείς τους).

Όταν ολοκληρωθούν οι συναντήσεις με τα παιδιά, θα γίνει μια εκ νέου συνάντηση με τους γονείς προκειμένου να συζητηθούν τυχόν δυσκολίες που προέκυψαν από το τέλος της ψυχοεκπαίδευσης μέχρι και τη συγκεκριμένη συνάντηση και θα γίνει μια ανασκόπηση των τεχνικών και στρατηγικών στις οποίες εκπαιδεύτηκαν. Επίσης, θα τους χορηγηθούν ξανά τα ερωτηματολόγια που θα έχουν συμπληρώσει κατά την αρχική αξιολόγηση προκειμένου να φανεί κατά πόσο έχουν παρατηρηθεί αλλαγές μετά την παρέμβαση (αξιολόγηση γονέων μετά την παρέμβαση, T2). Επιπλέον, αναμένεται να γίνει εκ νέου αξιολόγηση των παιδιών (αξιολόγηση παιδιών μετά την παρέμβαση, T2) με τα έργα που θα χρησιμοποιηθούν κατά την αρχική τους αξιολόγηση με στόχο πάλι να ελεγχθεί κατά πόσο έχουν επέλθει αλλαγές ως αποτέλεσμα της παρέμβασης στην ψυχοκοινωνική τους προσαρμογή, στον τρόπο που αντιλαμβάνονται την αποδοχή ή απόρριψη από τους γονείς τους και στο κατά πόσο αντιλαμβάνονται αλλαγές στον τρόπο διαπαιδαγώγησης που χρησιμοποιούν οι γονείς τους. Παράλληλα, θα ληφθούν μετρήσεις από τα γνωστικά έργα στον ηλεκτρονικό υπολογιστή, για να μελετηθεί αν υπάρχουν αλλαγές στα αποτελέσματά τους κατά τη διάρκεια των οποίων θα λαμβάνονται και πάλι μετρήσεις οφθαλμικών κινήσεων.

Στη συνέχεια, η ερευνητική ομάδα θα επικοινωνήσει ξανά με τους γονείς και τα παιδιά μετά από περίπου τρεις (3) μήνες για να διεξαχθεί μια τελική αξιολόγηση, όπου θα δοθούν τα ερωτηματολόγια εκ νέου και θα αξιολογηθούν τα παιδιά στα γνωστικά έργα (follow-up αξιολόγηση – μετρήσεις παρακολούθησης, T3) για να μελετηθεί κατά πόσο οι όποιες αλλαγές προέκυψαν από την παρέμβαση μπόρεσαν να διατηρηθούν και στο χρόνο μετά την παρέμβαση.

Όσον αφορά στους γονείς και τα παιδιά της ομάδας ελέγχου, αναμένεται ότι η αξιολόγηση θα περιλαμβάνει τα ίδια ερωτηματολόγια για τους γονείς και τα παιδιά και τα ίδια γνωστικά έργα στον υπολογιστή για τα παιδιά κατά τις ίδιες χρονικές στιγμές με την ομάδα παρέμβασης. Οι γονείς της συγκεκριμένης ομάδας, για δεοντολογικούς λόγους, θα λάβουν ψυχοεκπαίδευση σχετικά με τις δυσκολίες που παρουσιάζουν τα παιδιά τους και τεχνικές τροποποίησης της συμπεριφοράς με τη μορφή ενημερωτικής ημερίδας μετά τη δεύτερη συνάντηση αξιολόγησης (αξιολόγηση μετά την παρέμβαση, Τ2), όταν δηλαδή ολοκληρωθεί το παρεμβατικό πρόγραμμα για την πειραματική ομάδα.

**Αναμενόμενα Αποτελέσματα - Χρησιμότητα Έρευνας**

Μέσα από τη συγκεκριμένη ερευνητική προσπάθεια αναμένεται ουσιαστικά να ερευνηθούν οι παράγοντες εκείνοι που πρέπει να λαμβάνονται υπόψη κατά τη διάρκεια της δημιουργίας και εφαρμογής ενός προγράμματος παρέμβασης για παιδιά με δυσκολίες προσοχής. Οι μετρήσεις που θα ληφθούν από τα διάφορα ερωτηματολόγια και τα γνωστικά έργα θα παρέχουν πληροφορίες σχετικά με το γνωστικό προφίλ και τα χαρακτηριστικά των παιδιών, αλλά και στοιχεία σε σχέση με το οικογενειακό δυναμικό (σχέσεις στην οικογένεια, γονεϊκές πρακτικές διαπαιδαγώγησης, γονεϊκό στρες). Επίσης, θα αξιοποιηθούν σε σχέση με τα αποτελέσματα της παρέμβασης για να φανεί κατά πόσο αποτελούν ρυθμιστικούς παράγοντες στην έκβαση μιας παρέμβασης. Θα πραγματοποιηθούν αναλύσεις συνδιακύμανσης επαναληπτικών μετρήσεων, συγκρίσεις μεταξύ των ομάδων, καθώς και αναλύσεις , οι οποίες χρησιμοποιούνται για την εξέταση μεταβλητών που μπορεί να επιδρούν ρυθμιστικά στην έκβαση μιας παρέμβασης..

Επιπλέον, μέσα από τη συγκεκριμένη ερευνητική προσπάθεια αναμένεται να δημιουργηθεί ένα πολυεπίπεδο παρεμβατικό πρόγραμμα, το οποίο θα περιλαμβάνει εκπαίδευση τόσο των γονέων όσο και των παιδιών με στόχο τη βελτίωση των γνωστικών και συμπεριφορικών δεξιοτήτων των παιδιών.

Ένα σημαντικό στοιχείο στο οποίο καινοτομεί και το οποίο διαφοροποιεί τη συγκεκριμένη προτεινόμενη έρευνα από άλλες έρευνες με παρεμβατικά προγράμματα είναι η ένταξη της τεχνολογίας εικονικής πραγματικότητας, η οποία τα τελευταία χρόνια χρησιμοποιείται σε διάφορους τομείς. Η τεχνολογία εικονικής πραγματικότητας, όπως αναφέρθηκε πιο πάνω, προσφέρει δυνατότητες που δεν προσφέρουν τα παραδοσιακά είδη παρέμβασης ενισχύοντας την πιθανότητα για μεταφορά και γενίκευση των μαθημένων δεξιοτήτων και σε άλλα περιβάλλοντα εκτός από το περιβάλλον παρέμβασης, ενώ παρέχει ένα πιο διασκεδαστικό παρεμβατικό περιβάλλον ενισχύοντας τα κίνητρα των παιδιών.

Ουσιαστικά, η μελέτη των παραγόντων εκείνων που φαίνεται να συμβάλλουν ή όχι στην αποτελεσματικότητα ενός παρεμβατικού προγράμματος θα προσφέρει μια πιο ολοκληρωμένη εικόνα όσον αφορά στην ανάγκη για εμπειρικά βασισμένες και εξατομικευμένες παρεμβάσεις για τα παιδιά με βάση τις ανάγκες του κάθε παιδιού στοχεύοντας στην πρόληψη της αρνητικής εξελικτικής τους πορείας (π.χ. σχολική αποτυχία, μακροπρόθεσμες δυσκολίες προσαρμογής, δυσκολίες στις κοινωνικές και ερωτικές σχέσεις, προβλήματα με το νόμο κτλ.).

Τα οφέλη που θα προκύψουν από την υλοποίηση της συγκεκριμένης ερευνητικής προσπάθειας είναι σημαντικά κυρίως για τους μαθητές με νευροαναπτυξιακές διαταραχές όπως η ΔΕΠ-Υ και τις οικογένειές τους, καθώς τα αποτελέσματα θα συμβάλουν στην καλύτερη κατανόηση των ψυχοσυναισθηματικών και μαθησιακών επιπτώσεων στα παιδιά αυτά και στο οικογενειακό τους δυναμικό, ενώ η ανάπτυξη του παρεμβατικού προγράμματος για την καλύτερη προσαρμογή και ανάπτυξή τους θα μπορούσε να αποτελέσει μια εμπειρικά τεκμηριωμένη αποτελεσματική πρακτική που θα μπορούσε να εφαρμοστεί ευρύτερα σε πληθυσμούς με παρόμοια χαρακτηριστικά.

**Θέματα Ηθικής και Δεοντολογίας**

**Συναίνεση Κατόπιν Ενημέρωσης για Συμμετοχή**

Οι γονείς και τα παιδιά που αναμένεται να συμμετέχουν στην προτεινόμενη έρευνα θα λάβουν σχετική ενημέρωση για τους σκοπούς της έρευνας και τη διαδικασία που θα ακολουθηθεί μέσα από τα έντυπα ενημέρωσης και συγκατάθεσης που θα τους δοθούν, αλλά και από προσωπική τους επικοινωνία με τους ερευνητές εάν επιθυμούν να λάβουν περαιτέρω πληροφορίες για την έρευνα. Οι συμμετέχοντες του δείγματος θα ενημερωθούν ότι μπορούν να αποσύρουν οποιαδήποτε στιγμή το επιθυμούν τη συμμετοχή τους από την έρευνα και τα δεδομένα που ίσως συλλέχθηκαν μέχρι τη στιγμή της απόσυρσής τους θα καταστραφούν.

**Προστασία Προσωπικών Δεδομένων**

Οποιαδήποτε πληροφορία αφορά στους συμμετέχοντες κατά τη διάρκεια της προτεινόμενης έρευνας και παρέμβασης θα παραμένει απόρρητη και οποιαδήποτε δημοσίευση προκύψει από τη συγκεκριμένη μελέτη θα παρουσιάσει τα ευρήματα ανώνυμα. Τα δεδομένα που θα ληφθούν από τους συμμετέχοντες του δείγματος (γονείς και παιδιά) θα χρησιμοποιηθούν αποκλειστικά για ερευνητικούς σκοπούς στο πλαίσιο της εκπόνησης της διδακτορικής διατριβής. Η συλλογή και η επεξεργασία των προσωπικών δεδομένων των συμμετεχόντων θα συμμορφώνεται πλήρως με τις απαιτήσεις του Γενικού Νόμου Προστασίας Δεδομένων 679/2016 της Ευρωπαϊκής Ένωσης.

Τόσο τα ερευνητικά δεδομένα που θα συλλεχθούν κατά τη διάρκεια της προτεινόμενης ερευνητικής μελέτης όσο και το υλικό που θα προκύψει από την εφαρμογή του παρεμβατικού προγράμματος για κάθε άτομο που θα συμμετάσχει θα κωδικοποιηθούν με αριθμούς και επομένως, τα προσωπικά στοιχεία των συμμετεχόντων σε σχέση με τα δεδομένα τους δεν θα εμφανίζονται πουθενά και δεν θα μπορούν να ταυτοποιηθούν οι μαθητές και οι γονείς τους. Επιπλέον, τυχόν προσωπικά στοιχεία που θα χρειαστεί να ληφθούν για την διεξαγωγή της έρευνας (π.χ. τηλέφωνο επικοινωνίας) θα φυλάσσονται κάτω από την ευθύνη της ερευνητικής ομάδας και κυρίως της Επιστημονικής Υπεύθυνης και θα βρίσκονται κλειδωμένα σε ασφαλή χώρο (στο *Εργαστήριο Εφαρμοσμένης Ψυχολογίας του Κέντρου Ερευνών και Μελετών του Πανεπιστημίου Κρήτης*). Πρόσβαση στα στοιχεία αυτά και τα δεδομένα που θα προκύψουν θα έχει μόνο το ερευνητικό προσωπικό της μελέτης, το οποίο δεσμεύεται για το απόρρητο των στοιχείων των συμμετεχόντων που θα συμμετάσχουν για τις ανάγκες της προτεινόμενης διδακτορικής διατριβής.

Οι ερευνητές, επίσης, δεσμεύονται να τηρήσουν αυστηρά την εμπιστευτικότητα και τους νόμους για την προστασία των δεδομένων ακολουθώντας τις σχετικές οδηγίες όπως ορίζονται από τη νομοθεσία τόσο της Ελλάδος όσο και της Ευρωπαϊκής Ένωσης και από την Αρχή Προστασίας Δεδομένων Προσωπικού Χαρακτήρα (ΑΠΔΠΧ).

Με την ολοκλήρωση της έρευνας και σε συνεννόηση τόσο με τον Επιστημονικό Υπεύθυνο όλου του προγράμματος (Καθηγητή Τ. Παπαδόπουλο, Πανεπιστήμιο Κύπρου, Τμήμα Ψυχολογίας) όσο και με την Υπεύθυνη Επεξεργασίας Προσωπικών Δεδομένων (Data Protection Officer, DPO) του Πανεπιστημίου Κρήτης κα. Έλλη Βενεδίκτου θα οριστεί ο τρόπος βάσει ευρωπαϊκών οδηγιών για την τελική διαχείριση και καταστροφή των δεδομένων.

**Παράπονα ή Καταγγελίες**

Τυχόν παράπονα ή καταγγελίες που θα προκύψουν από τους συμμετέχοντες θα μπορούν να υποβληθούν προφορικά ή γραπτά στην υποψήφια διδάκτορα, καθώς και μέσω τηλεφώνου ή ηλεκτρονικού ταχυδρομείου σε έναν από τους κύριους υπεύθυνους της έρευνας (Δρ. Δημητροπούλου, Δρ. Τσαούση). Τα στοιχεία τόσο της υποψήφιας διδάκτορας όσο και των κύριων υπεύθυνων της έρευνας θα βρίσκονται στα έντυπα ενημέρωσης και συγκατάθεσης τα οποία θα δοθούν στους συμμετέχοντες σε δύο αντίτυπα (ένα για επιστροφή στην ερευνητική ομάδα και ένα για δική τους χρήση).

Επίσης, για οποιαδήποτε παράπονα ή καταγγελίες σχετικά με τη διεξαγωγή της έρευνας, οι συμμετέχοντες θα μπορούν να προσφύγουν στην Επιτροπή Ηθικής και Δεοντολογίας του Πανεπιστημίου Κρήτης στα στοιχεία που θα αναγράφονται στο έντυπο ενημέρωσης και συγκατάθεσης. Όσον αφορά σε καταγγελία σχετικά με τη διαχείριση των προσωπικών τους δεδομένων θα μπορούν να απευθυνθούν στην DPO του Πανεπιστημίου Κρήτης και σε κάθε περίπτωση στην ΑΠΔΠΧ.

Βιβλιογραφία

American Psychiatric Association. (2013). *Diagnostic and statistical manual of mental disorders* (5th ed.). <https://doi.org/10.1176/appi.books.9780890425596>

Anton, R., Orpis, D., Dobrean, A., & David, D. (2009). Virtual reality in the rehabilitation of attention deficit/ hyperactivity disorder. Instrument construction principles. *Journal of Cognitive and Behavioral Psychotherapies*, *9*(2), 235-246. <http://search.ebscohost.com/login.aspx?direct=true&db=asn&AN=44483007&site=ehost-live>

Bashiri, A., Ghazisaeedi, M., & Shahmoradi, L. (2017). The opportunities of virtual reality in the rehabilitation of children with attention deficit hyperactivity disorder: a literature review. *Korean Journal of Pediatrics, 60*(11), 337-343. <https://doi.org/10.3345/kjp.2017.60.11.337>

Brassett-Harknett, A., & Butler, N. (2007). Attention-deficit/hyperactivity disorder: an overview of the etiology and a review of the literature relating to the correlates and lifecourse outcomes for men and women. *Clinical Psychology Review*, *27*(2), 188-210. <https://doi.org/10.1016/j.cpr.2005.06.001>

Corcoran, J., Schildt, B., Hochbrueckner, R., & Abell, J. (2017). Parents of children with attention deficit/hyperactivity disorder: A meta-synthesis, part I. *Child and Adolescent Social Work Journal*, *34*(4), 281-335. <https://doi.org/10.1007/s10560-016-0465-1>

Cuthbert, B. N. (2015). Research Domain Criteria: toward future psychiatric nosologies. *Dialogues in Clinical Neuroscience, 17*(1), 89-97.

Evans, S. W., Owens, J. S., & Bunford, N. (2014). Evidence-based psychosocial treatments for children and adolescents with attention-deficit/hyperactivity disorder. *Journal of Clinical Child & Adolescent Psychology*, *43*(4), 527-551. <https://doi.org/10.1080/15374416.2013.850700>

Flores, G. W. R., & Parra, V. A. B. (2014). Cognitive behavioral treatment in children with attention deficit hyperactivity disorder. *Revista de Psicología:(Universidad de Antioquía)*, *6*(2), 79-94.

Franke, B., Michelini, G., Asherson, P., Banaschewski, T., Bilbow, A., Buitelaar, J. K., Cormand, B., Faraone, S. V., Ginsberg, Y., Haavik, J., Kuntsi, J., Larsson, H., Lesch, K-P., Ramos-Quiroga, J. A., Rethelyi, J. M., Ribases, M., & Reif, A. (2018). Live fast, die young? A review on the developmental trajectories of ADHD across the lifespan. *European Neuropsychopharmacology*, *28*(10), 1059-1088. <https://doi.org/10.1016/j.euroneuro.2018.08.001>

Garvey, M., Avenevoli, S., & Anderson, K. (2016). The national institute of mental health research domain criteria and clinical research in child and adolescent psychiatry. *Journal of the American Academy of Child & Adolescent Psychiatry*, *55*(2), 93-98. <https://psycnet.apa.org/doi/10.1016/j.jaac.2015.11.002>

Haack, L. M., Villodas, M., McBurnett, K., Hinshaw, S., & Pfiffner, L. J. (2017). Parenting as a mechanism of change in psychosocial treatment for youth with ADHD, predominantly inattentive presentation. *Journal of Abnormal Child Psychology*, *45*(5), 841-855. <https://doi.org/10.1007/s10802-016-0199-8>

Han, Z. R., Ahemaitijiang, N., Yan, J., Hu, X., Parent, J., Dale, C., DiMarzio, K., & Singh, N. N. (2019). Parent mindfulness, parenting, and child psychopathology in China. *Mindfulness*, 1-10. <https://doi.org/10.1007/s12671-019-01111-z>

Hinshaw, S. P. (2007). Moderators and mediators of treatment outcome for youth with ADHD: Understanding for whom and how interventions work. *Journal of Pediatric Psychology*, *32*(6), 664-675. <https://doi.org/10.1093/jpepsy/jsl055>

Jeffs, T. L. (2010). Virtual reality and special needs. *Themes in Science and Technology Education*, *2*(1-2), 253-268.

Johnston, C., & Park, J. L. (2015). Interventions for attention-deficit hyperactivity disorder: a year in review. *Current Developmental Disorders Reports*, *2*(1), 38-45. <https://doi.org/10.1007/s40474-014-0034-2>

Lee, P. C., Niew, W. I., Yang, H. J., Chen, V. C. H., & Lin, K. C. (2012). A meta-analysis of behavioral parent training for children with attention deficit hyperactivity disorder. *Research in Developmental Disabilities*, *33*(6), 2040-2049. <https://doi.org/10.1016/j.ridd.2012.05.011>

Lifford, K. J., Harold, G. T., & Thapar, A. (2008). Parent–child relationships and ADHD symptoms: a longitudinal analysis. *Journal of Abnormal Child Psychology*, *36*(2), 285-296. <https://doi.org/10.1007/s10802-007-9177-5>

Luna, B., Velanova, K., & Geier, C. F. (2008). Development of eye-movement control. *Brain and Cognition, 68*(3), 293-308. <https://doi.org/10.1016/j.bandc.2008.08.019>

Maniadaki, K. (2019). Attention deficit/hyperactivity disorder: A real disorder throughout the lifespan. *JSM Pediatrics and Child Health, 4*(3), 1-3.

Μανιαδάκη, Κ., & Κάκουρος, Ε. (2016). *Η διαχείριση της ΔΕΠ-Υ. Από τη θεωρία στην πράξη*. Gutenberg.

Miller, C., & Brooker, B. (2017). Mindful programming for parents and teachers of children with ADHD. *Complimentary Therapies in Clinical Practice*, *28*, 108-115. <https://doi.org/10.1016/j.ctcp.2017.05.015>

Musser, E. D., & Raiker Jr, J. S. (2019). Attention-deficit/hyperactivity disorder: An integrated developmental psychopathology and Research Domain Criteria (RDoC) approach. *Comprehensive Psychiatry*, *90*, 65-72. <https://doi.org/10.1016/j.comppsych.2018.12.016>

|  |
| --- |

Aήνα: )﷽δολογικο Ακα εμφάνισηςικ2000Palili, A., Kolaitis, G., Vassi, I., Veltsista, A., Bakoula, C., & Gika, A. (2011). Inattention, hyperactivity, impulsivity—epidemiology and correlations: A nationwide greek study from birth to 18 years. *Journal of Child Neurology*, *26*(2), 199-204. [https://doi.org/10.1177/0883073810379640](https://psycnet.apa.org/doi/10.1177/0883073810379640)

Parent, J., McKee, L. G., Rough, J. N., & Forehand, R. (2016). The association of parent mindfulness with parenting and youth psychopathology across three developmental stages. *Journal of Abnormal Child Psychology*, *44*(1), 191-202. <https://doi.org/10.1007/s10802-015-9978-x>

Parsons, T. D., Riva, G., Parsons, S., Mantovani, F., Newbutt, N., Lin, L., Venturini, E., & Hall, T. (2017). Virtual reality in pediatric psychology. *Pediatrics*, *140*(Supplement 2), S86-S91. <https://doi.org/10.1542/peds.2016-1758I>

Pfiffner, L. J., & Haack, L. M. (2014). Behavior management for school-aged children with ADHD. *Child and Adolescent Psychiatric Clinics*, *23*(4), 731-746. <https://doi.org/10.1016/j.chc.2014.05.014>

Polanczyk, G., De Lima, M. S., Horta, B. L., Biederman, J., & Rohde, L. A. (2007). The worldwide prevalence of ADHD: A systematic review and metaregression analysis. *American Journal of Psychiatry*, *164*(6), 942-948. <https://ajp.psychiatryonline.org/doi/10.1176/ajp.2007.164.6.942>

Rajeh, A., Amanullah, S., Shivakumar, K., & Cole, J. (2017). Interventions in ADHD: A comparative review of stimulant medications and behavioral therapies. *Asian Journal of Psychiatry*, *25*, 131-135. <http://dx.doi.org/10.1016/j.ajp.2016.09.005>

Rommelse, N. N. J., Van der Stigchel, S., & Sergeant, J. A. (2008). A review on eye movement studies in childhood and adolescent psychiatry. *Brain and Cognition, 68*(3), 391-414. <https://doi.org/10.1016/j.bandc.2008.08.025>

Schellack, N., Meyer, J. C., & Chigome, A. K. (2019). The management of attention-deficit hyperactivity disorder in children: Updated 2019. *South African Pharmaceutical Journal*, *86*(5), 17-27. <http://sapj.co.za/index.php/SAPJ/article/view/2756>

Sekaninova, N., Mestanik, M., Mestanikova, A., Hamrakova, A., & Tonhajzerova, I. (2019). Novel approach to evaluate central autonomic regulation in attention deficit/hyperactivity disorder (ADHD). *Physiological Research*, *68*, 531-545. <https://doi.org/10.33549/physiolres.934160>

Sjöwall, D., Roth, L., Lindqvist, S., & Thorell, L. B. (2013). Multiple deficits in ADHD: executive dysfunction, delay aversion, reaction time variability, and emotional deficits. *Journal of Child Psychology and Psychiatry*, *54*(6), 619-627. <https://doi.org/10.1111/jcpp.12006>

Tarver, J., Daley, D., & Sayal, K. (2014). Attention‐deficit hyperactivity disorder (ADHD): an updated review of the essential facts. *Child: Care, Health and Development*, *40*(6), 762-774. <https://doi.org/10.1111/cch.12139>

Theule, J., Wiener, J., Tannock, R., & Jenkins, J. M. (2013). Parenting stress in families of children with ADHD: A meta-analysis. *Journal of Emotional and Behavioral Disorders*, *21*(1), 3-17. <https://doi.org/10.1177%2F1063426610387433>

Wang, M., & Reid, D. (2011). Virtual reality in pediatric neurorehabilitation: attention deficit hyperactivity disorder, autism and cerebral palsy. *Neuroepidemiology*, *36*(1), 2-18. <https://doi.org/10.1159/000320847>

Zachor, D., Hodgens, B., & Patterson, C. (2009). Treatment of attention-deficit/hyperactivity disorder (ADHD). In J.L.Matson, F. Andrasik, & M.L. Matson(eds.), *Treating childhood psychopathology and developmental disabilities* (pp.139-181). Springer. <https://doi.org/10.1007/978-0-387-09530-1_6>

**ΠΑΡΑΡΤΗΜΑ Α**

**Ερωτηματολόγια Γονέων:**

1. Δημογραφικά Στοιχεία
2. Ερωτηματολόγιο Τυπολογίας του Έλληνα Πατέρα
3. Ερωτηματολόγιο Τυπολογίας της Ελληνίδας Μητέρας
4. Ερωτηματολόγιο Γονεϊκού Στρες-Σύντομη μορφή (Parenting Stress Index-Short form)
5. Κλίμακα Μέτρησης Γονεϊκής Αυτοαποτελεσματικότητας (Tool to Measure Parenting Self-Efficacy, TOPSE)
6. Ελληνική Κλίμακα Αξιολόγησης της ΔΕΠ/Υ-IV

**Ερωτηματολόγια Παιδιών:**

1. Δημογραφικά Στοιχεία
2. Τεστ Ψυχοκοινωνικής Προσαρμογής
3. Ερωτηματολόγιο για τις Αντιλήψεις των Παιδιών για την Τυπολογία του Πατέρα
4. Ερωτηματολόγιο για τις Αντιλήψεις των Παιδιών για την Τυπολογία της Μητέρας
5. Κλίμακα Μέτρησης της Αντίληψης της Γονεϊκής Αποδοχής/Απόρριψης-Συνοπτική Έκδοση (Parental Acceptance/Rejection-Short Form, PARQ-Child) για πατέρα και μητέρα

**ΠΑΡΑΡΤΗΜΑ Β**

1. Έντυπο Συγκατάθεσης Γονέων Πειραματικής Ομάδας
2. Έντυπο Συγκατάθεσης Μαθητών Πειραματικής Ομάδας
3. Έντυπο Συγκατάθεσης Γονέων Ομάδας Ελέγχου
4. Έντυπο Συγκατάθεσης Μαθητών Ομάδας Ελέγχου

**ΠΑΡΑΡΤΗΜΑ Γ**

1. Χρηματοδότηση Έργου μέσα από το πρόγραμμα «Neo-PRISM-C: Neurodevelopmental Optimal-Predictors, Risk factors, and Intervention from a Systems approach to Maladjustment in Children», το οποίο αποτελεί εγκεκριμένη χρηματοδότηση από την Ευρωπαϊκή Ένωση στο πλαίσιο προγράμματος Horizon2020 - Marie Skłodowska-Curie Innovative Training Networks.


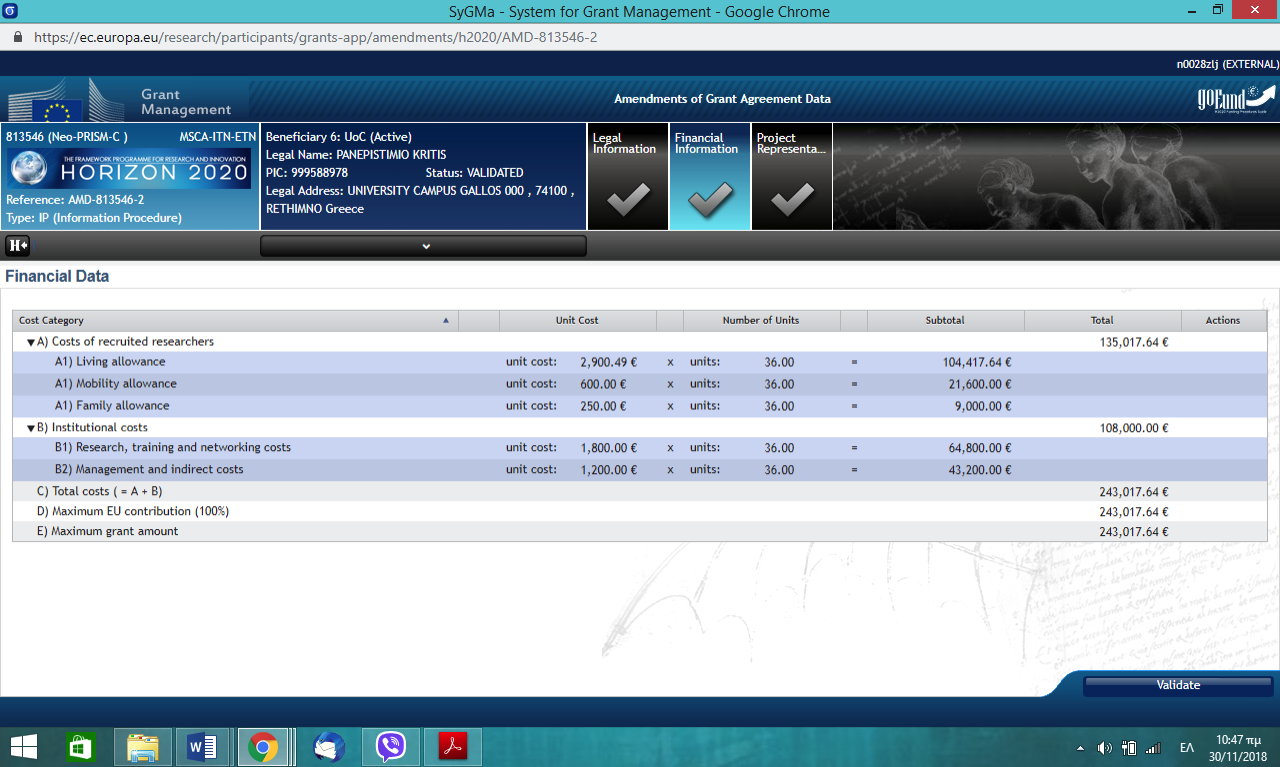

Supplement: S2 File — (DOCX) [file pone.0343364.s006.docx]
