# Supplementary material for: Child training in the Child ViReal Support Program: Combining iVR-based cognitive training and CBT techniques in a pilot study
Source: PLoS One. 2026 Feb 27;21(2):e0343364. doi: 10.1371/journal.pone.0343364 (PMC12948055; doi:10.1371/journal.pone.0343364)
Supplement: S3 File — (DOCX) [file pone.0343364.s007.docx]

**Determinants of Treatment Response to State-of-the-art Interventions for Attention Deficits: Child Temperament, Cognitive Profiles and Family Dynamics**

Iouliani Pachiti

Department of Psychology, University of Crete

Research Supervisor: Dr. Panayiota Dimitropoulou

Assistant Professor in Educational Psychology

**Research Protocol**

Attention deficits, which are often accompanied by symptoms of hyperactivity and impulsivity, are defined by the Diagnostic and Statistical Manual of Mental Disorders-5 (DSM-V; American Psychiatric Association, 2013) as Attention Deficit Hyperactivity Disorder (ADHD) provided that children meet specific diagnostic criteria. ADD/ADHD is a chronic neurodevelopmental disorder and is one of the most commonly diagnosed childhood developmental disorders (Maniadaki, 2019; Sekaninova et al., 2019). It is characterised by a persistent and developmentally inappropriate pattern of inattention, hyperactivity and impulsivity, which results to impairments in concentration, sustained attention, working memory as well as difficulties with impulse control (American Psychiatric Association, 2013; Maniadaki, 2019; Sekaninova et al., 2019).

Epidemiological data from various studies report that the incidence rate of this disorder ranges from 3% to 7% among the child population, while a meta-analysis of 102 studies conducted by Polanczyk and his colleagues (2007) reported that the global percentage of ADD/ADHD in the child population is 5.29% (Maniadaki & Kakouros, 2016; Polanczyk et al., 2007). As for the Greek population, research data report prevalence rates between 5-11% (Maniadaki & Kakouros, 2016). Palili and colleagues (2011) conducted one of the most important epidemiological studies to date concerning the Greek population using a longitudinal design study, where they systematically followed 2695 children from birth to adulthood in order to study the incidence of ADD/ADHD symptoms. The results of this study showed that at the age of 7 years old the incidence of hyperactivity symptoms was 7%, of inattention symptoms was 9.5% and of impulsivity symptoms was 7%. At the age of 18 years old, the same group of children was evaluated again and the evaluation showed that hyperactivity and impulsivity symptoms decreased to 3% and 0.3% respectively, but attention difficulties were still present in 7.3% of the sample (Palili et al., 2011).

Many of the children diagnosed with ADD/ADHD present comorbidity with other conditions and difficulties, such as disruptive behaviors, learning difficulties, internalizing problems (anxiety, depression), as well as difficulties in creating and maintaining social relationships, rejection by peer groups, reduced school performance and sleep impairments (Brassett-Harknett & Butler, 2005; Tarver et al., 2014). Children with ADD/ADHD also present deficits in executive functions that are in line with the structural differences observed in imaging studies on the frontal areas of their cerebral cortex. These deficits also become apparent from their lower performance scores when compared to typically developing children in tasks that measure dimensions of executive functions (eg. sustained attention, working memory etc.) (Sjöwall et al., 2013; Tarver et al., 2014).

Moreover, children with ADD/ADHD show impairments in recognizing, controlling and managing their emotions. These difficulties are a common feature observed in studies using parental reports, where parents mention that their children show low levels of emotional control and high levels of negative emotions (anger, distress), as well as in studies using emotion recognition tasks (Franke et al., 2018; Sjöwall et al., 2013).

In general, there is a considerable heterogeneity among people with attention deficits and hyperactivity/impulsivity presenting a wide spectrum of severity and symptoms both in neuropsychological and emotional deficits. This heterogeneity makes it necessary for individualized intervention regarding the deficits and needs presented by each child. Additionally, it is very important for interventions to consider and include child’s emotional functioning as it is a significant predictor for negative events in children’s lives including school drop-out and difficulties in family and social relationships (Franke et al., 2018; Sjöwall et al., 2013; Tarver et al., 2014).

As for the long-term course of ADD/ADHD, it has been found through longitudinal research studies that in a substantial number of cases the attention deficits do not remit in puberty, but persist into adulthood despite the decreasing levels of hyperactivity/impulsivity (Brassett-Harknett & Butler, 2007; Franke et al., 2018; Palili et al., 2011). Longitudinal studies also show that there are several concomitant problems through adolescence and adulthood for people with ADD/ADHD, such as a higher risk of developing drug addictions, especially if there is a comorbidity with disruptive behaviors (eg. conduct disorder), an increased risk for violent behavior and incarceration, as well as more car accidents (Brassett-Harknett & Butler, 2007; Franke et al., 2018).

Alongside the intra-individual difficulties presented by children coping with ADD/ADHD, there are substantial changes in family contexts and dynamics, where relationships are often disturbed by the hearing of the diagnosis for the child. Meta-syntheses and meta-analyses of qualitative and quantitative research studies respectively showed that parents of children diagnosed with ADD/ADHD experience high levels of parental stress and negative emotions while trying to manage family routines and their children’s behaviors (Corcoran et al., 2017; Miller & Brooker, 2017; Theule et al., 2013). Even typical family routines (eg. morning routines, school preparation, bedtime preparation) are a daily challenge for parents, while their parental stress seems to be channeled into several domains of their lives, such as their physical health, as well as their psychological, marital and professional functionality. In several studies that are referred into the meta-synthesis of studies conducted by Corcoran and colleagues (2017), several parents reported that they had to change or resign from their jobs in order to be able to better manage their children’s behaviors (Hallberg et al., 2008; Ηο et al., 2011; Moen et al., 2011, as cited in Corcoran et al., 2017), while their marital relationships are disrupted for a variety of reasons (eg. the child constantly seeks attention reducing the time between parents, mothers play a greater role in managing children’s behaviors than fathers etc.) (Corcoran et al., 2017; Theule et al., 2013). Furthermore, parental relationships with the child are also disrupted and are characterized by more conflicts and more authoritarian and controlling parenting techniques (Lifford et al., 2008). Therefore, it seems important to provide support and assistance to parents of children diagnosed with ADD/ADHD regarding their emotional self-management skills, since they experience considerable stress and negative emotions, as well as parent training on optimal parental practices for managing their children’s behaviors.

**Research Domain of Criteria (RDoC)**

The National Institute of Mental Health (NIMH) has developed, the last few years, a new research approach in an effort to study the difficulties and deficits that a lot of individuals present through a dimensional approach opposing to the categorical approach followed by DSM and ICD, which is referred to as Research Domain of Criteria (RDoC) (Cuthbert, 2015). This research approach does not intend to replace the two existing diagnostic manuals and form a new diagnostic categorical system. Its goal and rationale is for researchers to study and integrate information from different units of analysis (from genomics to self-reports) in order to give a better understanding of the deficits that some individuals present. It encourages researchers to investigate these deficits assessing them on a range of functioning of neurobiological, cognitive and behavioral capacities representing them along a continuum of greater or lesser degrees of health or adaptation (Musser & Raiker, 2019; Garvey et al., 2016).

Moreover, the aim of research studies adopting the RDoC approach is to develop appropriate conceptual models that will provide the necessary knowledge between and within these levels of information (genes, molecules, cells, circuits, physiology, behavior and self-reports) in order to give a better understanding of the complexity of the various symptoms, not merely on a behavioral level but on a neural circuitry, which underlies typical and atypical behaviors and symptoms. The goal is to direct and develop appropriate and more effective drug and behavioral interventions targeting in multiple domains and based on the biological basis of the difficulties (Garvey et al., 2016).

Therefore, new research efforts, which adopt the RDoC approach and study the difficulties and deficits of children with ADD/ADHD have to be enriched by measurements that are not only limited to parents’ and teachers’ reports or adolescents’ self-reports on behavioral scales, but that provide data regarding the neurobiological basis of behaviors and symptoms presented (eg. imaging methods, fMRI, eye movements etc.) in order to link and integrate information between levels of analysis.

The oculomotor system can provide this kind of data, since measurements with an eye-tracker gives information for the neural basis of both reflexive and voluntary behavior (Luna et al., 2008. Rommelse et al., 2008). The execution of an eye movement is the result of a complex interaction of various cognitive processes and thus, the oculomotor behavior can provide information about these processes as well as information for observed deficits (Rommelse et al., 2008). Furthermore, the addition of cognitive demands to oculomotor tasks (eg. tasks with antisaccadic movements) results in voluntary eye movements to require the use of higher-level cognitive processes as they produce neuronal activity throughout the cerebral cortex in anticipation of a planned response, thus allowing for the identification of brain regions involved in these cognitive processes (Basso, 1998, as cited in Luna et al., 2008).

**Intervention and ADHD**

In view of the above considerations, it can be noted that attention deficits and hyperactivity/impulsivity, as well as their comorbidities, occur in a significant proportion of the child population and affect children in important areas of their lives (school performance, friendships etc.) and their intra-family relationships. Therefore, it is evident that both children with ADD/ADHD and their parents need appropriate support and intervention in order to manage their behavioral and emotional disturbances, while parents need to practice their emotional self-management skills since they have to cope with these difficult behaviors.

Even though there is currently no cure for ADD/ADHD, there are interventions that have been designed and implemented aiming at the management of observed symptoms and improvement of functionality (Schellack et al., 2019; Tarver et al., 2014). Evidence-based interventions that are widely accepted for the management of ADD/ADHD symptoms include pharmacotherapy, psychoeducational interventions or a combination of the two (Anton et al., 2009. Maniadaki & Kakouros, 2016). Pharmacotherapy includes stimulants (eg. methylphenidate and amphetamine) and non-stimulants (eg. atomoxetine). Research data demonstrate the efficacy of medication in reducing core ADD/ADHD symptoms since stimulants appear to improve inhibition and self-regulation of behavior, while non-stimulants help on the improvement of attention and concentration (Maniadaki & Kakouros, 2016; Schellack et al., 2019; Tarver et al., 2014).

Although pharmacotherapy has proven its efficacy, many experts and parents are concerned about the potential side effects of medication in children. Some of these side effects include decreased appetite, sleep problems, headaches, nausea, fatigue and mood swings (Franke et al., 2018; Maniadaki & Kakouros, 2016; Zachor et al., 2009). In addition, medication may help with the improvement of primary ADD/ADHD symptoms while offering short-term improvement in children’s social interactions and school performance, however, this improvement is not sustained in the long run and does not help their general functioning in social relationships and school life (Maniadaki & Kakouros, 2016; Tarver et al., 2014).

Psychoeducational interventions include: (a) behavior modification programs with key components of these programs being parent training and school interventions, (b) cognitive-behavioral interventions and (c) social skills training (Anton et al., 2009; Maniadaki & Kakouros, 2016; Tarver et al., 2014).

Behavior modification interventions are based on operant conditioning’s and social learning’s principles and aim at increasing the frequency of adaptive child behaviors, at reducing non-compliant or disruptive behaviors and at developing new functional behaviors that did not previously exist in child’s repertoire. Their application is mainly based on parents’ and teachers’ training on differential reinforcement, as well as on reward systems (eg. token or point system, social reinforcement, time-out etc.) (Anton et al., 2009; Maniadaki & Kakouros, 2016; Zachor et al., 2009).

The main goal of parent training is for parents to gain further knowledge, understanding and clarification of the difficulties and deficits that their children have to cope with, to address any misconceptions they may have about themselves and their children, as well as to replace the dysfunctional interaction patterns they have with their children with more functional and positive patterns (Johnston & Park, 2015; Lee et al., 2012; Pfiffner & Haack, 2014). In essence, they are trained to focus on the children’s positive behaviors rather than on the negative ones they usually focus on, on learning how to set rules and give efficient requests, as well as on how to use differential reinforcement techniques in order to effectively handle their children’s reactions. They are also trained in the application of home-reward systems (eg. list with rewards/privileges that the child may earn when they present adaptive behaviors, exchange of points/tokens earned for presenting these behaviors) and on the generalization of these techniques on behaviors that the child present in public places (Anton et al., 2009; Johnston & Park, 2015; Maniadaki & Kakouros, 2016; Lee et al., 2012; Zachor et al., 2009). Parent training on behavior modification programs seems to improve child’s, parents’ and the whole family’s functionality as well as parents’ sense of self-efficacy regarding their management skills for the behaviors their child exhibit. Moreover, there seems to be an increase in parental self-esteem, improved parental practices and a decrease in parental stress and negative emotions (Pfiffner & Haack, 2014; Zachor et al., 2009).

Alongside the behavior modification programs, more emphasis has been placed, in recent years, on the concept of mindful parenting and parent-focused mindfulness-based interventions (Miller & Brooker, 2017). Mindfulness refers to the awareness arising through paying deliberate attention on the present moment and the observation of a current experience as it arises without any judgment, analysis or evaluation (Han et al., 2019). Mindful parenting consists of nonjudgmental and present-centered awareness during parent-child interactions. Research studies that examined the pathway of mindful parenting’s effect on children’s internalizing and externalizing problems showed that mindful parents use more positive parenting practices. They are also better at distinguishing between cognitive, emotional and behavioral experiences in contrast to parents who have lower levels of mindful parenting, thus reducing the likelihood of engaging in inappropriate interactions with their children (Han et al., 2019).

Moreover, parents who mindfully interact with their children have higher quality relationships with their children than those who have less mindful interactions (Duncan et al., 2009, as cited in Han et al., 2019; Parent et al., 2016) and this is related to better psychosocial adjustment and fewer problem behaviors in children (Han et al., 2019; Parent et al., 2016). Therefore, mindfulness-based interventions have been, in recent years, integrated into intervention programs for parents of children with ADD/ADHD, as they seem to provide self-care opportunities to parents. Participants in these interventions report improved coping with stress, a reduction in distress, and reduced symptoms of psychopathology (Miller & Brooker, 2017).

As for cognitive-behavioral interventions (CBT), they consist an extension of behavioral modification interventions that focus on the interaction between an individual’s cognition, emotions and behavior (Flores & Parra, 2014. Tarver et al., 2014). Their main goal is to teach children to exercise more effective control over their thoughts, feelings and cognitive functions as a mean of self-management and improvement of their self-control over their concentration, attention and behavior. Thus, in CBT, children with ADD/ADHD are taught to use self-instruction, self-evaluation, self-reinforcement as well as problem solving and motivational strategies using role-playing in order to self-assess and self-correct their behavior and improve their cognitive skills such as attention and working memory. These strategies have positive impact on different areas of their lives such as adapting to rules and routines of daily living, developing and maintaining social relationships and healthy self-esteem and better problem solving (Flores & Parra, 2014; Maniadaki & Kakouros, 2016; Schellack et al., 2019).

In addition to the above forms of intervention, it is particularly important to educate children with ADD/ADHD in acquiring social skills (social skills training), because, as mentioned above, they usually have difficulties in establishing and maintaining friendly relationships, while often experiencing rejection by peer groups. Social skills training involves role-playing on a wide range of skills, such as asking questions, collaborating, maintaining a discussion, following rules and joining a peer group (Maniadaki & Kakouros, 2016; Pfiffner & Haack, 2014; Zachor et al., 2009). Research studies on the effectiveness of these interventions have not yielded any significant results. A possible reason for this is that the skills learned during the training are not transferred and generalized into children’s daily life (Evans et al., 2014).

To sum up on existing evidence-based interventions for children with ADD/ADHD, medication has proven to reduce primary ADD/ADHD symptoms, but without long-term improvement on children’s functionality. On the other hand, psychosocial interventions help parents and children to acquire those essential skills on managing the difficulties presented and their effectiveness and efficacy has been proven by research studies as well as by meta-analyses of research studies (Johnston & Park, 2015; Fabiano et al., 2009, as cited in Tarver et al., 2014). Even though a recent meta-analysis of studies conducted by Sonuga and his colleagues (2013, as cited in Franke et al., 2018 and Tarver et al., 2014) showed that the effect sizes of psychosocial interventions’ impact on the reduction of ADD/ADHD core symptoms were low, it is important to bear in mind that these interventions have positive effects both on the management of children’s behaviors and on secondary symptoms and difficulties such as the improvement of their social interactions, the reduction of parental stress and enhancement of parental sense of self-efficacy, as well as the improvement of children’s self-control over their behavior resulting in long-term adaptation to their environment (Anton et al., 2009; Maniadaki & Kakouros, 2016; Pfiffner & Haack, 2014).

The most effective improvement in the difficulties and deficits that children with ADD/ADHD present is related to multilevel intervention programs, which are tailored to each child’s cognitive and behavioral levels. These programs take advantage of the combination of parent-focused psycho-education and child-focused cognitive-behavioral intervention (Anton et al., 2009; Maniadaki & Kakouros, 2016; Rajeh et al., 2017) and, in many cases, pharmacotherapy.

Overall, both medication and psychosocial interventions have been shown to be effective, but they seem to lack, according to latest research data, an important dimension. This dimension relates to the fact that children do not learn skills in an environment similar to the one in which they are expected to transfer and generalize those skills. This limitation is addressed by the flexibility offered by Virtual Reality (VR) technology (Bashiri et al., 2017), which is discussed in the next section.

**Virtual Reality**

VR technology enables a computer-based simulation of a three-dimensional image or environment that one can interact with through multiple sensory channels allowing them to behave as they would in real-world situations and environments (Bashiri et al., 2017; Shema-Shiratzky et al., 2019; Wang & Reid, 2011). This technology has been used for several years in various fields such as education, training, entertainment, military training, medical and surgical training and therapeutic intervention (Anton et al., 2009; Bashiri et al., 2017).

Several studies have shown that the possibilities of VR technology in intervention for children with difficulties (eg. ADHD, autism, cerebral palsy etc.) include the flexibility regarding each child’s requirements, the therapist’s ability to control and adapt intervention’s elements based on each child’s individual level, the sense of immersion on the part of the child in an environment similar to the one in which new acquired skills are expected to be transferred and the possibility to learn and acquire new skills in a safe environment (Jeffs, 2010; Parsons et al., 2017; Wang & Reid, 2011).

In addition to the above possibilities, using VR technology in the intervention for children with diagnosed ADD/ADHD also offers the ability for individualized activities for each child or subgroup of children depending on the difficulties they present and on each child’s interests and preferences (Bashiri et al., 2017; Wang & Reid, 2011). Therefore, a therapist can modify a complex virtual environment by removing several distractors in order to increase children’s attention span, while at the same time they can maximize feedback’s impact, which can be immediate and it is significant for children experiencing attention deficits.

Furthermore, the use of VR in intervention provides control over the design of the intervention plan with the integration of structured and systematic educational strategies by the therapist, while it becomes somewhat more interactive and fun enhancing thus children’s motivation and engagement (Anton et al., 2009; Bashiri et al., 2017; Wang & Reid, 2011). A literature review of research studies conducted by Bashiri and her colleagues (2017) showed that the use of VR reduces the costs and time of intervention plans and improves working memory, executive functions and cognitive processes (eg. attention).

In addition, Shema-Shiratzky and her colleagues (2019) conducted a pilot study in which 14 school-aged children with diagnosed ADHD took part, in order to examine the efficacy of a combined motor-cognitive training using VR to enhance children’s behavior, cognitive function and dual tasking. After the training, parents reported significant improvements in children’s social problems and psychosomatic behavior. There was also an improvement in directing and selective attention, which were reflected in better executive function scores and an improvement in working memory, although the attention index score (focused attention and vigilance) remained unchanged (Shema-Shiratzky et al., 2019).

VR technology, in general, could be integrated into a multimodal intervention plan for children with ADD/ADHD, as it offers opportunities and possibilities that traditional intervention programs cannot offer, such as high ecological validity, which can increase the chances of transfer and generalization of new acquired skills in the real world (Anton et al., 2009; Bashiri et al., 2017).

On the basis of the above information, it can be said that attention deficits and hyperactivity/impulsivity faced by a significant proportion of child population significantly affect various areas of their school, social and family life, making it necessary for both children themselves and their parents to receive intervention and support (Corcoran et al., 2017; Sjöwall et al., 2013; Tarver et al., 2014). In addition, it seems important to provide a combination of interventions that can be individualized and tailored to each child’s difficulties. Alongside that, it is considered particularly important to determine and identify the elements that differentiate each child (eg. cognitive profile) and their family environment (eg. family relationships) since these elements mediate the outcome of the intervention and play a significant role in the individualization of the interventions proposed (Haack et al., 2017; Hinshaw, 2007).

Therefore, the aim of the proposed research study is to examine the regulatory factors that influence the outcome of interventions in children with attention deficits such as children’s cognitive profiles and their family environment. Thus, researchers proposing the current study are expected to develop a *longitudinal multimodal intervention program* *for children with attention deficits and/or hyperactivity/impulsivity*. This intervention program will include evidence-based components that are usually used for children with attention deficits and hyperactivity/impulsivity combined with the use of VR technology. In particular, it is expected to include: (a) child training on cognitive and emotional self-regulation skills based on CBT principles, supported by VR simulation games, (b) parent training on optimal parental practices and (c) parent counseling/support aiming to reduce the psychological burden of ADHD and enhance parental emotional self-regulation skills.

The hypotheses of the proposed research study are as follow:

1. Children of parents presenting a supportive parenting style will have higher levels of psychosocial adjustment in contrast to children of parents using authoritarian or strict parenting practices
2. Intervention group’s children will show improvement on the behavioral problems they present, better scores on attention tasks and enhancement of their psychosocial adjustment compared to the control group children after the implementation of the intervention
3. Intervention group’s parents will show decrease on parental stress and increase on their sense of self-efficacy compared to the parents of the control group after the intervention
4. We will examine the parenting styles in relation to the intervention results in order to identify how each parenting style mediate the outcome of the intervention
5. We will examine children’s characteristics and cognitive profiles in relation to the intervention results in order to identify how these elements mediate the outcome of an intervention program.

**Method**

**Participants**

It is expected that, in the proposed research study, data will be obtained from approximately 80 children aged 9-12 years old (fourth to sixth elementary school class) who have already been diagnosed with Attention Deficit Hyperactivity Disorder (40 children for the experimental group and 40 children for the control group). Children and their parents will be located at the Community Children and Adolescents’ Mental Health Centers (Koi.Ke.PS.Y.P.E) in Heraklion, Rethymnon and Attica, as well as at the Educational and Counseling Support Centers (K.E.S.Y.) in Rethymnon, Heraklion, Chania and Attica. This sample size is considered appropriate since, in a significance level of 5%, it is estimated that 36 participants in each group will be needed to obtain a statistical power of 90% to reject the null hypothesis of no difference between these conditions. This calculation is based on analyses of between-subjects and within-subjects’ repeated measures.

The research team will reach out to the experts and staff of Koi.Ke.PS.Y.P.E. and K.E.S.Y. centers in order to inform and explain them the aim of the study so that, on their part, they will inform the parents of children diagnosed with ADD/ADHD. Participants’ information and consent forms for the study will be provided to the parents and those who are willing to participate in the research study will complete and return them to the research team. Children’s inclusion criteria will be age, gender (in an attempt to have a boy-girl ratio), non-medication, non-participation in other intervention programs based on cognitive-behavioral approach and no comorbidities. Exclusion criteria will be the non-fulfillment of the inclusion criteria.

After the selection, children will be randomly assigned to two groups (experimental group and control group), where the experimental group will take part in the intervention program (parent training and child training). For ethical reasons, the parents of the control group will take part in a psycho-educational seminar about the difficulties that their children experience after the post-intervention assessment at the end of the intervention program for the experimental group.

**Materials**

The tools that will be used to collect data for the proposed study have been widely used in similar studies with children with attention deficits and hyperactivity/impulsivity. We will use questionnaires to obtain demographic data and child history from parents, as well as data from the assessment that children received at mental health’s centers following informed consent from children’s parents/guardians.

In addition, we will use questionnaires for the parents since it is important to collect data regarding parental style, sense of parental self-efficacy and parenting stress, as these are factors that may influence the outcome of parent training. We plan to use the following questionnaires:

1. Questionnaire for Demographic Data (see Appendix A),
2. Parenting Styles and Dimensions Questionnaire (for Greek fathers and Greek mothers) (adaptation and standardization in Greek population by Maridaki-Kassotaki, 2009 and Antonopoulou & Tsitsas, 2011, see Appendix A), which is a parental self-report questionnaire that assesses parental typology regarding parent-child relationship, communication and rearing methods and reveals four parental types, the authoritative, the authoritarian, the supportive and the strict parent,
3. Parenting Stress Index-Short Form (Abidin, 1995, translation in Greek by Leze, 2013, see Appendix A), which measures parental stress as a variable influenced by child’s characteristics, parental characteristics and the situations surrounding their relationship and gives an observation of parental stress levels, as well as inadequate childrearing methods and child’s adaptability in the family environment,
4. Tool to Measure Parenting Self-Efficacy (TOPSE, Kendall & Bloomfield, 2005, translation and adaptation in Greek population by Karakosta-Stefanopoulou & Malikiosi-Loizou, 2013, see Appendix A), which measures parents’ self-efficacy perceptions on specific domains of their parental role,
5. Greek ADHD-IV Rating Scale (DuPaul, Power, Anastopoulos, & Reid, 1998, adaptation and standardization in Greek population by Kalantzi-Azizi, Aggeli, & Eustathiou, 2006, see Appendix A), which measures the frequency of ADHD symptoms.

Along with the data that will be obtained by parents, it is expected that the research team will collect data from the children who will participate in the proposed research study. Initially, separate participants’ information and consent forms will be given to children (see Appendix B), in order for them to decide whether they would like or not to take part in this study. Also, children will be reassured that any information received from them during the study will be confidential and that they may withdraw their participation at any time they wish. We plan to use the following measurements for children’s evaluation:

1. Questionnaire for Demographic Data (see Appendix A)
2. Wechsler Intelligence Scale for Children-V (WISC-V) in order to obtain data regarding their cognitive profile (or specific subscales from WISC-V),
3. Psychosocial Adjustment Test (Greek test, Hatzichristou et al., 2008, see Appendix A), which is a self-report scale for children aged 10-12 years old that provides information for certain characteristics of psychosocial adjustment as assessed by children themselves aiming to the identification of both skills and deficits on social and emotional domains, on social adjustment as well as interpersonal adjustment,
4. Children’s Perceptions of Parenting Styles and Dimensions Questionnaire (for fathers and mothers) (see Appendix A), in order to compare the results with the answers from the PSDQ completed by parents and find out how children perceive the parenting style of their parents and whether the results of children’s perceptions are in line with the results of parents’ perceptions, and
5. Parental Acceptance/Rejection Questionnaire-Short Form (PARQ-Child, Rohner, 2002, translation and adaptation in Greek population by Tsaousis, Giovazolias, & Mascha, 2012, see Appendix A), which measures children’s perceptions on parental acceptance and rejection.

In addition to the above measurements, it is expected that data will also be collected from computerized cognitive tests that have scientifically proven their relationship with the difficulties that children with ADD/ADHD present. The research team will use a Posner-type task adapted for children as it assesses attention and the ability to shift attention and a Continuous Performance Test (CPT) adapted for children, which measures sustained and selective attention since individuals are expected to respond to a stimulus target and ignore disruptive and irrelevant stimuli. Moreover, during the execution of these computerized tasks, eye-tracking measurements are expected to be obtained using an eye-tracker that will be embedded on the computer screen. The aim is to collect data of children’s fixations and data on whether they can control reflexive/autonomic eye movements towards disruptive stimuli (eg. during CPT). Cognitive tests’ and eye-tracking’s measurements have to be taken in order to be compared with those at the end of the intervention program to examine and determine whether the intervention was effective on sustained and selective attention in which children with ADD/ADHD present deficits.

All of the above measurements (parents’ questionnaires, children’s questionnaires and computerized cognitive tests) are considered necessary as they will provide information on potential determinants of the intervention’s outcome, while they will give data from different levels of analysis as determined by the RDoC research approach.

**Research’s and Intervention’s Implementation Procedure**

Initially, the research team will contact and inform the experts and staff at Koi.Ke.PS.Y.P.E. and K.E.S.Y. centers for the research study in order for them to inform parents of children diagnosed with ADD/ADHD for the study. Participants’ information and consent forms (see Appendix B) will then be provided to interested parents in order for the research team to gather a number of interested participants.

Prior to the start of the intervention program, a pilot study with a small number of participants will be conducted to investigate the feasibility and usability of VR technology, as it is a new method introduced into the intervention plan. After this pilot study, the necessary changes will be made based on children’s statements on the use of equipment and VR as part of the intervention.

Subsequently, children will be selected based on inclusion criteria (age, gender, non-treatment, no-comorbidities). The participants’ sample will then be randomly divided into two groups (intervention group and control group).

After the selection and distribution of participants in the two groups, the research team will contact them in order to inform them that they have been selected to take part in the research study. Two assessment sessions will follow, where parents will be given to complete the questionnaires described above (parents’ assessment prior to intervention, T1). Prior to children’s assessment, they will be given a separate participant’s information and consent form in which they will get all the necessary information for the study in order for them to decide whether they would like to take part or not (see Appendix B). After they give their informed consent, children will be evaluated with the measurements described above (children’s assessment prior to intervention, T1).

Afterwards, the parents of children that will form the intervention group are expected to participate in an 8-10 week psycho-educational program on behavior modification techniques, training on optimal parental practices and on mindfulness techniques aiming at the reduction of stress they are expected to experience and the enhancement of emotional self-management.

The intervention program will include an initial session where it will be given an overview of ADD/ADHD and information in order to address any misconceptions that parents might have, while parents will have the opportunity to express their feelings regarding the difficulties their children experience. Also, in this initial session, confidentiality will be ensured from parents towards the group and the sessions. Then, in the next sessions, parents will get trained in various behavioral techniques (eg. positive attention on child’s positive behaviors, set of family rules, formation of parents’ requests, positive reinforcement for child’s adaptive behaviors, home-reward system, generalization of techniques etc.) and on mindfulness techniques. In addition, it is expected that, during the training sessions, parents will express their concerns about the training and their children’s behavior and guidance will be given based on their needs.

As far as children’s intervention program is concerned, this is expected to consist of 18-20 weekly sessions (1 hour/session) and will include training on cognitive and emotional self-regulation skills based on cognitive-behavioral principles. VR technology will also be used in the intervention program since it offers the opportunity to train children in real-world simulated environments. In essence, it is expected that children will be trained in techniques of self-evaluation, self-reinforcement and self-regulation of their behavior and emotions. It is also expected that through VR simulation games children will perform various tasks whose difficulty and content will change according their level and skills, while they will be trained in sustaining and shifting attention skills. Children’s training will be combined with parents’ training (eg. they will be able to change game points with privileges in home-reward system).

After children’s sessions will be completed, a boost session with the parents will be held to discuss any difficulties that might arise since the end of their psycho-education sessions and a review of the behavioral techniques and strategies will take place. Parents will also have to complete the same questionnaires, which have been described above, in order to examine whether any changes will occur after the intervention on any of these measures (parents’ assessment post-intervention, T2). Furthermore, children will be re-evaluated with the tests that will be used in their initial assessment in order to examine whether any changes will occur as a result of the intervention program on their psychosocial adjustment, perceptions of parental acceptance/rejection, perceptions of their parents’ rearing methods and parental style, as well as on their attention measures during the computerized cognitive tests (children’s assessment post-intervention, T2). During the execution of the computerized cognitive tests eye movement measurements will also be obtained.

The research team will then contact parents and children again after about 3 months to conduct a final evaluation (follow-up assessment, T3), where parents and children will complete the same questionnaires and children will be evaluated with the computerized cognitive tests in order to examine whether any changes resulting from the intervention program will be sustained in the post-intervention period.

Parents and children in the control group will complete the same questionnaires and computerized cognitive tests using eye-tracking measures as well, at the same times as participants in the intervention group. For ethical reasons, parents of control group will take part in a psycho-educational seminar about the difficulties that their children experience and some form of training on parental practices after the post-intervention assessment (T2).

**Expected Results – Research Study’s Usefulness**

The proposed research study involves the design, development and implementation of a longitudinal multimodal intervention program for children with attention deficits and hyperactivity/impulsivity, which will include parent training and child training. Researchers expect to investigate the factors that must be taken into account when designing and implementing an intervention program. The data that will be collected from the questionnaires and computerized cognitive tasks will provide information on children’s cognitive profile and characteristics as well as information for the family dynamic (family relationships, parental style, parenting stress). These measurements will be studied in relation to the results of the intervention to determine whether they play a regulatory role on the outcome of an intervention. Mixed model analyses using repeated measures covariance, group comparisons as well as analyses used to examine variables that may have a regulatory effect on the outcome of an intervention would be conducted.

An important differentiating and innovating element of this proposed research study is the integration of VR technology in the intervention program. VR technology, as mentioned above, offers opportunities that traditional types of intervention cannot offer, enhancing the chances and ability to transfer and generalize new acquired skills to environments other than the intervention environment. Also, VR technology provides a more interactive and entertaining environment where the intervention can take place thus enhancing children’s motivation and engagement.

In essence, finding out about the factors which contribute or not to the effectiveness of an intervention will offer a better understanding of the need for evidence-based, individualized treatments for children so as to prevent negative developmental trajectories (eg. development of other difficulties and disorders, school failure, long-term adjustment difficulties etc.).

The benefits of the implementation of the proposed research study are particularly important for students with neurodevelopmental disorders such as ADD/ADHD and their families since the results will contribute to a better understanding of their difficulties’ impact on the psycho-emotional and learning areas of their lives and their families’ system. Also, the development of an intervention program for children’s better adaptation and development could consist an empirically based effective practice that could be widely applied for populations with similar characteristics.

**Research’s Ethics**

**Informed Consent**

Parents and children who are expected to participate in the proposed research study will receive all relevant information for the purpose of the research and the procedure to be followed through the participants’ information and consent forms that will be provided to them. Also, if they wish to receive further information they could personally contact the research team. Participants will also be informed that they may withdraw their participation at any time they wish and data collected by the time of their withdrawal will be destroyed.

**Personal Data Protection**

All information concerning the participants during the proposed research study and intervention program will remain confidential and any publication resulting from this study will present the findings anonymously. The data obtained from the participants (parents and children) will be used exclusively for research purposes in the context of this doctoral dissertation. The collection and processing of participants’ personal data will fully comply with the requirements of the European Union’s General Data Protection Regulation 679/2016.

Both research data collected during the study and the material resulting from the implementation of the intervention program will be coded with numbers. Therefore, the participants’ personal data and the data collected from measurements will not appear anywhere so participants will not be able to get identified. Additionally, any personal information that will be collected for research study’s needs (eg. contact details) will be kept confidential and under research team’s and especially Research Supervisor’s supervision and responsibility and they will be locked in a safe place (at *the Applied Psychology Laboratory of the University of Crete’s Research Center for the Humanities, the Social and Education Sciences*). Only the research team of the study will have access to any data collected and they are committed to the confidentiality of participants’ data.

Researchers are also committed to strictly adhere to confidentiality and data protection regulations following the relevant guidelines as set out in both Greek and European Union’s legislations as well as from Hellenic Data Protection Authority (HDPA).

Upon completion of the research study and in collaboration and consultation of both Neo-PRISM-C program’s coordinator (Professor T. Papadopoulos, University of Cyprus, Department of Psychology) and University of Crete’s Data Protection Officer (DPO) will be set out the final management and destruction of participants’ data based on European Union’s guidelines.

**Complaints**

Any complaints that may arise from participants could be submitted orally or written to the doctoral student as well as via phone call or email to one of the research supervisors (Dr. Dimitropoulou and Dr. Tsaousis). Contact details of both doctoral student and research supervisors will be submitted on the participants’ information and consent forms that will be given to participants in two copies (one for research’s purpose and one for their own use).

Also, for any complaints regarding the conduct of the research, participants could contact University of Crete’s Research Ethics Committee and for any complaints regarding the management of their personal data they could contact University of Crete’s DPO and in any case the HDPA.

References

American Psychiatric Association. (2013). *Diagnostic and statistical manual of mental disorders* (5th ed.). <https://doi.org/10.1176/appi.books.9780890425596>

Anton, R., Orpis, D., Dobrean, A., & David, D. (2009). Virtual reality in the rehabilitation of attention deficit/ hyperactivity disorder. Instrument construction principles. *Journal of Cognitive and Behavioral Psychotherapies*, *9*(2), 235-246. <http://search.ebscohost.com/login.aspx?direct=true&db=asn&AN=44483007&site=ehost-live>

Bashiri, A., Ghazisaeedi, M., & Shahmoradi, L. (2017). The opportunities of virtual reality in the rehabilitation of children with attention deficit hyperactivity disorder: a literature review. *Korean Journal of Pediatrics, 60*(11), 337-343. <https://doi.org/10.3345/kjp.2017.60.11.337>

Brassett-Harknett, A., & Butler, N. (2007). Attention-deficit/hyperactivity disorder: an overview of the etiology and a review of the literature relating to the correlates and lifecourse outcomes for men and women. *Clinical Psychology Review*, *27*(2), 188-210. <https://doi.org/10.1016/j.cpr.2005.06.001>

Corcoran, J., Schildt, B., Hochbrueckner, R., & Abell, J. (2017). Parents of children with attention deficit/hyperactivity disorder: A meta-synthesis, part I. *Child and Adolescent Social Work Journal*, *34*(4), 281-335. <https://doi.org/10.1007/s10560-016-0465-1>

Cuthbert, B. N. (2015). Research Domain Criteria: toward future psychiatric nosologies. *Dialogues in Clinical Neuroscience, 17*(1), 89-97.

Evans, S. W., Owens, J. S., & Bunford, N. (2014). Evidence-based psychosocial treatments for children and adolescents with attention-deficit/hyperactivity disorder. *Journal of Clinical Child & Adolescent Psychology*, *43*(4), 527-551. <https://doi.org/10.1080/15374416.2013.850700>

Flores, G. W. R., & Parra, V. A. B. (2014). Cognitive behavioral treatment in children with attention deficit hyperactivity disorder. *Revista de Psicología:(Universidad de Antioquía)*, *6*(2), 79-94.

Franke, B., Michelini, G., Asherson, P., Banaschewski, T., Bilbow, A., Buitelaar, J. K., Cormand, B., Faraone, S. V., Ginsberg, Y., Haavik, J., Kuntsi, J., Larsson, H., Lesch, K-P., Ramos-Quiroga, J. A., Rethelyi, J. M., Ribases, M., & Reif, A. (2018). Live fast, die young? A review on the developmental trajectories of ADHD across the lifespan. *European Neuropsychopharmacology*, *28*(10), 1059-1088. <https://doi.org/10.1016/j.euroneuro.2018.08.001>

Garvey, M., Avenevoli, S., & Anderson, K. (2016). The national institute of mental health research domain criteria and clinical research in child and adolescent psychiatry. *Journal of the American Academy of Child & Adolescent Psychiatry*, *55*(2), 93-98. <https://psycnet.apa.org/doi/10.1016/j.jaac.2015.11.002>

Haack, L. M., Villodas, M., McBurnett, K., Hinshaw, S., & Pfiffner, L. J. (2017). Parenting as a mechanism of change in psychosocial treatment for youth with ADHD, predominantly inattentive presentation. *Journal of Abnormal Child Psychology*, *45*(5), 841-855. <https://doi.org/10.1007/s10802-016-0199-8>

Han, Z. R., Ahemaitijiang, N., Yan, J., Hu, X., Parent, J., Dale, C., DiMarzio, K., & Singh, N. N. (2019). Parent mindfulness, parenting, and child psychopathology in China. *Mindfulness*, 1-10. <https://doi.org/10.1007/s12671-019-01111-z>

Hinshaw, S. P. (2007). Moderators and mediators of treatment outcome for youth with ADHD: Understanding for whom and how interventions work. *Journal of Pediatric Psychology*, *32*(6), 664-675. <https://doi.org/10.1093/jpepsy/jsl055>

Jeffs, T. L. (2010). Virtual reality and special needs. *Themes in Science and Technology Education*, *2*(1-2), 253-268.

Johnston, C., & Park, J. L. (2015). Interventions for attention-deficit hyperactivity disorder: a year in review. *Current Developmental Disorders Reports*, *2*(1), 38-45. <https://doi.org/10.1007/s40474-014-0034-2>

Lee, P. C., Niew, W. I., Yang, H. J., Chen, V. C. H., & Lin, K. C. (2012). A meta-analysis of behavioral parent training for children with attention deficit hyperactivity disorder. *Research in Developmental Disabilities*, *33*(6), 2040-2049. <https://doi.org/10.1016/j.ridd.2012.05.011>

Lifford, K. J., Harold, G. T., & Thapar, A. (2008). Parent–child relationships and ADHD symptoms: a longitudinal analysis. *Journal of Abnormal Child Psychology*, *36*(2), 285-296. <https://doi.org/10.1007/s10802-007-9177-5>

Luna, B., Velanova, K., & Geier, C. F. (2008). Development of eye-movement control. *Brain and Cognition, 68*(3), 293-308. <https://doi.org/10.1016/j.bandc.2008.08.019>

Maniadaki, K. (2019). Attention deficit/hyperactivity disorder: A real disorder throughout the lifespan. *JSM Pediatrics and Child Health, 4*(3), 1-3.

Μανιαδάκη, Κ., & Κάκουρος, Ε. (2016). *Η διαχείριση της ΔΕΠ-Υ. Από τη θεωρία στην πράξη*. Gutenberg.

Miller, C., & Brooker, B. (2017). Mindful programming for parents and teachers of children with ADHD. *Complimentary Therapies in Clinical Practice*, *28*, 108-115. <https://doi.org/10.1016/j.ctcp.2017.05.015>

Musser, E. D., & Raiker Jr, J. S. (2019). Attention-deficit/hyperactivity disorder: An integrated developmental psychopathology and Research Domain Criteria (RDoC) approach. *Comprehensive Psychiatry*, *90*, 65-72. <https://doi.org/10.1016/j.comppsych.2018.12.016>

Aήνα: )﷽δολογικο Ακα εμφάνισηςικ2000Palili, A., Kolaitis, G., Vassi, I., Veltsista, A., Bakoula, C., & Gika, A. (2011). Inattention, hyperactivity, impulsivity—epidemiology and correlations: A nationwide greek study from birth to 18 years. *Journal of Child Neurology*, *26*(2), 199-204. [https://doi.org/10.1177/0883073810379640](https://psycnet.apa.org/doi/10.1177/0883073810379640)

Parent, J., McKee, L. G., Rough, J. N., & Forehand, R. (2016). The association of parent mindfulness with parenting and youth psychopathology across three developmental stages. *Journal of Abnormal Child Psychology*, *44*(1), 191-202. <https://doi.org/10.1007/s10802-015-9978-x>

Parsons, T. D., Riva, G., Parsons, S., Mantovani, F., Newbutt, N., Lin, L., Venturini, E., & Hall, T. (2017). Virtual reality in pediatric psychology. *Pediatrics*, *140*(Supplement 2), S86-S91. <https://doi.org/10.1542/peds.2016-1758I>

Pfiffner, L. J., & Haack, L. M. (2014). Behavior management for school-aged children with ADHD. *Child and Adolescent Psychiatric Clinics*, *23*(4), 731-746. <https://doi.org/10.1016/j.chc.2014.05.014>

|  |
| --- |

Polanczyk, G., De Lima, M. S., Horta, B. L., Biederman, J., & Rohde, L. A. (2007). The worldwide prevalence of ADHD: A systematic review and metaregression analysis. *American Journal of Psychiatry*, *164*(6), 942-948. <https://ajp.psychiatryonline.org/doi/10.1176/ajp.2007.164.6.942>

Rajeh, A., Amanullah, S., Shivakumar, K., & Cole, J. (2017). Interventions in ADHD: A comparative review of stimulant medications and behavioral therapies. *Asian Journal of Psychiatry*, *25*, 131-135. <http://dx.doi.org/10.1016/j.ajp.2016.09.005>

Rommelse, N. N. J., Van der Stigchel, S., & Sergeant, J. A. (2008). A review on eye movement studies in childhood and adolescent psychiatry. *Brain and Cognition, 68*(3), 391-414. <https://doi.org/10.1016/j.bandc.2008.08.025>

Schellack, N., Meyer, J. C., & Chigome, A. K. (2019). The management of attention-deficit hyperactivity disorder in children: Updated 2019. *South African Pharmaceutical Journal*, *86*(5), 17-27. <http://sapj.co.za/index.php/SAPJ/article/view/2756>

Sekaninova, N., Mestanik, M., Mestanikova, A., Hamrakova, A., & Tonhajzerova, I. (2019). Novel approach to evaluate central autonomic regulation in attention deficit/hyperactivity disorder (ADHD). *Physiological Research*, *68*, 531-545. <https://doi.org/10.33549/physiolres.934160>

Sjöwall, D., Roth, L., Lindqvist, S., & Thorell, L. B. (2013). Multiple deficits in ADHD: executive dysfunction, delay aversion, reaction time variability, and emotional deficits. *Journal of Child Psychology and Psychiatry*, *54*(6), 619-627. <https://doi.org/10.1111/jcpp.12006>

Tarver, J., Daley, D., & Sayal, K. (2014). Attention‐deficit hyperactivity disorder (ADHD): an updated review of the essential facts. *Child: Care, Health and Development*, *40*(6), 762-774. <https://doi.org/10.1111/cch.12139>

Theule, J., Wiener, J., Tannock, R., & Jenkins, J. M. (2013). Parenting stress in families of children with ADHD: A meta-analysis. *Journal of Emotional and Behavioral Disorders*, *21*(1), 3-17. <https://doi.org/10.1177%2F1063426610387433>

Wang, M., & Reid, D. (2011). Virtual reality in pediatric neurorehabilitation: attention deficit hyperactivity disorder, autism and cerebral palsy. *Neuroepidemiology*, *36*(1), 2-18. <https://doi.org/10.1159/000320847>

Zachor, D., Hodgens, B., & Patterson, C. (2009). Treatment of attention-deficit/hyperactivity disorder (ADHD). In J.L.Matson, F. Andrasik, & M.L. Matson(eds.), *Treating childhood psychopathology and developmental disabilities* (pp.139-181). Springer. <https://doi.org/10.1007/978-0-387-09530-1_6>

**Appendix Α**

**Parents’ Questionnaires:**

1. Demographic data
2. Parenting Styles and Dimensions Questionnaire for Greek fathers (PSDQ)
3. Parenting Styles and Dimensions Questionnaire for Greek mothers (PSDQ)
4. Parenting Stress Index-Short form (PSI-SF)
5. Tool to Measure Parenting Self-Efficacy (TOPSE)
6. ADHD-IV Rating Scale-Greek version

**Children’s Questionnaires:**

1. Demographic data
2. Psychosocial Adjustment Test (Greek test)
3. Children’s Perceptions of Parenting Styles and Dimensions Questionnaire for Greek fathers
4. Children’s Perceptions of Parenting Styles and Dimensions Questionnaire for Greek mothers
5. Parental Acceptance/Rejection Questionnaire-Short Form (PARQ-Child) for fathers and mothers

**Appendix B**

1. Participants’ Information and Consent Form for Parents of Experimental Group
2. Participants’ Information and Consent Form for Students of Experimental Group
3. Participants’ Information and Consent Form for Parents of Control Group
4. Participants’ Information and Consent Form for Students of Control Group
